# Multicomponent Electrosynthesis of Enaminyl Sulfonates Starting from Alkylamines, SO_2_, and Alcohols

**DOI:** 10.1021/acs.orglett.4c04746

**Published:** 2025-01-27

**Authors:** Florian A. Breitschaft, Alicia L. Saak, Christian Krumbiegel, Aloisio de A. Bartolomeu, Thomas Weyhermüller, Siegfried R. Waldvogel

**Affiliations:** † Max-Planck-Institute for Chemical Energy Conversion, Stiftstraße 34−36, 45470 Mülheim an der Ruhr, Germany; ‡ Department of Chemistry, Johannes Gutenberg University, Duesbergweg 10−14, 55218 Mainz, Germany; § Karlsruhe Institute of Technology, Institute of Biological and Chemical Systems − Functional Molecular Systems (IBCS FMS), Kaiserstraße 12, 76131 Karlsruhe, Germany

## Abstract

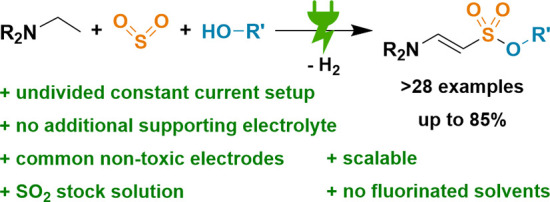

An electrochemical one-pot synthesis of enaminyl sulfonate esters was established, featuring a quasidivided cell under constant current conditions. The multicomponent reaction utilizes simple and readily available alkylamines and an easy-to-use stock solution of SO_2_ and alcohols. Omission of additional supporting electrolyte through in-situ-generated monoalkylsulfite facilitates the downstream processing. A diverse scope with more than 28 examples and yields up to 85% as well as a 20-fold scale-up reaction prove the feasibility of this novel protocol.

The β-amino sulfonyl functionality is a prevalent motif in many pharmaceuticals or natural products. The simplest representative of this class is the nonproteinogenic ammonium sulfonate taurine (Figure 1). Biosynthesized from cysteine, taurine provides numerous physiological activities, ranging from cytoprotection^1^ and neurotransmitter^2^ to its highly discussed role as a (semi)essential nutrient^3^ and its application as a therapeutic.^4^ Taurine-derived taurocholic acid is naturally occurring in the bile of mammals and has found application as a choleretic.^5^ Structurally related, the β-amino sulfone apremilast (Otezla, Amgen, Figure 1) is one of the most-sold pharmaceuticals worldwide, accounting for more than 2 billion USD in sales in 2021.^6^ As a phosphodiesterase 4 (PDE 4) inhibitor, apremilast is administered in cases of severe psoriasis and psoriatic arthritis. The penicillin-derived drug sulbactam, also exhibiting a β-amino sulfone motif, is applied together with β-lactam antibiotics to inhibit the effects of β-lactamase, increasing the efficiency of the antibiotic drastically.^7^ TSAO-T (Figure 1), a spirocyclic enaminyl sultone, is able to inhibit HIV-1 reverse transcriptase in a highly selective and noncompetitive way,^8^ rendering it a potential lead structure for the development of anti-AIDS medications.^9^ Installation of these moieties can be achieved by conventional chemistry including aza-Michael addition,^10^ cycloaddition,^11^ Knoevenagel reaction,^12^ Horner-Wadsworth-Emmons reaction,^13^ or the condensation of functionalized sp^3^ carbons with formanilides.^14^ Recently, sodium sulfonates^15^ (Scheme 1) or sulfonyl hydrazides^16^ have emerged for the generation of vinyl sulfones, requiring an additional oxidant in stoichiometric amounts. Electrochemistry on the other hand uses electric current as a green oxidant,^17^ therefore omitting toxic and/or expensive catalysts while being inherently safe.^18^ Electrosynthetic methods for the construction of enaminyl sulfones have been developed by several groups (Scheme 1), utilizing different aryl sulfonyl precursors.^19^ However, this restricts the resulting products to sulfones, while enaminyl sulfonates are hardly accessed and seem to be underexplored. Sulfur fluoride exchange chemistry may be used^20^ but is crucially limited by the commercial availability of suitable sulfonyl fluorides. Utilizing the inexpensive chemical feedstock sulfur dioxide as a central building block,^21^ we report a novel dehydrogenative electrochemical multicomponent reaction for the construction of enaminyl sulfonates starting from simple amines, SO_2_, and alcohols (Scheme 1). This approach circumvents the need for prefunctionalization and allows for the direct incorporation of the pollutant sulfur dioxide into value-added products like sulfonates,^22^ sulfonamides,^23^ or sulfamides.^24^ As a source of SO_2_, we employ readily available and easy-to-use stock solutions, minimizing waste and facilitating downstream processing, a key step for the translation into technical application.^23b,25^

**Figure 1:**
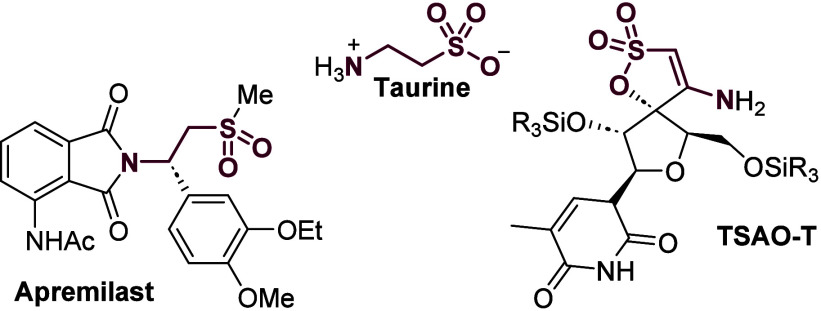
Prominent compounds containing the β-amino sulfonyl motif.

**Scheme 1:**
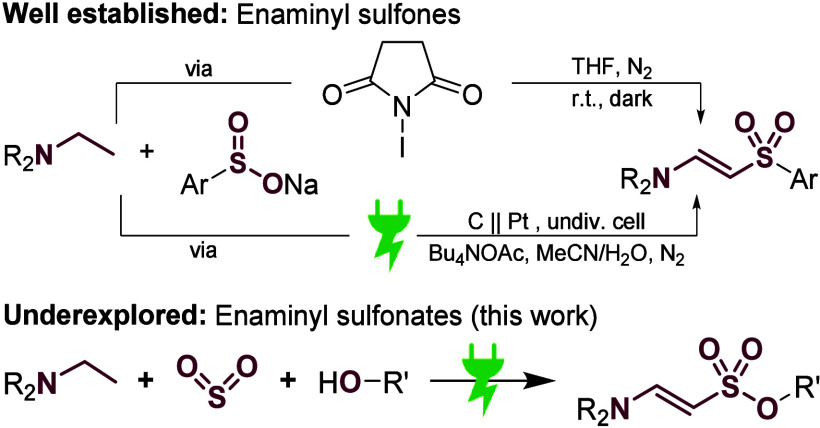
Selected Methods for the Construction of the β-Enaminyl Sulfonyl Moiety

The initial reactivity was discovered using *N*,*N*-diisopropylamine (**1a**), SO_2_ stock solution, and neopentyl alcohol (**2a**) employing a graphite anode and stainless-steel cathode in an undivided cell under galvanostatic conditions (Table 1). Neopentanol was chosen due to the enhanced stability as sulfonate.^26^ With the help of 1,8-diazabicyclo[5.4.0]undec-7-ene (DBU), **3a** was obtained in an ^1^H NMR yield of 24% (Entry 1). Increasing the stoichiometry of the reactants (Entry 2) as well as altering the geometry of the cathode from a plate to a thin wire improved the yield to 50% (Entry 3). This setup, commonly known as a quasidivided cell,^27^ can help to prevent undesired counter reactions,^28^ since the electron transfer becomes diffusion-limited, leading to mostly solvent degradation. Screening of anode materials (Entries 4 - 6 and Supporting Information) showed the best results with an inexpensive and readily available graphite foil (Sigraflex). Testing of different bases (Entries 7–9 and Supporting Information) showed no improvement. Using a design-of-experiments study^29^ (2^4–1^-design plan with star points, see Supporting Information), the stoichiometry of alcohol and base, the current density, and amount of applied charge were optimized, which resulted in an enhanced yield of 65% (Entry 10). Pretreating the Sigraflex electrode in MeCN prior to use resulted in minor swelling, and the qNMR yield of **3a** was increased to 70%, of which 61% could be isolated (Entry 11). The pretreating is believed to improve the diffusion of the substrates into the porous electrode.

**Table 1:**
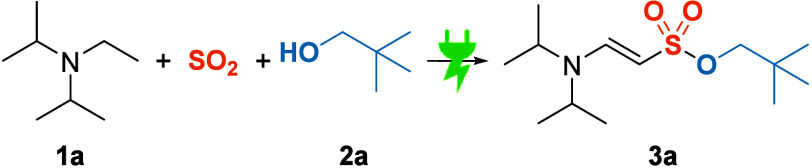
Optimization of Reaction Conditions^a^

Entry	**2a** [eq.]	Base	Anode material	Yield^b^
1	2.0	DBU^c^	Graphite	24%^d^
2	4.0	DBU	Graphite	41%^d^
3	4.0	DBU	Graphite	50%
4	4.0	DBU	Glassy Carbon	16%
5	4.0	DBU	BDD	18%
6	4.0	DBU	Sigraflex	55%
7	4.0	DBN	Sigraflex	51%
8	4.0	TMG	Sigraflex	49%
9	4.0	2,6-Lutidine	Sigraflex	0%
10	5.2	DBU^e^	Sigraflex	65%^f^
11	5.2	DBU^e^	Sigraflex	70%^f^,^g^ (61%)

a Conditions: **1a** (500 μmol, 1 equiv, 0.1 M), SO_2_ (1.5 × [equiv **2a**]), **2a**, base (8.0 equiv), MeCN, anode||stainless-steel wire, 40 mA/cm^2^, 10 *F*, r.t.

b Yield determined by ^1^H NMR with 1,3,5-trimethoxy-benzene as internal standard. Isolated yield in parentheses.

c 5.0 equiv.

d Planar stainless-steel cathode.

e 9.0 equiv.

f 67.5 mA/cm^2^, 11.5 *F*.

g Pretreating of Sigraflex electrode in acetonitrile 2 h before usage.

With these optimized conditions in hand, we explored the scope of our newly discovered reactivity using various alkyl and aromatic amines as well as amides (Scheme 2). Ethylamines bearing isopropyl (**3a**) or cyclohexyl moieties (**3b** and **3c**) gave good yields ranging from 51% to 61%. Highly hindered and rigid *N*-ethyl-2,2,6,6-tetramethylpiperidine was sulfonylated in an excellent yield of 85% (**3d**). Its structure was verified by single-crystal X-ray analysis (CCDC 2407541). Investigating different alkyl chain lengths, we found a sharp decline in yield when switching from ethyl (**3e**, 72%) to propyl (**3f**, 15%) or butyl groups (**3g**, 10%). This agrees with previous reports suggesting a kinetic preference of *n*-alkylamines to dehydrogenate in the terminal position.^30^ Additionally, numerous dealkylated byproducts were observed for **3f** and **3g** (see Supporting Information for details). NMR experiments confirmed the displayed (*E*)-configuration. While *N*-ethylpyrrolidine lead to overoxidation (see Supporting Information), nitrogen heterocycles could be applied with our methodology. In these cases, a competing reaction between the *exo*- and *endo*-product was observed. Product mixtures were obtained for the sulfonylation of *N*-ethylpiperidine (**3h**, 21%) and *N*-ethylazepane (**3i**, 14%) with *exo*/*endo* ratios of 4/1 and 1/1, respectively. Sulfonylation of unsaturated *N*-functionalized heteroaromatics occurred not at the ethyl group but solely on the 3-position of the aromatic ring (**3j**, from *N*-ethylpyrrole, and **3k**, from *N*-ethylindole, both 35%). Since the lone pair of the N atom is part of the aromatic system, removal of an electron leads to a more stable intermediate rather than the exocyclic cation. While the electrolysis of aniline derivatives often results in polymerization and the formation of aniline black,^31^ we were pleased to see that *N*,*N*-diethylaniline was sulfonylated in an acceptable yield of 27% (**3l**). By substitution of the *para*-position by a methyl group, yield could be increased to 55% (**3m**). Surprisingly, *N*-acetylation lead to no conversion of the starting material (see Supporting Information for examples); not even Shono-type reactivity^32^ was observed. However, starting from *N*-vinyl compounds and therefore skipping the oxidation from amide to enamide reestablished the desired reactivity. *N*-Vinylamides are common motifs frequently employed in polymer chemistry.^33^ We achieved a good yield of 67% for the sulfonylation of *N*-methyl-*N*-vinylacetamide (**3n**) and moderate yields of 46% (**3o**) and 49% (**3p**) for the two cyclic analogues. Lastly, after the solvent was changed to benzonitrile due to limited solubility, *N*-vinylcarbazole could be sulfonylated in an acceptable yield of 28% (**3q**).

**Scheme 2:**
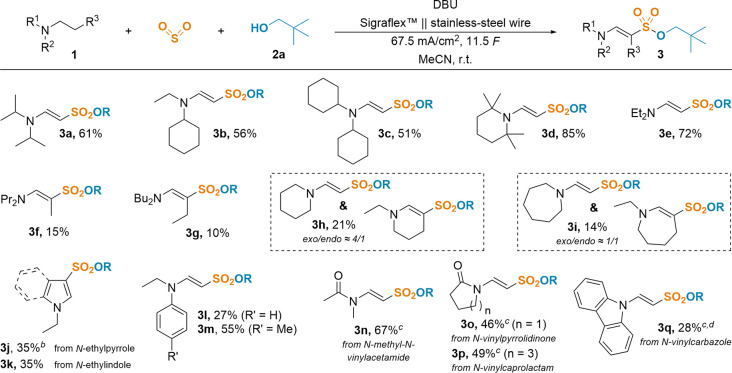
Scope of Amines, Anilines, and Vinyl Amides Isolated yields displayed. R = neopentyl. Conditions: amine **1** (500 μmol, 1 equiv, 0.1 M), SO_2_ (7.8 equiv), **2a** (5.2 equiv), DBU (9.0 equiv), Sigraflex||stainless-steel wire, 67.5 mA/cm^2^, 11.5 *F*, r.t. 17.25 *F*. 5.75 *F*. PhCN as solvent.

Following these promising results, we explored the scope of the alcohols (Scheme 3). Besides neopentanol (**3d**, 85%), simple primary alkyl alcohols like methanol or ethanol yielded 64% (**4a**) and 69% (**4b**), respectively. Even very apolar 1-decanol could be converted into the respective sulfonate ester in a satisfying yield of 69% (**4c**). With racemic 2-methylbutanol, an excellent yield of 85% was achieved (**4d**). Using secondary alcohols resulted in yields of 58% (**4e**, with isopropanol), 67% (**4f**, with cyclohexanol), and 58% (**4g**) for the sterically demanding 2-adamantol. Tertiary alcohols such as *tert*-butanol however could not be converted with our methodology (see Supporting Information for limitations), most probably due to their bulkiness. Similar observations were made in previous projects.^22^ Demonstrating the range of applicable alcohols, an acceptable yield of 38% was achieved for methyl lactate (**4h**). Since amides do not interfere with the desired reactivity, two *N*-Boc-protected aminoalcohols were tested, which resulted in yields of 62% (**4i**) and 47% (**4j**), respectively. Even labile 3-cyclohexenol was converted into the corresponding sulfonate, albeit with a lowered yield of 21% (**4k**). Unfortunately, the use of phenols such as 2,4-dichlorophenol did not yield the desired product but resulted in the formation of dimers instead (see Supporting Information). Using secondary amines instead of alcohols, we could isolate only minor amounts of the desired sulfonamides (see Supporting Information for examples). Since an ^1^H NMR sample of the crude reaction mixture indicated a moderate yield of 44%, we suspect degradation of the enaminyl sulfonamide during column chromatography.

**Scheme 3:**
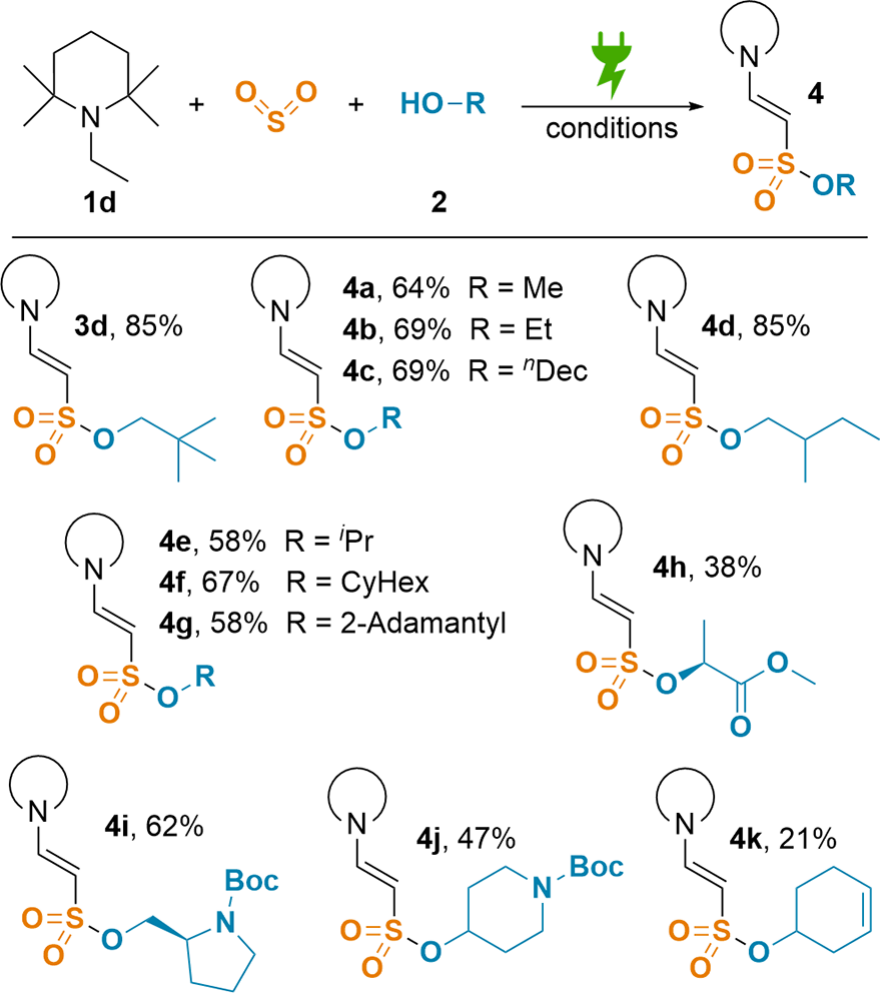
Scope of Alcohols Conditions: **1d** (500 μmol, 1 equiv, 0.1 M), SO_2_ (7.8 equiv), alcohol **2** (5.2 equiv), DBU (9.0 equiv), Sigraflex||stainless-steel wire, 67.5 mA/cm^2^, 11.5 *F*, r.t. Isolated yields displayed.

To showcase robustness and scalability of our dehydrogenative sulfonylation, enaminyl sulfonate **3d** was synthesized in a gram scale reaction (Scheme 4, 20-fold scale-up, see Supporting Information). Herein, we observed a similar ^1^H NMR yield (84% vs 90% in the small scale) and only a minor decline in isolated yield (76%, 2.42 g vs 85%). A similar scale-up experiment with *N*-methyl-*N*-vinylacetamide yielded 84% of **3n** (compared to 67% in the small-scale, see Supporting Information for details).

**Scheme 4:**
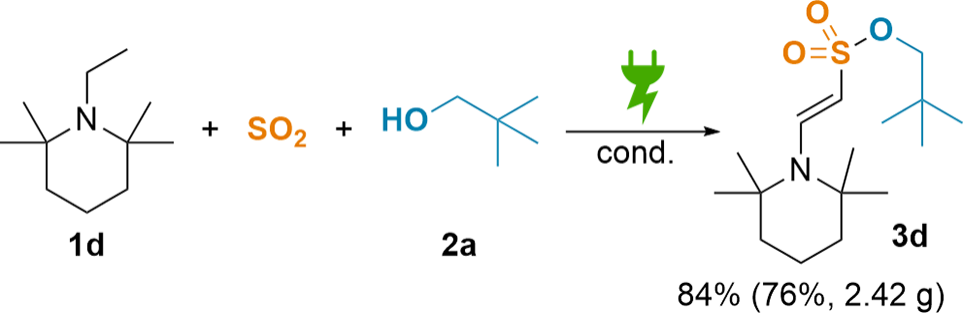
Gram Scale Synthesis of **3d** Conditions: **1d** (10.0 mmol, 1 equiv, 0.1 M), SO_2_ (7.8 equiv), **2a** (5.2 equiv), DBU (9.0 equiv), Sigraflex||stainless-steel wire, 67.5 mA/cm^2^, 11.5 *F*, r.t. Yield determined via ^1^H NMR. Isolated yield in parentheses.

To gain insight toward a possible reaction mechanism, several control experiments were conducted (Table 2). As expected, no signs of the desired product were detected when omitting electric charge or base (Entries 1 and 2). By addition of radical scavengers 2,2,6,6-tetramethylpiperidinyloxyl (TEMPO, Entry 3) or butylated hydroxytoluene (BHT, Entry 4), the yield dropped significantly but did not diminish completely. This may be attributed to the fact that TEMPO as well as BHT are readily oxidized, which competes with oxidation of the amine substrate.^34^ With BHT, *N,N*-diisopropylvinylamine (which, according to literature reports,^30b,35^ is only stable below −20 °C for prolonged time) and a neopentyl sulfonyl species could be trapped and detected via GC/MS, respectively (see Supporting Information). Unsurprisingly, cyclic voltammetry experiments (see Supporting Information) showed the early oxidation of the amine substrate, while the products and alkoxy sulfonyl intermediate were stable toward oxidation.

**Table 2:** Control Experiments

Entry	Deviation from the standard conditions^a^	Yield^b^
1	No charge passed	0%
2	No base (+ 0.1 M Bu_4_NBF_4_ for conductivity)	0%
3	+ TEMPO (3 equiv)	38%
4	+ BHT (3 equiv)	20%

a **1a** (500 μmol, 1 equiv, 0.1 M), SO_2_ (7.8 equiv), **2a** (5.2 equiv), DBU (9.0 equiv), Sigraflex||stainless-steel wire, 67.5 mA/cm^2^, 11.5 *F*, r.t.

b Yield determined via ^1^H NMR.

Based on these results and previous literature reports, we propose the following mechanism (Scheme 5): First, the tertiary alkylamine substrate **1** gets oxidized at the anode to the enamine **A**, releasing two protons in the process, which are intercepted by an excess of base. Electrochemical oxidation of amines to enamines is well-established in the literature^36^ and has been used in similar transformations for the construction of enaminyl sulfones.^19^ Subsequently, **A** is oxidized again in a one-electron fashion,^37^ yielding radical cation **B**, which is stabilized by allylic resonance structure **C**. Multiple literature reports^38^ have identified **C** as the predominant form of the enamine radical cation, which is better described as an α-imino radical.^39^*O*-Monoalkylsulfite **D** can be formed in situ by insertion of SO_2_ into the O–H bond of the alcohol **2**, with DBU shifting the equilibrium toward the deprotonated species. Such intermediates have been known for a long time^40^ and put to synthetic use on multiple occasions already.^22,23^ They also provide the conductivity necessary for electrolysis, which is why the need for an additional supporting electrolyte is circumvented. **D** adds to resonance structure **C**, forming the *S*-centered radical **E**. Noteworthily, we did not find any evidence for a nucleophilic *O*- or *S*-attack of **D** to the iminium carbon of ion **C**. On the other hand, the ability of SO_2_-derived species to trap free radicals and the extraordinary stability of the resulting *S*-centered radicals is well-known.^21,41^ Subsequently, **E** undergoes another anodic oxidation to **F**, and deprotonation through an excess of base finally affords the desired enaminyl sulfonate. As a counter reaction, the high current density on the stainless-steel wire leads mostly to solvent degradation and hydrogen evolution, as evidenced by the formation of bubbles observed during the scale-up experiment.

**Scheme 5:**
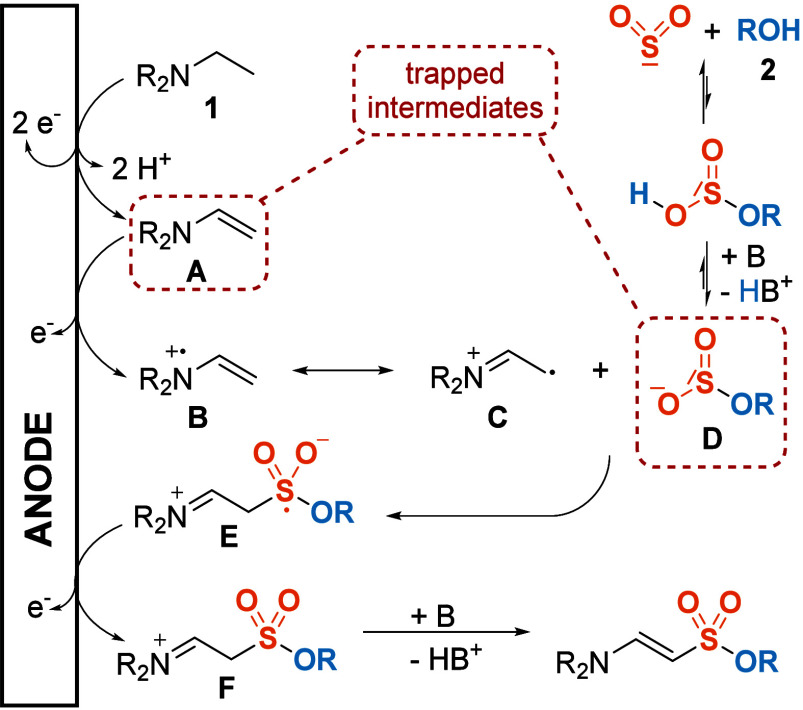
Proposed Reaction Mechanism

In summary, we developed a new electrochemical dehydrogenative multicomponent reaction affording enaminyl sulfonates from abundant alkylamines, SO_2_, and alcohols. The process features inexpensive electrode materials and utilizes a simple quasidivided setup under galvanostatic conditions. An extensive scope of more than 28 examples with yields up to 85% as well as a gram-scale reaction demonstrates the feasibility of this first-of-its-kind transformation. Our one-pot method opens a new and straightforward pathway for the construction of up-to-this-date underexplored enaminyl sulfonate esters.

## Data Availability

The data underlying this study are available in the published article and its Supporting Information.

## Supporting Information Available

Experimental details, spectra of isolated compounds, and crystallographic data. Additional references are cited herein. (PDF)
