# Supplementary material for: Multicomponent Electrosynthesis of Enaminyl Sulfonates Starting from Alkylamines, SO2, and Alcohols
Source: Org Lett. 2025 Jan 27;27(5):1210–5. doi: 10.1021/acs.orglett.4c04746 (PMC11812012; doi:10.1021/acs.orglett.4c04746)
Supplement: Supplementary file 1 — ol4c04746_si_001.pdf [file ol4c04746_si_001.pdf]

Supporting Information

Multicomponent Electrosynthesis of Enaminyl Sulfonates starting from Alkylamines, SO_2 and Alcohols

Florian A. Breitschaft, Alicia L. Saak, Christian Krumbiegel, Aloisio de A. Bartolomeu, Thomas Weyhermüller and Siegfried R. Waldvogel

Table of Contents

1 GENERAL ASPECTS 3

1.1 General Information 3
1.2 Instruments and Analytical Methods 3
1.3 Electrochemical Setup 4

2 EXPERIMENTAL PROCEDURES 6

2.1 Preparation of the SO_2 stock solution 6
2.2 Determination of the SO_2 concentration of the stock solution 6
2.3 General protocol for the synthesis of alkyl enaminylsulfonates (GP1) 6
2.4 General protocol for the optimization of reaction conditions (GP2) 6
2.5 Protocol for the large-scale synthesis 7

3 OPTIMIZATION 9

4 CONTROL EXPERIMENTS 13

5 CYCLOVOLTAMMETRY STUDIES 14

6 COMPOUND CHARACTERIZATION 16

6.1 Neopentyl (*E*)-2-(diisopropylamino)ethene sulfonate (3a) 16
6.2 Neopentyl (*E*)-2-(cyclohexyl(ethyl)amino)ethene sulfonate (3b) 16
6.3 Neopentyl (*E*)-2-(dicyclohexylamino)ethene sulfonate (3c) 16
6.4 Neopentyl (*E*)-2-(2,2,6,6-tetramethylpiperidinyl)ethene sulfonate (3d) 17
6.5 Neopentyl (*E*)-2-(diethylamino)ethene sulfonate (3e) 17
6.6 Neopentyl (*E*)-1-(dipropylamino)prop-1-ene-2-sulfonate (3f) 17
6.7 Neopentyl (*E*)-1-(dibutylamino)but-1-ene-2-sulfonate (3g) 18
6.8 Neopentyl (*E*)-2-(piperidinyl)ethene sulfonate (3h) 18
6.9 Neopentyl (*E*)-2-(azepanyl)ethene sulfonate (3i) 18
6.10 Neopentyl *N*-ethyl-1H-pyrrole-3-sulfonate (3j) 19
6.11 Neopentyl *N*-ethyl-1H-indole-2-sulfonate (3k) 19
6.12 Neopentyl (*E*)-2-(ethyl(phenyl)amino)ethene sulfonate (3l) 19
6.13 Neopentyl (*E*)-2-(ethyl(p-tolyl)amino)ethene sulfonate (3m) 20
6.14 Neopentyl (*E*)-2-(*N*-methylacetamido)ethene sulfonate (3n) 20
6.15 Neopentyl (*E*)-2-(2-oxopyrrolidinyl)ethene sulfonate (3o) 20
6.16 Neopentyl (*E*)-2-(2-oxopyrrolidinyl)ethene sulfonate (3p) 21
6.17 Neopentyl (*E*)-2-(9H-carbazol-9-yl)ethene sulfonate (3q) 21
6.18 Methyl (*E*)-2-(2,2,6,6-tetramethylpiperidinyl)ethene sulfonate (4a) 21
6.19 Ethyl (*E*)-2-(2,2,6,6-tetramethylpiperidinyl)ethene sulfonate (4b) 22
6.20 ^{n}Decyl (*E*)-2-(2,2,6,6-tetramethylpiperidinyl)ethene sulfonate (4c) 22
6.21 2-Methylbutyl (*E*)-2-(2,2,6,6-tetramethylpiperidinyl)ethene sulfonate (4d) 22
6.22 Isopropyl (*E*)-2-(2,2,6,6-tetramethylpiperidinyl)ethene sulfonate (4e) 22
6.23 Cyclohexyl (*E*)-2-(2,2,6,6-tetramethylpiperidinyl)ethene sulfonate (4f) 23
6.24 Adamantan-2-yl (*E*)-2-(2,2,6,6-tetramethylpiperidinyl)ethene sulfonate (4g) 23
6.25 *S*-Methyllactyl (*E*)-2-(2,2,6,6-tetramethylpiperidinyl)ethene sulfonate (4h) 23
6.26 *N*-Butyloxycarbonylprolinyl (*E*)-2-(2,2,6,6-tetramethylpiperidinyl)ethene sulfonate (4i) 24
6.27 Tert-butyl (*E*)-4-(((2-(2,2,6,6-tetramethylpiperidinyl)vinyl)sulfonyl)oxy)piperidine carboxylate (4j) 24
6.28 Cyclohex-3-enyl (*E*)-2-(2,2,6,6-tetramethylpiperidin-1-yl)ethene sulfonate (4k) 24
6.29 (*E*)-4-((2-(2,2,6,6-tetramethylpiperidinyl)vinyl)sulfonyl)morpholine (5a) 25
6.30 (1*R*,5*S*)-8-(((*E*)-2-(2,2,6,6-tetramethylpiperidinyl)vinyl)sulfonyl)-8-azabicyclo[3.2.1]octane (5b) 25
6.31 Limitations of the Scope/Unsuccessful substrates 26

7 CRYSTALLOGRAPHIC DATA 27

8 NMR SPECTRA 28

9 AUTHOR CONTRIBUTIONS 59

10 REFERENCES 59

1 General aspects

1.1 General Information

If not stated otherwise, all reactions were performed under ambient conditions and chemicals in analytical grade were used as purchased without further purification. Cyclohexane and ethyl acetate used for column chromatography were purchased in HPLC-grade. Acetonitrile was commercially obtained in LCMS-grade and stored over molecular sieves (3 Å) prior use.

1.2 Instruments and Analytical Methods

Chromatography

Thin layer chromatography for reaction monitoring was performed using DC Kieselgel 60 F_{254} on aluminium plates (*Merck KGaA,* Darmstadt, Germany). An UV lamp (λ = 254 nm, UV-4 S/L, *Herolab GmbH Laborgeräte*, Wiesloch, Germany) and potassium permanganate solution (3 g $KMnO_4$, 20 g K_2CO_3, 5 mL NaOH (5%), 300 mL H_2O) were used for substance detection. Preparative column chromatography was performed on prepacked puriFlash™ silica columns (15 µm or 30 µm, PF-15SIHP-F0012, PF-15SIHP-F0025, PF-25SIHC-F0025, PF-15SIHP-F0040, PF-25SIHC-F0120 *Interchim*, Montlucon Cedex, France) using a puriFlash™-System (puriFlash™ XS520Plus, *Interchim*, Montlucon Cedex, France) with an integrated UV detector.

High Resolution Mass Spectrometry

Mass spectra *via* electrospray-ionization (ESI+) were recorded using a Q Exactive™ mass spectrometer (*Thermo Fischer Scientific*™, Waltham, USA). Mass spectra *via* electron-ionization (EI) were recorded using a Q Exactive™ GC Orbitrap™ GC-MS/MS (*Thermo Fischer Scientific*™, Waltham, USA) equipped with an XTI-5 column (*Restek GmbH*, Bad Homburg v. d. Höhe, Deutschland).

X-Ray Crystallography

Measurement of the single-crystal structure was carried out on a Bruker D8 Venture Kappa Diffractometer equipped with IµS3 diamond Mo-source (50 kV, 1.4 mA; Mo-K-alpha radiation, lambda = 0.71073 Å), Oxford cryostream 800 cooler, Incoatec Helios mirror optics and a Photon III detector.

Gas Chromatography (GC)

Analysis of crude reaction mixtures and purified products were performed using a GC-2030 (*Shimadzu*, Kyoto, Japan) equipped with a flame ionization detector (FID) and a quartz capillary column HI-5MS (*Avantor VWR*, Radnor, USA) with following specification: length of 30 m, inner diameter of 0.25 mm and a stationary phase ((5%-phenyl)dimethylsiloxane) of 0.25 µm thickness. Hydrogen was used as carrier gas with a constant velocity of 40 cm/s. Measurements were performed at an injector temperature of 270 °C and a detector temperature of 320 °C, starting at 50 °C (holding for 1 min) and heating to 300 °C (holding for 4.71 min) with a temperature ramp of 17.5 °C/min (method: 2_medium, total program time: 20.0 min).

Gas Chromatography coupled with Mass Spectrometry (GC/MS)

Analysis of crude reaction mixtures and purified products were performed using a GCMS QP2010SE (*Shimadzu*, Kyoto, Japan) equipped with an electron ionization (EI) source and a quadrupole mass analyzer. A quartz capillary column HI-5MS (*Avantor VWR*, Radnor, USA) with the following specification was used: length of 30 m, inner diameter of 0.25 mm and a stationary phase [(5%-phenyl)-dimethylsiloxane] of 0.25 µm thickness. Helium was used as carrier gas with a constant velocity of 30 cm/s. The GC temperature ramp started at 50 °C (holding for 1 min) and heating to 300 °C (holding for 4.71 min) with a temperature ramp of 17.5 °C/min (method: 2_medium, total program time: 20.0 min). Measurements were performed at an injector temperature of 270 °C and a temperature of the EI source of 250 °C.

Nuclear Magnetic Resonance (NMR) Spectroscopy

^{1}H NMR and $^{13}C\{^{1}H\}$ NMR spectra were recorded at 25 °C on a Bruker AVANCE III HD 500 MHz NMR spectrometer with a Bruker Prodigy probe (*Bruker BioSpin GmbH*, Rheinstetten, Germany) using $CDCl_3$ or CD_3CN as deuterated solvent. All chemical shifts are reported in δ-scale as parts per million [ppm] (multiplicity, coupling constant J, number of protons), relative to the solvent residual peaks as the internal standard. Coupling constants J are given in Hertz [Hz]. Besides ^{1}H and ^{13}C experiments, the 2D techniques $^{1}H,^{1}H$-COSY, $^{1}H,^{13}C$-HSQC, $^{1}H,^{13}C$-HMBC and $^{1}H,^{1}H$-NOESY were used assisting to assign the signals. Determination of stereochemistry (*E/Z*-isomerism) was done using $^{1}H,^{1}H$-^{3}J vicinal coupling, $^{1}H,^{1}H$-NOESY or $^{3}J_{C,H}$-HMBC-Coupling. The following abbreviations were used to describe the signals: s (singlet), d (doublet), t (triplet), q (quartet), pent (pentet), sext (sextett), hept (heptet), m (multiplet), br (broad signal). The spectra obtained were evaluated with MestReNova 14 (*Mestrelab Research S.L.*, Spain).

Cyclic Voltammetry (CV) Measurements

Cyclic voltammetry was performed using a Metrohm 663 VA Stand equipped with an Autolab type III potentiostat (*Metrohm AG*, Herisau, Switzerland). WE: glassy carbon electrode (*d* = 2 mm); CE: glassy carbon rod; RE: Ag/$AgNO_3$; Scan rate *v* = 100 mV/s. Electrolyte: 0.1 M Bu_4NBF_4 in acetonitrile; *c*(substrate) = 10 mmol/L; *c*(SO_2) = 40 mmol/L; *V* = 5 mL. All solutions except for the ones containing SO_2 have been deoxygenated by bubbling with argon for 10 min prior to measurement. Electrodes have been thoroughly rinsed before and after each measurement with acetone and acetonitrile. The glassy carbon working electrode was polished with

alumina paste (1 µm followed by 0.1 µm) and sonicated for 5 min each in between measurements. All data is displayed against the half-wave potential of ferrocene/ferrocenium redox couple (FcH/FcH^+; -0.10 V vs. $Ag/AgNO_3$) as internal reference. Oxidation potentials are marked and displayed as the half-wave potential of the respective peak.

1.3 Electrochemical Setup

Galvanostat

Electrochemical reactions were carried out using a multichannel galvanostat HMP4040 (*Rohde & Schwarz*, München, Germany). The cells used for screening or large-scale batch reactions are described below (Figure S1).

Screening and small-scale batch reactions

Screening reactions were carried out in undivided/quasi-dived Teflon™ cells with a volume of 5 mL equipped with two electrodes (interelectrode gap: 6 mm) and a round, cross-shaped stirring bar. The described system is commercially available as IKA Screening System Package (*IKA™ Werke GmbH & Co. KG*, Staufen, Germany).

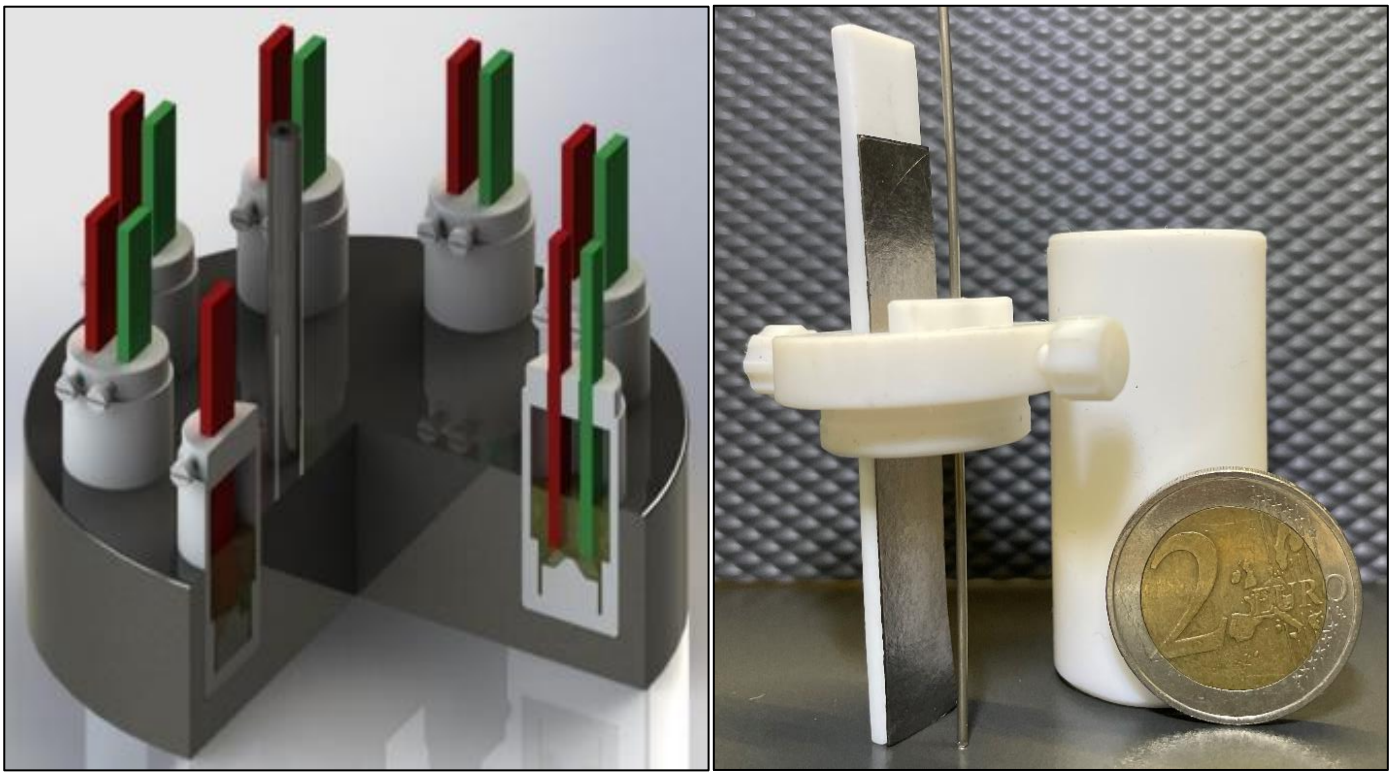

Figure S1: Undivided screening setup as available through IKA[1] (left) and quasi-divided (right) cell with Sigraflex™ anode and stainless-steel wire cathode. Diameter of the coin 25.75 mm.

The screening was carried out using various electrode materials (7 cm × 1 cm × 0.3 cm). Graphite electrodes were sanded using 600 grit followed by 1000 grit sandpaper and cleaned with acetonitrile prior to use. BDD electrodes were electrochemically treated (j = 50 mA/cm^2) in 20% (*v*/*v*) sulfuric acid for 10 min before use. Sigraflex™, an inexpensive graphite foil that is frequently used for sealing or heat transfer purposes,[2] was cut into electrodes (7 cm × 1 cm × 0.02 cm) and pretreated in the corresponding solvent for at least 2 h prior to use and fixed to PTFE supports. The anodes were immersed 1.8 cm (planar cathode) or 1.6 cm (wire cathode) into the electrolyte. This resulted in a geometric active electrode area of 1.8 cm^2 or 1.6 cm^2 used for electrolysis. The stainless-steel wire (d = 0.1 cm; l = 7 cm) was fixated using an inhouse-made PTFE adapter.

Table S1: Electrode materials, purity, and their supplier.

Entry	Electrode Material	Specification	Supplier
1	Boron-doped diamond	15 µm boron-doped diamond on silicon support	*CONDIAS GmbH*, Itzehoe, Germany
2	Glassy Carbon	Sigradur G	*HTW*, Thierhaupten, Germany
3	Graphite	highly isostatic, V2100	*SGL Carbon*, Bonn, Germany
4	Graphite foil (Sigraflex™)	F02012Z	*SGL Carbon*, Meitingen, Germany
5	Stainless-steel	1.4571	various suppliers (metal traders)

Scale-up Cell

The scale-up experiment was performed in a jacketed undivided glass cell with a volume of 100 mL equipped with a PTFE stopper and sleeve, electrodes (interelectrode gap: 13 mm), electrode holders and a cross-shaped stirring bar. The cell is commercially available via *HWS Labortechnik* (Mainz, Germany). The electrolysis was conducted using a TDK-Lambda Z+ series (*TDK-Lambda UK Limited*, Devon, United Kingdom) as power source. The Sigraflex™ anode was held in place using a PTFE support frame. Its dimensions (excluding the area covered by the frame) were 6 cm × 2 cm × 0.02 cm and was fully submerged into the solution. This resulted in an active electrode area of 12 cm^2. As cathode, a stainless-steel wire (l = 7 cm, d = 0.1 cm) was used and fixated in front of the middle of the anode by a PTFE support frame. The stoppers and electrode holders are available through Sigma-Aldrich within the SynLectro™ series.

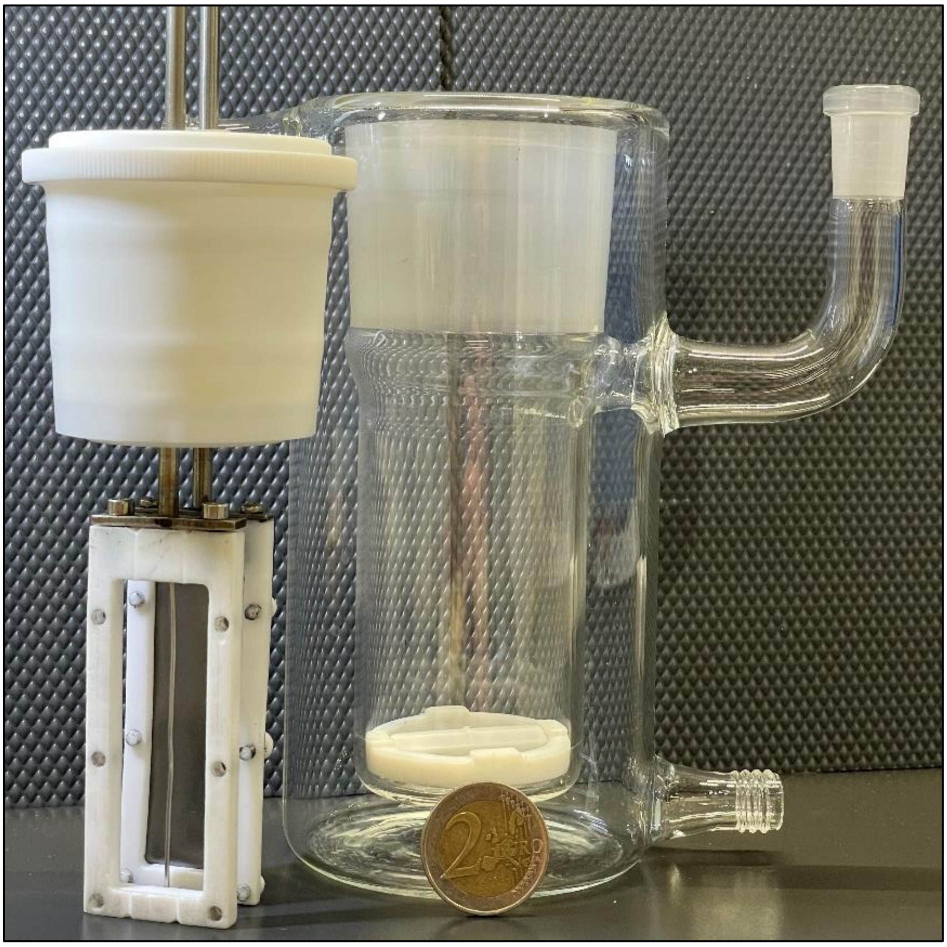

Figure S2: 100-mL quasi-divided glass cell with Sigraflex™ anode and stainless-steel wire cathode, electrode holders and stirring bar used for the scale-up experiment. Diameter of the coin 25.75 mm.

2 Experimental Procedures

2.1 Preparation of the SO_2 stock solution

In a gas-inlet apparatus connected to a washing bottle filled with aq. NaOH (20% v/v) with Woulff bottles placed before and after the washing bottle, acetonitrile (350 mL, anhydrous) was enriched with SO_2 at 0 °C under constant stirring for 15 minutes. After usage, the gas-inlet apparatus was flushed with Argon and molecular sieve (3 Å) was added to the obtained solution. The stock solution was sealed by a PTFE septum and stored in a fridge at 7 °C.

2.2 Determination of the SO_2 concentration of the stock solution

The SO_2 molarity was determined according to the principles of the "Excess Iodine Method" described by Ferguson.[3] To a solution of I_2 (1.27 g, 5.00 mmol) and KI (2.20 g, 13.3 mmol) in H_2O (100 mL) was slowly added the freshly prepared SO_2 stock solution (1 mL). The solution was then back titrated with a freshly prepared solution of $Na_2S_2O_3$ x 5 H_2O (aq., 0.2 mol/L) as titrant. After full reduction of the iodine, the concentration of SO_2 (mostly 3–5 mol/L) was calculated according to the previously reduced iodine by aq. SO_2. The titration was conducted three successive times, and the molarity of the stock solution was determined by averaging the three measurements.

2.3 General protocol for the synthesis of alkyl enaminylsulfonates (GP1)

The reactions were carried out using the undivided Teflon™ cells with a pretreated Sigraflex™ anode and a stainless-steel wire as a cathode as described in the section before.

Electrolyte: An undivided Teflon™ cell was charged with an alcohol **2** (2.60 mmol, 5.2 eq.), 1,8-diaza-bicyclo[5.4.0]undec-7-ene (DBU, 685 mg, 672 µL, 4.50 mmol, 9 eq.), SO_2 stock solution (7.8 eq., 1–2 mL, depending on the concentration of SO_2 in the stock solution used) and diluted with acetonitrile (anhydrous) so that a total volume of 5 mL was achieved. The cell was additionally loaded with a tertiary alkyl amine substrate **1** (500 µmol, 1 eq., 0.1 M).

The lid including a prefixed Sigraflex™ anode and a stainless-steel wire cathode was attached to the cell, the amperage was set accordingly so that a current density of 67.5 mA/cm^2 was reached (108 mA with the setup described herein) and the amount of applied charge was set to 11.5 *F* (555 C using 500 µmol of starting material). The electrolysis was conducted at room temperature under constant stirring (400 rpm) for ca. 1 h 25 min. After completion of the electrolysis the reaction mixture was transferred to a beaker and the cell as well as the electrodes were rinsed with acetonitrile (3 x 2 mL). The combined solutions were filtered over a short layer of silica (ca. 2 cm) using acetonitrile and the solvent was removed under reduced pressure. The crude product (approx. 250 mg) was loaded onto Celite™ (diatomaceous earth) using dichloromethane and purified by automated flash column chromatography (cyclohexane/ethyl acetate) with an appropriate gradient. Detection of the product was achieved by monitoring the UV-trace at 254 nm.

2.4 General protocol for the optimization of reaction conditions (GP2)

The reactions were carried out using the undivided Teflon™ cells with a PTFE lid including the anode and a stainless-steel cathode as described in the section before.

Electrolyte: An undivided Teflon™ cell was charged with neopentyl alcohol (**2a**), an organic base, SO_2 stock solution (in acetonitrile, anhydrous) and diluted with acetonitrile (anhydrous) so that a total volume of 5 mL was achieved. The cell was additionally loaded with *N,N*-diisoproylamine (DIPEA, **1a**, 64.6 mg, 87 µL, 500 µmol, 1 eq., 0.1 M) as a substrate.

The lid including the electrodes was attached to the cell, the amperage and amount of applied charge were set to the desired values and the electrolysis was conducted at room temperature under constant stirring (400 rpm). After completion, 1,3,5-trimethoxybenzene (1,3,5-TMB, 50 mg) was added to the reaction mixture as an internal standard. The solution was stirred for 5 min at room temperature and an aliquot (ca. 0.5 mL) was taken for qNMR measurement (^{1}H NMR, 400 MHz). The yield was calculated using the two alkenyl-protons of product **3a** (Figure S3).

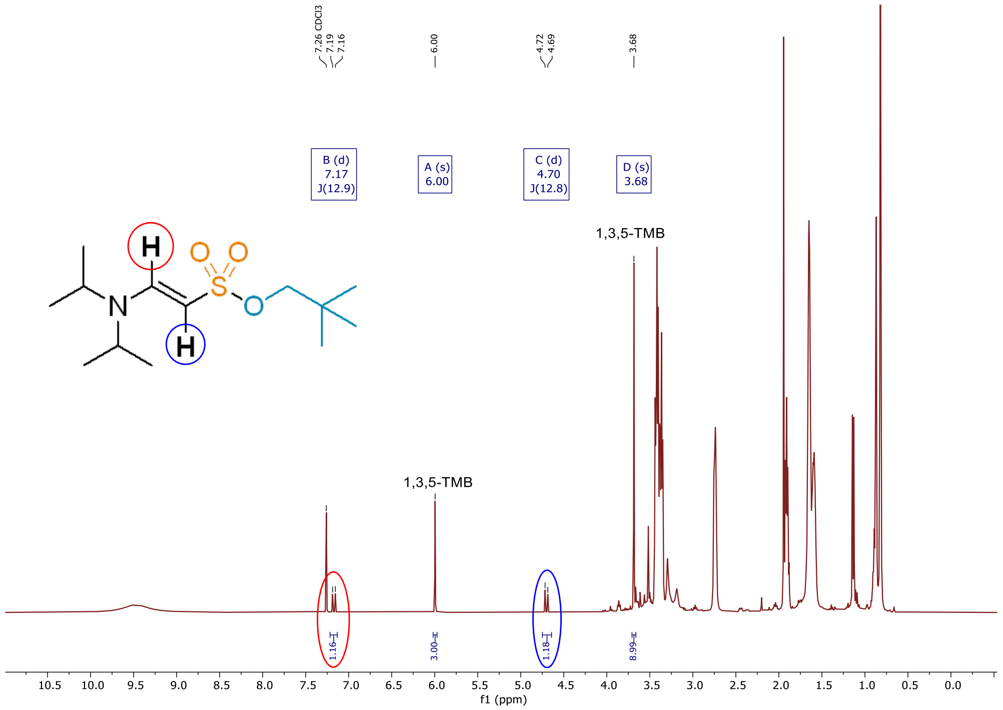

Figure S3: Typical 1H NMR spectrum of a reaction mixture used for quantification of product **3a**.

2.5 Protocol for the large-scale synthesis

The reactions was carried out using the undivided glass cell with a Sigraflex™ anode and a stainless-steel wire as a cathode as described in the section above.

Neopentyl (*E*)-2-(2,2,6,6-tetramethyl-piperidinyl)ethene sulfonate (3a)

Electrolyte: The glass cell was charged with *N*-ethyl-2,2,6,6-tetramethylpiperidine (**4a**, 1.69 g, 10.0 mmol, 1 eq.), neopentanol (**2a**, 4.58 g, 52.0 mmol, 5.2 eq.), 1,8-diaza-bicyclo[5.4.0]undec-7-ene (13.7 g, 13.4 mL, 90.0 mmol, 9 eq.), SO_2 stock solution (2.95 mol/L, 26.4 mL, 78.0 mmol of SO_2, 7.8 eq.) and diluted with acetonitrile (67.3 mL, anhydrous) so that a total volume of 100 mL was achieved.

The stopper including the electrodes and electrode holders was attached to the cell and connected to the power source. The amperage was set accordingly so that a current density of 67.5 mA/cm^2 was reached (810 mA with the setup described herein) and the amount of applied charge was set to 11.5 *F* (corresponds to 11096 C when using 10.0 mmol of starting material). The electrolysis was conducted at 23° C with active temperature control through the help of a cryostat and under constant stirring (700 rpm) for ca. 3 h 48 min. After completion of the electrolysis, the reaction mixture was transferred to a flask, the cell was thoroughly rinsed with acetonitrile and the solvent was removed under reduced pressure. The remaining mixture was filtered over a layer of silica (ca. 5 cm) using ethyl acetate (ca. 400 mL). The crude (ca. 10 g) was loaded onto Celite™ (diatomaceous earth) using dichloromethane and purified by automated flash column chromatography (cyclohexane/ethyl acetate = 98/2 → 70/30). The desired product neopentyl (*E*)-2-(2,2,6,6-tetramethyl-piperidinyl)ethene sulfonate (**3a**, 2.42 g, 7.62 mmol, 76%) was obtained as a colorless solid.

Spectroscopic data of the isolated compound matched the one obtained from the small-scale reaction described in section 6.4.

Note: During the reaction, extensive bubble formation was observed on the cathode. The gas is believed to be H_2 originating from the degradation of solvent or the reduction of protonated base. An ^{1}H NMR yield of 84% was calculated by addition of 1,3,5-trimethoxybenzene (508.6 mg) as internal standard to the reaction mixture upon removal from the cell. After filtration over silica, a second ^{1}H NMR sample was taken and a yield of 83% was calculated.

Neopentyl (E)-2-(N-methylacetamido)ethene sulfonate (3n)

Electrolyte: The glass cell was charged with *N*-methyl-*N*-vinylacetamide (991 mg, 1.03 mL, 10.0 mmol, 1 eq.), neopentanol (**2a**, 4.58 g, 52.0 mmol, 5.2 eq.), 1,8-diaza-bicyclo[5.4.0]undec-7-ene (13.7 g, 13.4 mL, 90.0 mmol, 9 eq.), SO_2 stock solution (4.15 mol/L, 18.8 mL, 78.0 mmol of SO_2, 7.8 eq.) and diluted with acetonitrile (75.6 mL, anhydrous) so that a total volume of 100 mL was achieved.

The stopper including the electrodes and electrode holders was attached to the cell and connected to the power source. The amperage was set accordingly so that a current density of 67.5 mA/cm^2 was reached (810 mA with the setup described herein) and the amount of applied charge was set to 5.75 *F* (corresponds to 5548 C when using 10.0 mmol of starting material). The electrolysis was conducted at 23° C with active temperature control through the help of a cryostat and under constant stirring (700 rpm) for ca. 1 h 54 min. After completion of the electrolysis, the reaction mixture was transferred to a flask, the cell was thoroughly rinsed with acetonitrile and the solvent was removed under reduced pressure. The remaining mixture was filtered over a layer of silica (ca. 5 cm)

using ethyl acetate (ca. 400 mL). The crude (ca. 10 g) was loaded onto Celite™ (diatomaceous earth) using dichloromethane and purified by automated flash column chromatography (cyclohexane/ethyl acetate = 96/4 → 60/40). The desired product neopentyl (*E*)-2-(*N*-methylacetamido)ethene sulfonate (**3n**, 2.08 g, 8.35 mmol, 84 %) was obtained as a colorless solid.

Spectroscopic data of the isolated compound matched the one obtained from the small-scale reaction described in section 6.14.

Note: During the reaction, extensive bubble formation was observed on the cathode. The gas is believed to be H_2 originating from the degradation of solvent or the reduction of protonated base. An ^{1}H NMR yield of 51% was calculated by addition of 1,3,5-trimethoxybenzene (456.5 mg) as internal standard to the reaction mixture upon removal from the cell. After filtration over silica, a second ^{1}H NMR sample was taken and a yield of 59% was calculated. The difference in NMR- to isolated yield is probably due to insufficient relaxation time during 1H NMR measurement.

3 Optimization

Optimization of the conditions was carried out by using linear screening for the discrete and design-of-experiment-studies (DoE) for continuous parameters. *N,N*-diisoproylamine (**1a**, DIPEA) and neopentyl alcohol (**2a**) were used as substrates according to the procedure described in GP2. Neopentyl alcohol was chosen due to the enhanced stability of the respective sulfonate ester.[4] Yields were determined by ^{1}H NMR spectroscopy *via* the addition of 1,3,5-trimethoxybenzene (50 mg) as an internal standard after the end of electrolysis.

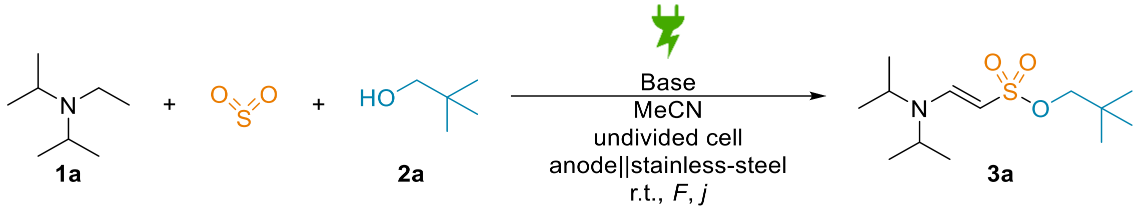

Initial experiments were carried out in an undivided Teflon™ cell with a planar graphite anode and a planar stainless-steel cathode (Table S2, Entry 1 & 2), with 1,8-diaza-bicyclo[5.4.0]undec-7-ene (DBU) as a supporting base. When the geometry of the cathode was changed to a thin wire (d = 0.1 cm), yield improved significantly (Entry 3).

Table S2: First experiments.

Entry	Neopentanol [eq.]	SO_2 [eq.]	DBU [eq.]	Cathode	Yield (^{1}H NMR)
1	2.0	3.0	5.0	Stainless-steel plate	24%
2	4.0	6.0	8.0	Stainless-steel plate	41%
3	4.0	6.0	8.0	Stainless-steel wire	50%

Conditions (GP2): *N,N*-diisopropylamine (500 µmol, 1 eq., 0.1 M), SO_2 stock solution (3–6 eq.), neopentyl alcohol (2–4 eq.), DBU (5–8 eq.), MeCN, graphite||stainless-steel, 40 mA/cm^2, 10 *F*, r.t., 400 rpm.

Next, several carbon-based anode materials were tested (Table S3). Sigraflex™ (Entry 4), an inexpensive graphite foil, gave the best results. Supposedly, this is due do the materials improved porosity, compared to a standard isostatic graphite electrode. Screening was continued with this material.

Table S3: Screening of anode materials.

Entry	Anode material	Yield (^{1}H NMR)
1	Graphite	50%
2	Glassy Carbon	16%
3	Boron-doped diamond	18%
4	Sigraflex™	55%

Conditions (GP2): *N,N*-diisopropylamine (500 µmol, 1 eq., 0.1 M), SO_2 stock solution (6 eq.), neopentyl alcohol (4 eq.), DBU (8 eq.), MeCN, anode||stainless-steel wire, 40 mA/cm^2, 10 *F*, r.t., 400 rpm.

Screening of different bases (Table S4) showed no improvement to the previously used conditions, so the optimization was continued with 1,8-diaza-bicyclo[5.4.0]undec-7-ene (DBU, Entry 1).

Table S4: Screening of bases.

Entry	Base	Yield (^{1}H NMR)
1	1,8-Diaza-bicyclo[5.4.0]undec-7-ene (DBU)	55%
2	1,5-Diazabicyclo(4.3.0)non-5-ene (DBN)	51%
3	1,1,3,3-Tetramethylguanidine (TMG)	49%
4	2-*tert*-Butyl-1,1,3,3-tetramethylguanidine (^{t}BuTMG, Bartons Base)	55%
5	2,4,6-Collidine	0%
6	2,6-Lutidine	0%
7	7-Methyl-1,5,7-triazabicyclo[4.4.0]dec-5-en (MeTBD)	49%

Conditions (GP2): *N,N*-diisopropylamine (500 µmol, 1 eq., 0.1 M), SO_2 stock solution (6 eq.), neopentyl alcohol (4 eq.), base (8 eq.), MeCN, Sigraflex™||stainless-steel wire, 40 mA/cm^2, 10 *F*, r.t., 400 rpm.

To improve the yield further, a design-of-experiment study was conducted (2^{4-1}-design plan, duplicates for each experiment, triplicate for the center point (CP), randomized order of experiments). The following parameters were investigated in their respective ranges:

- Amount of neopentyl alcohol: 2–5 eq.
- Amount of base: 5–10 eq.
- Current density *j*: 25–75 mA/cm^2.
- Amount of applied charge *Q*: 5–10 *F*.

To maintain an excess of SO_2, its amount was adjusted according to the stoichiometric equivalents of neopentyl alcohol, with the molar ratio of SO_2 to neopentyl alcohol set at 1.5:1 (eq. [SO_2] = 1.5 × eq. [ROH]). Since initial data evaluation revealed curvature of the model, additional star point (SP) experiments were added. The experimental results are summarized in Table S5.

Table S5: Design-of-experiment study.

Entry	Type	Neopentyl alcohol [eq.]	DBU [eq.]	Q [*F*]	*j* [mA/cm^2]	Yield (^{1}H NMR)
1	-	5.0	10	10	75	69%
2	CP	3.5	7.5	7.5	50	56%
3	-	5	5	5	75	38%
4	-	2	10	5	75	28%
5	-	2	5	5	25	38%
6	-	2	5	5	25	36%
7	-	2	10	5	75	29%
8	-	2	10	5	25	37%
9	-	2	5	10	75	53%
10	-	2	10	10	25	23%
11	CP	3.5	7.5	7.5	50	59%
12	-	5	5	10	25	44%
13	-	2	5	10	25	44%
14	-	5	5	10	25	51%
15	-	2	10	10	25	22%
16	CP	3.5	7.5	7.5	50	58%
17	-	5	5	5	75	39%
18	-	5	10	5	25	37%
19	-	5	10	10	75	60%
20	SP	1.1	7.5	7.5	50	23%
21	SP	5.9	7.5	7.5	50	46%
22	SP	3.5	3.5	7.5	50	51%
23	SP	3.5	11.5	7.5	50	37%
24	SP	3.5	7.5	3.5	50	35%
25	SP	3.5	7.5	11.5	50	56%
26	SP	3.5	7.5	7.5	10	34%
27	SP	3.5	7.5	7.5	90	53%

Conditions (GP2): *N,N*-diisopropylamine (500 µmol, 1 eq., 0.1 M), SO_2 stock solution (1.5 x eq. [neopentyl alcohol]), neopentyl alcohol (1.1–5.9 eq.), DBU (3.5–11.5 eq.), MeCN, Sigraflex™||stainless-steel wire, 10–90 mA/cm^2, 3.5–11.5 *F*, r.t., 400 rpm.

The following analyses were conducted, demonstrating adequate statistical significance to support the validity of the DoE:

Table S6: Statistical evaluation of the DoE study.

Source	DF	Adj SS	Adj MS	F-Value	P-Value
Model	12	4005.63	333.803	31.91	0.000
Blocks	1	119.13	119.133	11.39	0.005
Linear	4	2236.23	559.057	53.45	0.000
ROH [eq.]	1	912.19	912.189	87.21	0.000

DBU [eq.]	1	172.73	172.735	16.51	0.001
Q [F]	1	654.82	654.818	62.60	0.000
j [mA/cm^2]	1	496.48	496.485	47.47	0.000
Square	4	893.87	223.467	21.36	0.000
ROH [eq.] * ROH [eq.]	1	627.69	627.685	60.01	0.000
DBU [eq.] * DBU [eq.]	1	146.20	146.200	13.98	0.002
Q [F] * *Q* [F]	1	100.90	100.895	9.65	0.008
j [mA/cm^2] * *j* [mA/cm^2]	1	163.16	163.164	15.60	0.001
2-Way Interactions	3	865.50	288.500	27.58	0.000
ROH [eq.] * DBU [eq.]	1	625.00	625.000	59.75	0.000
ROH [eq.] * *Q* [F]	1	240.25	240.250	22.97	0.000
ROH [eq.] * *j* [mA/cm^2]	1	0.25	0.250	0.02	0.879
Error	14	146.44	10.460		
Lack-of-Fit	4	32.77	8.193	0.72	0.597
Pure Error	10	113.67	11.367		

Table S7: Model summary.

S	R^2	R^2 (adj)	R^2 (pred)
3.23419	96.47%	93.45%	86.62%

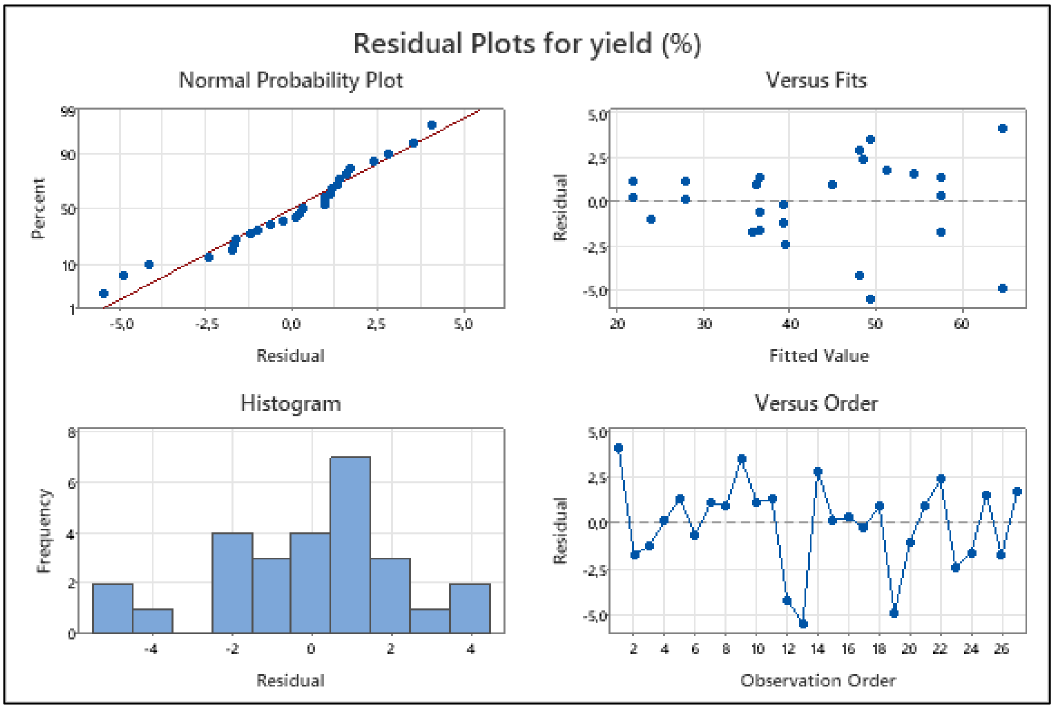

Figure S4: Residual plots.

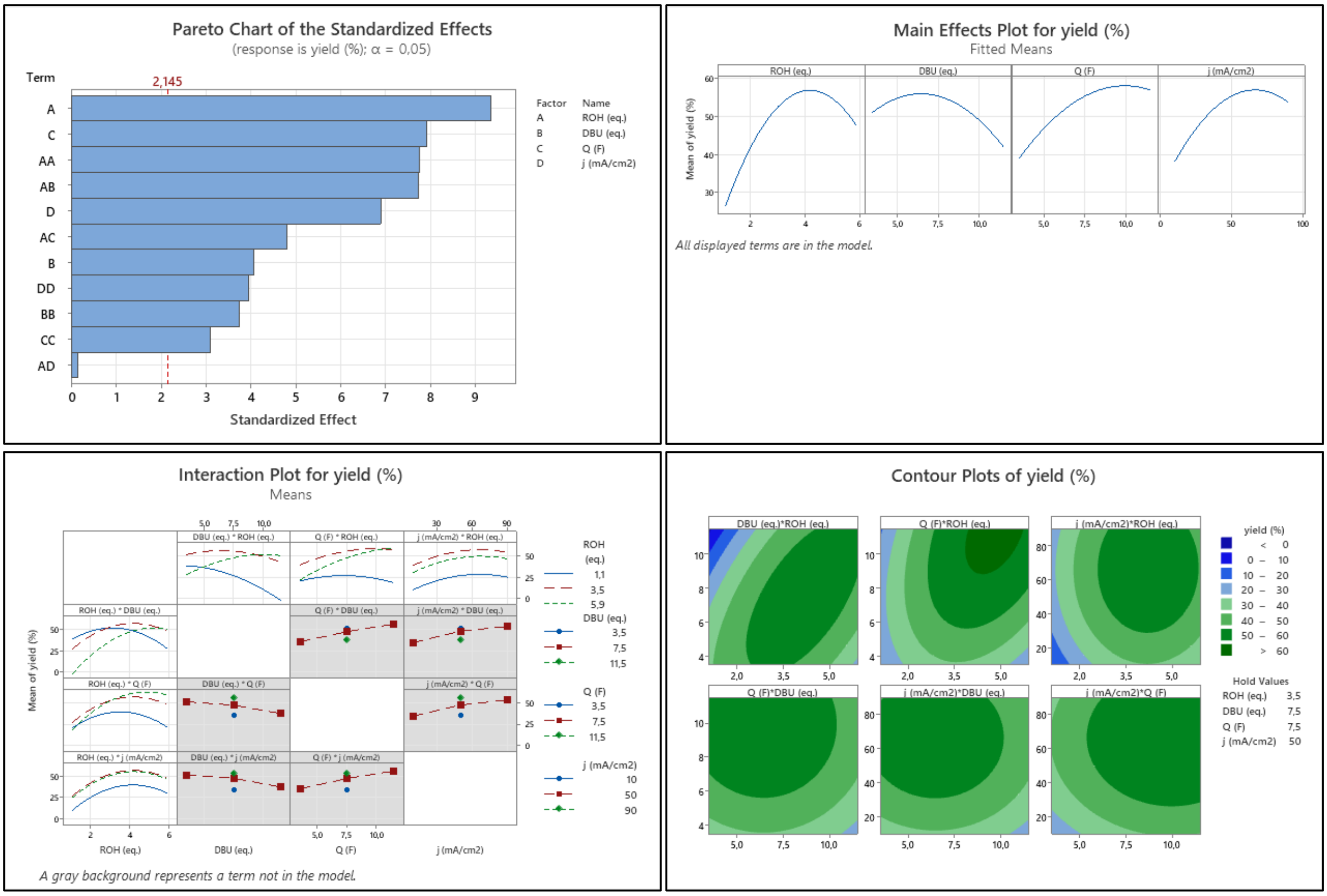

Figure S5: Analysis of main effects and interactions.

Response optimization analysis (Figure S6) determined the following optimal parameter values, with a predicted yield of 65%:

- Amount of neopentyl alcohol: 5.2 eq.
- Amount of base: 9.0 eq.
- Current density *j*: 67.5 mA/cm^2
- Amount of applied charge *Q*: 11.5 *F*

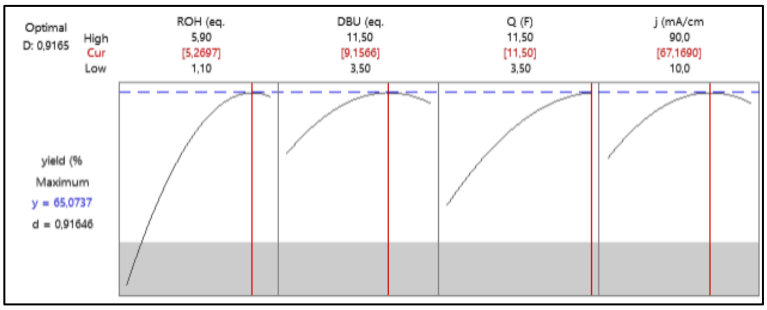

Figure S6: Results of the response optimization.

This prediction was verified in an experiment using the calculated parameter settings (Table S8, Entry 1). Yield was further improved to 70% by pretreating the Sigraflex™ anode in acetonitrile for two hours prior to use (Entry 2). This caused some minor swelling, which is believed to improve diffusion of reactants into the porous electrode. The conditions of Table S8, Entry 2 were regarded as optimal and, if not stated otherwise, used in all following experiments.

Table S8: Final reaction conditions.

Entry	Neopentyl alcohol [eq.]	SO2 [eq.]	DBU [eq.]	j [mA/cm2]	Q [*F*]	Yield (1H NMR)
1	5.2	7.8	9.0	67.5	11.5	65%
2	5.2	7.8	9.0	67.5	11.5	70%[a]

Conditions (GP2): *N*,*N*-diisopropylamine (500 µmol, 1 eq., 0.1 M), SO_2 stock solution (7.8 eq.), neopentyl alcohol (5.2 eq.), DBU (9.0 eq.), MeCN, Sigraflex™||stainless-steel wire, 67.5 mA/cm^2, 11.5 *F*, r.t., 400 rpm. [a]Pretreating of Sigraflex™ electrode in acetonitrile 2 h before usage.

4 Control Experiments

To gain insight into the reaction, some control experiments (Table S9) were performed using the optimized conditions from Table S8, Entry 2:

Table S9: Control experiments.

Entry	Deviation from the standard conditions[a]	Yield (^{1}H NMR)
1	No electricity; stirred for 1.5 h at room temperature	0%
2	No DBU (0.1 M Bu_4NBF_4 added for conductivity)	0%
3	+ (2,2,6,6-Tetramethylpiperidinyl)oxyl (TEMPO, 3 eq.)	38%
4	+ 2,6-Di-tert-butyl-4-methylphenol (BHT, 3 eq.)	20%

[a]**Conditions (GP2):** *N,N*-diisopropylamine (500 µmol, 1 eq., 0.1 M), SO_2 stock solution (7.8 eq.), neopentyl alcohol (5.2 eq.), DBU (9.0 eq.), MeCN, Sigraflex™||stainless-steel wire, 67.5 mA/cm^2, 11.5 *F*, r.t., 400 rpm.

In the case of BHT (Table S9, Entry 4), we were able to detect two trapped products *via* GC/MS (EI). They result from the addition of a DIPEA-derived enamine (Figure S7 top) or neopentyl-alcohol-derived sulfinate (Figure S7 bottom) to oxidized BHT, respectively:

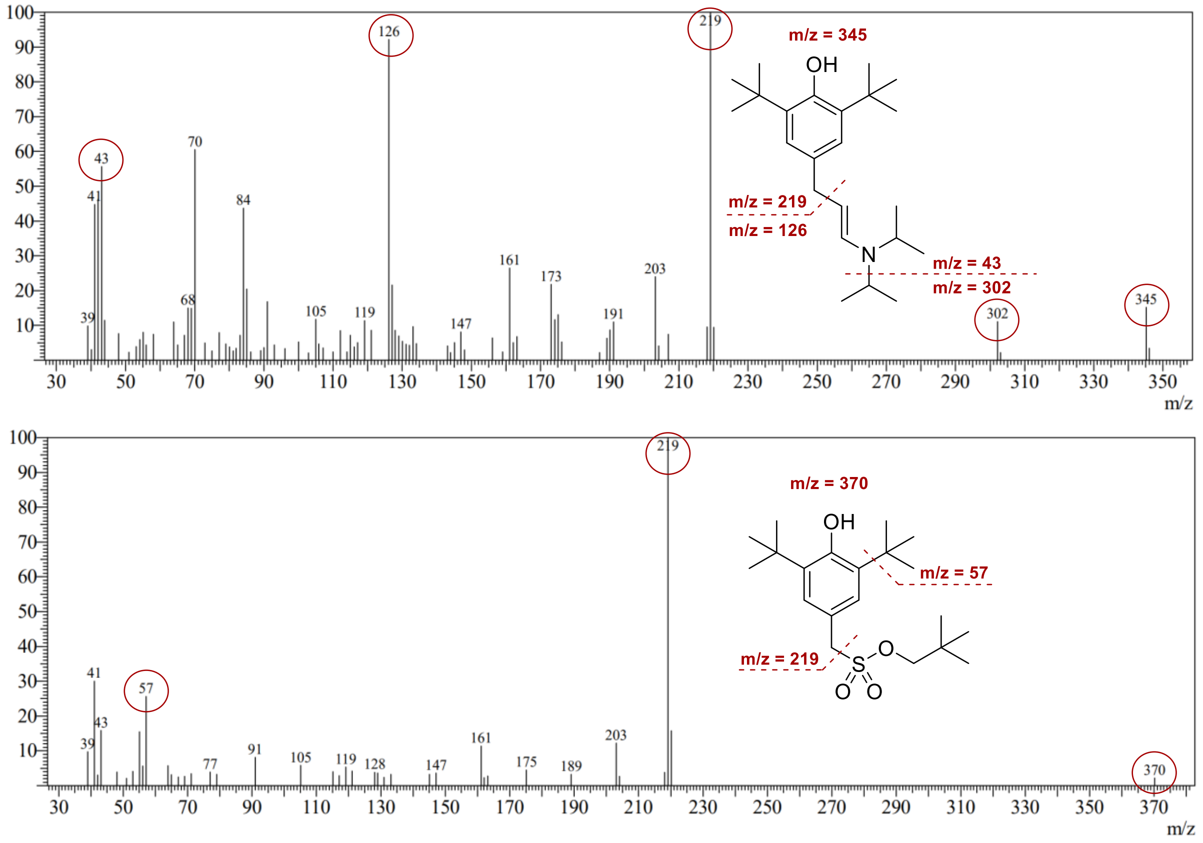

Figure S7: Trapped intermediates using BHT detected in GC/MS (EI).

5 Cyclovoltammetry Studies

For the elucidation of the mechanism cyclic voltammetry was performed (Figure S8). The redox behavior of the starting material DIPEA (**1a**) shows an oxidation wave at 0.66 V vs. FcH/FcH$^+$ (red trace). Oxidation of the enaminyl sulfonate **3a** is shifted towards a more positive potential (1.25 V vs. FcH/FcH$^+$, green trace), therefore overoxidation is prevented. The in-situ generated monoalkylsulfite intermediate **D** (blue trace) shows an oxidation peak at 2.09 V and a second oxidation/ degradation wave at 2.94 V. This clearly shows that during electrolysis, the amine substrate **1a** is initially oxidized, as evidenced by literature reports as well.[5]

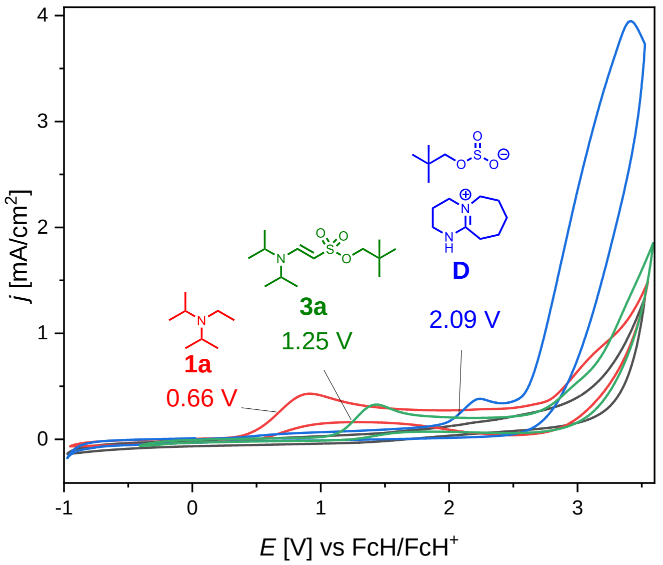

Figure S8: Cyclic voltammogram of *N,N*-diisopropylethylamine (**1a**, red), the corresponding enaminyl sulfonate product **3a** (green), the intermediate monoalkylsulfite **D** (blue) and a blank measurement (grey). Measurement conditions: 0.1 M Bu_4NBF_4 in MeCN; c(substrate) = 10 mmol/L; c(SO_2) = 40 mmol/L; v = 100 mV/s. WE: Glassy Carbon, RE: Ag/$AgNO_3$, CE: Glassy Carbon rod. All measurements were performed at room temperature. Graphic displayed according to IUPAC convention.

The redox potentials regarding the substrate 1-ethyl-2,2,6,6-tetramethylpiperidine (**1d,** Figure S9) shows a similar behavior, where the starting material oxidizes at 0.66 V vs. FcH/FcH$^+$ (red trace) and at a lower potential than the corresponding enaminyl sulfonate (**3d**, green) at 1.29 V and the in-situ generated monoalkylsulfite intermediate **D** (blue trace) at 2.09 V and 2.94 V, respectively.

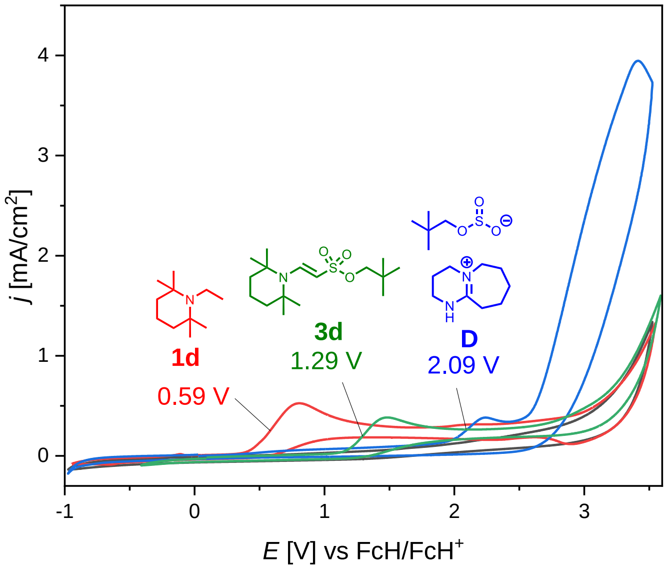

Figure S9: Cyclic voltammogram of 1-ethyl-2,2,6,6-tetramethylpiperidine (**1d**, red), the corresponding enaminyl sulfonate product **3d** (green), the intermediate monoalkylsulfite **D** (blue) and a blank measurement (grey). Measurement conditions: 0.1 M Bu_4NBF_4 in MeCN; c(substrate) = 10 mmol/L; c(SO_2) = 40 mmol/L; v = 100 mV/s. WE: Glassy Carbon, RE: Ag/$AgNO_3$, CE: Glassy Carbon rod. All measurements were performed at room temperature. Graphic displayed according to IUPAC convention.

When a similar intermediate was formed with morpholine instead of neopentyl alcohol,[6] a gradual oxidation wave starting at ca. 0.93 V was observed (Figure S10). This peak is within the oxidation range of the aforementioned DIPEA. This possible competing oxidation reaction offers an additional explanation for the lower yields observed for the formation of the sulfonamides **5a** and **5b**.

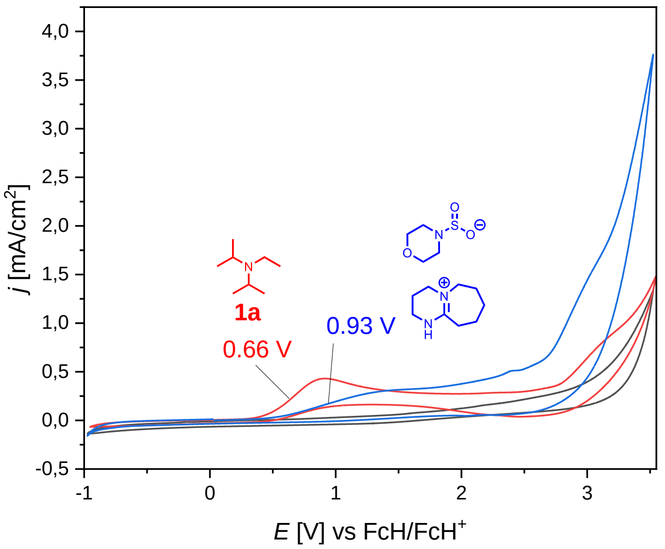

Figure S10: Cyclic voltammogram of *N,N*-diisopropylethylamine (**1a**, red) and a possible intermediate amidosulfinate (blue). Measurement conditions: 0.1 M Bu_4NBF_4 in MeCN; c(substrate) = 10 mmol/L; c(SO_2) = 40 mmol/L; v = 100 mV/s. WE: Glassy Carbon, RE: Ag/AgNO₃, CE: Glassy Carbon rod. All measurements were performed at room temperature. Graphic displayed according to IUPAC convention.

6 Compound Characterization

6.1 Neopentyl (*E*)-2-(diisopropylamino)ethene sulfonate (3a)

According to GP1, *N*,*N*-diisopropylethylamine (64.4 mg, 87 µL, 500 µmol, 1 eq.), SO_2 stock solution (3.90 mmol, 7.8 eq.) and neopentyl alcohol (229 mg, 2.60 mmol, 5.2 eq.) were used as substrates and the electrolysis was carried out using 11.5 *F*. After purification by flash column chromatography (cyclohexane/ethyl acetate = 98/2 → 82/18), the desired product (84.3 mg, 304 µmol, 61%) was obtained as a colorless solid.

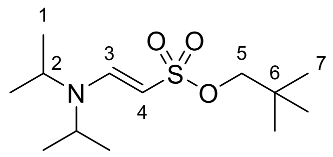

^{1}H NMR (500 MHz, CD_3CN, 25 °C) δ = 7.22 (d, *J* = 12.8 Hz, 1H, 3-*H*), 4.86 (d, *J* = 12.8 Hz, 1H, 4-*H*), 3.69 (brs, 2H, 2-*H*), 3.57 (s, 2H, 5-*H*), 1.18 (d, *J* = 6.7 Hz, 12H, 1-*H*), 0.93 (s, 9H, 7-*H*) ppm.
^{1}H NMR (500 MHz, CD_3CN, -30 °C) δ = 7.18 (d, *J* = 12.8 Hz, 1H, 3-*H*), 4.84 (d, *J* = 12.8 Hz, 1H, 4-*H*), 3.72 (hept, *J* = 6.7 Hz, 1H, 2-*H*), 3.62 (hept, *J* = 6.7 Hz, 1H, 2'-*H*), 1.15 (dd, *J*= 8.5, 6.7 Hz, 12H, 1-*H*), 0.89 (s, 9H, 7-*H*) ppm.
^{13}C NMR (126 MHz, CD_3CN) δ = 147.6, 84.8, 78.2, 50.1, 48.8, 32.1, 26.4, 23.4, 19.4 ppm.
HRMS (EI): *m/z* for $C_{13}H_{27}NO_3S^+$ [M]$^+$: calc.: 277.1706, found: 277.1711.

Note: Hindered rotation around the N-3-bond caused broadened peaks in the ^{1}H NMR spectrum (3.69 ppm) and doublet peaks in the ^{13}C NMR spectrum (50.1 & 48.8 ppm, 26.4 & 23.4 ppm) measured at 25 °C. This could be resolved by lowering the measurement temperature to -30 °C.

6.2 Neopentyl (*E*)-2-(cyclohexyl(ethyl)amino)ethene sulfonate (3b)

According to GP1, *N,N*-diethylcyclohexylamine (77.6 mg, 92 µL, 500 µmol, 1 eq.), SO_2 stock solution (3.90 mmol, 7.8 eq.) and neopentyl alcohol (229 mg, 2.60 mmol, 5.2 eq.) were used as substrates and the electrolysis was carried out using 11.5 *F*. After purification by flash column chromatography (cyclohexane/ethyl acetate = 98/2 → 82/18), the desired product (84.4 mg, 278 µmol, 56%) was obtained as a colorless solid.

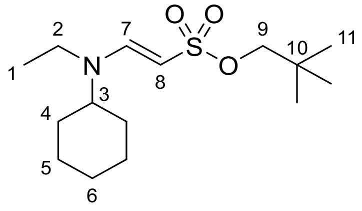

^{1}H NMR (500 MHz, $CDCl_3$) δ = 7.26 (d, J = 12.8 Hz, 1H, 7-*H*), 4.71 (d, J = 12.7 Hz, 1H, 8-*H*), 3.62 (s, 2H, 9-*H*), 3.14 (q, J = 7.4 Hz, 2H, 2-*H*), 3.03 (brd, J = 14.5 Hz, 1H, 3-*H*), 1.87 – 1.81 (m, 4H, CyHex-*H*), 1.70 – 1.64 (m, 1H, CyHex-*H*), 1.41 (qd, J = 12.7, 3.7 Hz, 2H, CyHex-*H*), 1.33 – 1.24 (m, 3H, CyHex-*H*), 1.16 – 1.11 (m, 3H, 1-*H*), 0.96 (s, 9H, 11-*H*) ppm.
^{13}C NMR (126 MHz, $CDCl_3$) δ = 148.7, 84.1, 77.7, 65.2, 60.7, 56.9, 41.9, 39.7, 32.6, 31.7, 29.9, 29.3, 26.4, 26.2, 26.1, 25.9, 25.3, 24.9, 24.7, 12.2, 11.5 ppm.
HRMS (EI): *m/z* for $C_{15}H_{29}NO_3S^+$ [M]$^+$: calc.: 303.1863, found: 303.1861.

Note: Hindered rotation around the N-7 bond caused doublet peaks for some of the carbon atoms in the ^{13}C NMR spectrum.

6.3 Neopentyl (*E*)-2-(dicyclohexylamino)ethene sulfonate (3c)

According to GP1, *N*,*N*-dicyclohexylethylamine (105 mg, 115 µL, 500 µmol, 1 eq.), SO_2 stock solution (3.90 mmol, 7.8 eq.) and neopentyl alcohol (229 mg, 2.60 mmol, 5.2 eq.) were used as substrates and the electrolysis was carried out using 11.5 *F*. After purification by flash column chromatography (cyclohexane/ethyl acetate = 98/2 → 84/16), the desired product (90.6 mg, 253 µmol, 51%) was obtained as a colorless solid.

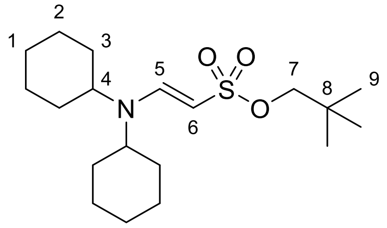

^{1}H NMR (500 MHz, CDCl$_3$) δ = 7.27 (d, *J* = 12.7 Hz, 1H, 5-*H*), 4.78 (d, *J* = 12.7 Hz, 1H, 6-*H*), 3.61 (s, 2H, 7-*H),* 3.23 – 3.01 (brm, 2H, 4-*H*), 1.83 (d, *J* = 12.0 Hz, 5H, *CyHex-H*), 1.77 – 1.55 (m, 3H, *CyHex-H*), 1.44 (qd, *J* = 12.4, 3.4 Hz, 4H, *CyHex-H*), 1.37 – 1.19 (m, 6H, *CyHex-H*), 1.12 (qt, *J* = 11.6, 2.9 Hz, 2H, *CyHex-H*), 0.96 (s, 9H, 9-*H*) ppm.
^{13}C NMR (126 MHz, CDCl$_3$) δ = 147.2, 84.0, 77.6, 58.5, 56.6, 54.1, 34.8, 34.3, 31.7, 30.5, 30.0, 29.4, 26.4, 26.0, 25.4, 24.9, 24.9 ppm.
HRMS (EI): *m/z* for $C_{19}H_{35}NO_3S^+$ [M]$^+$: calc.: 357.2332, found: 357.2334.

Note: Due to hindered rotation around the N-5-bond a full set of peaks was observed for the two cyclohexyl groups in the ^{13}C NMR spectrum.

6.4 Neopentyl (*E*)-2-(2,2,6,6-tetramethylpiperidinyl)ethene sulfonate (3d)

According to GP1, 1-ethyl-2,2,6,6-tetramethylpiperidine (84.7 mg, 500 µmol, 1 eq.), SO_2 stock solution (3.90 mmol, 7.8 eq.) and neopentyl alcohol (229 mg, 2.60 mmol, 5.2 eq.) were used as substrates and the electrolysis was carried out using 11.5 *F*. After purification by flash column chromatography (cyclohexane/ethyl acetate = 98/2 → 80/20), the desired product (135 mg, 423 µmol, 85%) was obtained as a colorless solid.

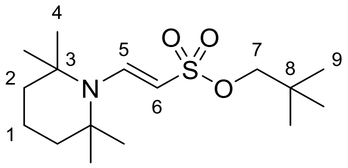

^{1}H NMR (500 MHz, CDCl$_3$) δ = 7.45 (d, *J* = 13.8 Hz, 1H, 5-*H*), 5.03 (d, *J* = 13.8 Hz, 1H, 6-*H*), 3.60 (s, 2H, 7-*H*), 1.67 – 1.61 (m, 6H, 1-*H*, 2-*H),* 1.36 (s, 12H, 4-*H),* 0.95 (s, 9H, 9-*H*) ppm.
^{13}C NMR (126 MHz, CDCl$_3$) δ = 146.7, 91.2, 77.8, 57.8, 41.1, 31.6, 28.8, 26.4, 16.4 ppm.
HRMS (EI): *m/z* for $C_{16}H_{31}NO_3S^+$ [M]$^+$: calc.: 317.2019, found: 317.2019.

6.5 Neopentyl (*E*)-2-(diethylamino)ethene sulfonate (3e)

According to GP1, triethylamine (50.6 mg, 69 µL, 500 µmol, 1 eq.), SO_2 stock solution (3.90 mmol, 7.8 eq.) and neopentyl alcohol (229 mg, 2.60 mmol, 5.2 eq.) were used as substrates and the electrolysis was carried out using 11.5 *F*. After purification by flash column chromatography (cyclohexane/ethyl acetate = 98/2 → 71/29), the desired product (89.2 mg, 358 µmol, 72%) was obtained as a colorless oil.

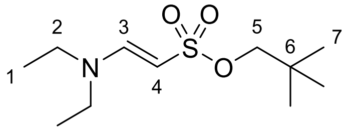

^{1}H NMR (500 MHz, CDCl$_3$) δ = 7.18 (d, *J* = 12.8 Hz, 1H, 3-*H*), 4.69 (d, *J* = 12.7 Hz, 1H, 4-*H*), 3.60 (s, 2H, 5-*H*), 3.29 – 3.05 (brm, 4H, 2-*H*), 1.22 – 1.10 (brm, 6H, 1-*H*), 0.94 (s, 9H, 7-*H*) ppm.
^{13}C NMR (126 MHz, CDCl$_3$) δ = 150.2, 84.4, 77.8, 50.2, 42.7, 42.3, 31.6, 26.3, 14.8, 11.2, 11.1 ppm.
HRMS (EI): *m/z* for $C_{11}H_{23}NO_3S^+$ [M]$^+$: calc.: 249.1393, found: 249.1395.

Note: Hindered rotation around the N-3 bond caused broadened signals in the ^{1}H NMR and a full set of peaks in the ^{13}C NMR spectrum.

6.6 Neopentyl (*E*)-1-(dipropylamino)prop-1-ene-2-sulfonate (3f)

According to GP1, tripropylamine (71.6 mg, 96 µL, 500 µmol, 1 eq.), SO_2 stock solution (3.90 mmol, 7.8 eq.) and neopentyl alcohol (229 mg, 2.60 mmol, 5.2 eq.) were used as substrates and the electrolysis was carried out using 11.5 *F*. After purification by flash column chromatography (cyclohexane/ethyl acetate = 98/2 → 83/17), the desired product (21.4 mg, 73.4 µmol, 15%) was obtained as a colorless oil.

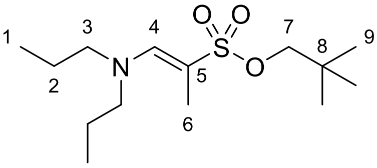

^{1}H NMR (500 MHz, CDCl$_3$) δ = 7.07 (s, 1H, 4-*H*), 3.52 (s, 2H, 7-*H*), 3.23 – 3.05 (m, 4H, 3-*H*), 2.01 (d, *J* = 0.8 Hz, 3H, 6-*H*), 1.59 (tq, *J* = 7.4, 7.4 Hz, 4H, 2-*H*), 0.96 (s, 9H, 9-*H*), 0.90 (t, *J* = 7.4 Hz, 6H, 1-*H*) ppm.
^{13}C NMR (126 MHz, CDCl$_3$) δ = 146.7, 91.9, 77.8, 54.8, 31.6, 26.5, 22.7, 11.3, 11.0 ppm.
HRMS (EI): *m/z* for $C_{14}H_{29}NO_3S^+$ [M]$^+$: calc.: 291.1863, found: 291.1864.

Note: $^4J_{H,H}$ coupling constant between 4-H and 6-H was determined to be 0.8 Hz. No $^1H,^1H$-NOESY-contact was found between these two protons. $^1H,^{13}C$-HMBC showed a $^3J_{C,H}$ coupling constant of 8.0 Hz between 4-H and carbon #6. Therefore, the product was determined to be (E)-configurated. Numerous byproducts originating from the dealkylation and/or oxidation of the amine substrate were detected via GC/MS (e.g. dipropylamine or N,N-dipropylformamide), offering an explanation for the reduced yield.

6.7 Neopentyl (*E*)-1-(dibutylamino)but-1-ene-2-sulfonate (3g)

According to GP1, tributylamine (92.7 mg, 119 µL, 500 µmol, 1 eq.), SO_2 stock solution (3.90 mmol, 7.8 eq.) and neopentyl alcohol (229 mg, 2.60 mmol, 5.2 eq.) were used as substrates and the electrolysis was carried out using 11.5 *F*. After purification by flash column chromatography (cyclohexane/ethyl acetate = 99/1 → 87/13), the desired product (17.0 mg, 51.0 µmol, 10%) was obtained as a yellowish oil.

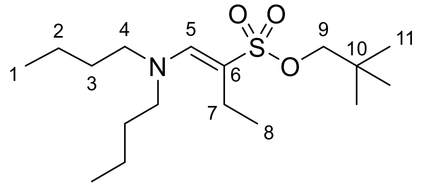

^{1}H NMR (500 MHz, CDCl$_3$) δ = 7.05 (s, 1H, 5-*H*), 3.54 (s, 2H, 9-*H*), 3.19 – 3.12 (m, 4H, 4-*H*), 2.38 (q, J = 7.4 Hz, 2H, 7-*H*), 1.58 – 1.50 (m, 4H, 3-*H*), 1.35 – 1.26 (m, 4H, 2-*H*), 1.16 (t, J = 7.4 Hz, 3H, 8-*H*), 0.95 (s, 9H, 11-*H*), 0.94 (t, J = 7.4 Hz, 6H, 1-*H*) ppm.
^{13}C NMR (126 MHz, CDCl$_3$) δ = 146.2, 99.0, 77.5, 52.8, 31.6, 31.3, 26.5, 19.9, 19.0, 16.0, 14.0 ppm.
HRMS (ESI+): *m/z* for $C_{17}H_{35}NO_3SNa^+$ [M+Na]$^+$: calc.: 356.2330, found: 356.2233.

Note: No $^1H,^1H$-NOESY-contact was found between 5-H and 7-H or 8-H. 7-H showed a NOESY-contact to 4-H. There-fore, the product was determined to be E-configured. Numerous byproducts originating from the dealkylation and/or oxidation of the amine substrate were detected via GC/MS (e.g. dibutylamine or N,N-dibutylformamide), offering an explanation for the reduced yield.

6.8 Neopentyl (*E*)-2-(piperidinyl)ethene sulfonate (3h)

According to GP1, *N*-Ethylpiperidine (56.6 mg, 69 µL, 500 µmol, 1 eq.), SO_2 stock solution (3.90 mmol, 7.8 eq.) and neopentyl alcohol (229 mg, 2.60 mmol, 5.2 eq.) were used as substrates and the electrolysis was carried out using 11.5 *F*. After purification by flash column chromatography (cyclohexane/ethyl acetate = 97/3 → 75/25), a product mixture of the *exo*- and the *endo*-product was obtained in the same fraction (27.0 mg, 103 µmol, 21%) as a colorless solid.

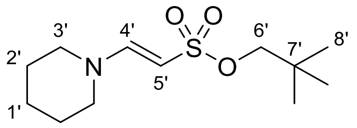

AND

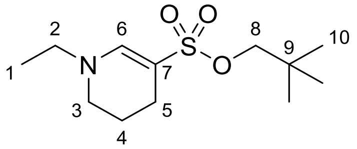

^{1}H NMR (500 MHz, CDCl$_3$, *exo*-product) δ = 7.15 (d, J = 12.8 Hz, 1H, 4'-*H*), 4.77 (d, J = 12.8 Hz, 1H, 5'-*H*), 3.63 (s 2H, 6'-*H*), 3.22 – 3.12 (m, 4H, 3'-H), 1.68 – 1.56 (m, 6H, 1'-*H*, 2'-*H*), 0.95 (s, 9H, 8'-*H*) ppm.
^{1}H NMR (500 MHz, CDCl$_3$, *endo*-product) δ = 7.14 (s, 1H, 6-*H*), 3.54 (s, 2H, 8-*H*), 3.11 – 3.09 (m, 2H, 3-*H*), 2.29 (t, *J* = 6.1 Hz, 2H, 2-*H*), 1.93 – 1.80 (m, 4H, 4-*H*, 5-*H*), 1.16 (t, J = 7.2 Hz, 3H, 1-*H*), 0.95 (s, 9H, 10-*H*) ppm.
^{13}C NMR (126 MHz, CDCl$_3$, *exo/endo-mixture*) δ = 151.1, 145.0, 93.8, 85.1, 77.8, 77.8, 50.5, 44.9, 44.8, 31.7, 31.6, 26.4, 26.4, 26.1, 23.9, 22.7, 22.6, 21.1, 20.0, 13.9 ppm.
HRMS (EI): *m/z* for $C_{12}H_{23}NO_3S^+$ [M]$^+$: calc.: 261.1393, found: 261.1395 (*exo*-product) and 261.1294 (*endo*-product).

Note: An exo/endo-ratio of ca. 4/1 was calculated via ^1H NMR and confirmed by GC measurements.

6.9 Neopentyl (*E*)-2-(azepanyl)ethene sulfonate (3i)

According to GP1, *N*-Ethylazepane (63.6 mg, 64 µL, 500 µmol, 1 eq.), SO_2 stock solution (3.90 mmol, 7.8 eq.) and neopentyl alcohol (229 mg, 2.60 mmol, 5.2 eq.) were used as substrates and the electrolysis was carried out using 11.5 *F*. After purification by flash column chromatography (cyclohexane/ethyl acetate = 97/3 → 77/23), two products (*exo*-product: 9.9 mg, 35.9 µmol, 7%; *endo*-product: 35.2 µmol, 7%) could be isolated as yellowish solids.

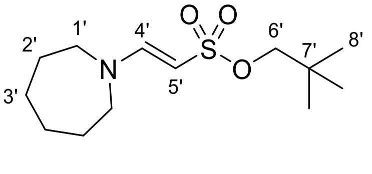

AND

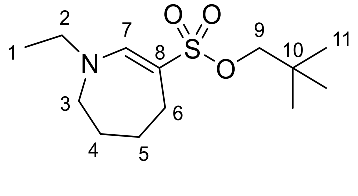

Exo-product:
^{1}H NMR (500 MHz, CDCl$_3$) δ = 7.29 (d, J = 12.6 Hz, 1H, 4'-*H*), 4.68 (d, J = 12.6 Hz, 1H, 5-*H*), 3.63 (s, 2H, 6'-*H*), 3.42 – 3.33 (m, 2H, 1'-*H*), 3.19 – 3.11 (m, 2H, 1'-*H*), 1.79 – 1.68 (m, 4H, 2'-*H*), 1.64 – 1.53 (m, 4H, 3'-*H*), 0.96 (s, 9H, 8'-*H*) ppm.
^{13}C NMR (126 MHz, CDCl$_3$) δ = 151.7, 84.2, 77.8, 55.8, 48.9, 46.3, 31.7, 30.6, 28.3, 27.2, 26.8, 26.5, 26.4, 26.4, 26.3, 25.5, 25.3 ppm.
HRMS (EI): *m/z* for $C_{12}H_{23}NO_3S^+$ [M]$^+$: calc.: 261.1393, found: 261.1394.

Note: Hindered rotation around the N-4' bond caused doublet peaks for some of the carbon atoms in the ^{13}C NMR spectrum.

Endo-product:
^{1}H NMR (500 MHz, CDCl$_3$) δ = 7.17 (s, 1H, 7-*H*), 3.56 (s, 2H, 9-*H*), 3.26 – 3.23 (m, 2H, 3-*H*), 3.20 (q, J = 7.1 Hz, 2H, 2-*H*), 2.48 (dd, J = 7.3, 4.2 Hz, 2H, 6-*H*), 1.81 – 1.76 (m, 4H, 4-*H*, 5-*H),* 1.19 (t, J = 7.1 Hz, 3H, 1-*H*), 0.96 (s, 9H, 11-*H*) ppm.
^{13}C NMR (126 MHz, CDCl$_3$) δ = 148.6, 99.6, 77.8, 54.0, 51.9, 31.7, 28.7, 26.9, 26.5, 26.5, 14.5 ppm.
HRMS (EI): *m/z* for $C_{12}H_{23}NO_3S^+$ [M]$^+$: calc.: 261.1393, found: 261.1395.

Note: An exo/endo-ratio of ca. 1/1 was calculated via the isolated yields.

6.10 Neopentyl *N*-ethyl-1H-pyrrole-3-sulfonate (3j)

According to GP1, *N*-ethylpyrrole (47.6 mg, 53 µL, 500 µmol, 1 eq.), SO_2 stock solution (3.90 mmol, 7.8 eq.) and neopentyl alcohol (229 mg, 2.60 mmol, 5.2 eq.) were used as substrates and the electrolysis was carried out using 17.25 *F*. After twofold purification by flash column chromatography (cyclohexane/ethyl acetate = 98/2 → 91/9, followed by cyclohexane/ethyl acetate = 99/1 → 93/7), the desired product (42.7 mg, 174 µmol, 35%) was obtained as a colorless liquid.

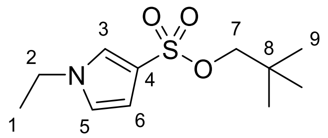

^{1}H NMR (500 MHz, CDCl$_3$) δ = 6.93 (dd, J = 2.7, 1.9 Hz, 1H, 3-*H*), 6.90 (dd, J = 4.0, 1.9 Hz, 1H, 5-*H*), 6.19 (dd, J = 4.0, 2.7 Hz, 1H, 6-*H*), 4.22 (q, J = 7.3 Hz, 2H, 2-*H*), 3.63 (s, 2H, 7-*H*), 1.46 (t, J = 7.3 Hz, 3H, 1-*H*), 0.91 (s, 9H, 9-*H*) ppm.
^{13}C NMR (126 MHz, CDCl$_3$) δ = 127.6, 122.0, 119.7, 108.3, 79.8, 43.5, 31.6, 26.2, 16.7 ppm.
HRMS (EI): *m/z* for $C_{11}H_{19}NO_3S^+$ [M]$^+$: calc.: 245.1080, found: 245.1077.

6.11 Neopentyl *N*-ethyl-1H-indole-2-sulfonate (3k)

According to GP1, *N*-ethylindole (72.6 mg, 73 µL, 500 µmol, 1 eq.), SO_2 stock solution (3.90 mmol, 7.8 eq.) and neopentyl alcohol (229 mg, 2.60 mmol, 5.2 eq.) were used as substrates and the electrolysis was carried out using 11.5 *F*. After purification by flash column chromatography (cyclohexane/ethyl acetate = 98/2 → 65/35), the desired product (51.7 mg, 175 µmol, 35%) was obtained as a colorless liquid.

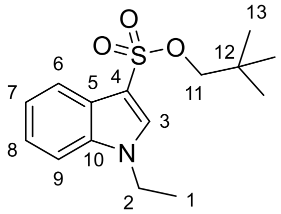

^{1}H NMR (500 MHz, CDCl$_3$) δ = 7.95 – 7.87 (m, 1H, 6-*H*), 7.77 (s, 1H, 3-*H*), 7.42 (dt, J = 8.2, 1.3 Hz, 1H, 9-*H*), 7.35 (ddd, *J* = 8.2, 7.0, 1.3 Hz, 1H, 7-*H*), 7.30 (ddd, *J* = 8.2, 7.0, 1.3 Hz, 1H, 8-*H*) 4.23 (q, J = 7.3 Hz, 2H, 2-*H*), 3.67 (s, 2H, 11-*H*), 1.54 (t, J = 7.3 Hz, 3H, 1-*H*), 0.88 (s, 9H, 13-*H*) ppm.
^{13}C NMR (126 MHz, CDCl$_3$) δ = 136.3, 132.6, 124.5, 123.8, 122.6, 120.3, 110.5, 109.2, 79.2, 42.0, 31.6, 26.2, 15.2 ppm.
HRMS (EI): *m/z* for $C_{15}H_{21}NO_3S^+$ [M]$^+$: calc.: 295.1237, found: 295.1237.

6.12 Neopentyl (*E*)-2-(ethyl(phenyl)amino)ethene sulfonate (3l)

According to GP1, *N,N*-diethylaniline (74.6 mg, 80 µL, 500 µmol, 1 eq.), SO_2 stock solution (3.90 mmol, 7.8 eq.) and neopentyl alcohol (229 mg, 2.60 mmol, 5.2 eq.) were used as substrates and the electrolysis was carried out using 11.5 *F*. After purification by flash column chromatography (cyclohexane/ethyl acetate = 98/2 → 81/19), the desired product (40.2 mg, 135 µmol, 27%) was obtained as a colorless solid.

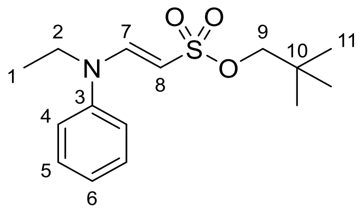

^{1}H NMR (500 MHz, CDCl$_3$) δ = 7.60 (d, J = 12.8 Hz, 1H, 7-*H*), 7.42 – 7.35 (m, 2H, 4-*H*), 7.22 (t, J = 7.5 Hz, 1H, 6-*H*), 7.17 – 7.11 (m, 2H, 5-*H*), 5.08 (d, J = 12.8 Hz, 1H, 8-*H*), 3.68 (q, J = 7.1 Hz, 2H, 2-*H*), 3.67 (s, 2H, 9-*H),* 1.26 (t, J = 7.1 Hz, 3H, 1-*H*), 0.97 (s, 9H, 11-*H*) ppm.
^{13}C NMR (126 MHz, CDCl$_3$) δ = 147.8, 129.9, 129.6, 125.9, 121.9, 90.8, 78.2, 31.7, 29.8, 26.4, 12.0 ppm.
HRMS (EI): *m/z* for $C_{15}H_{23}NO_3S^+$ [M]$^+$: calc.: 297.1393, found: 297.1392.

6.13 Neopentyl (*E*)-2-(ethyl(p-tolyl)amino)ethene sulfonate (3m)

According to GP1, *N,N*-diethy-4-methyllaniline (81.6 mg, 89 µL, 500 µmol, 1 eq.), SO_2 stock solution (3.90 mmol, 7.8 eq.) and neopentyl alcohol (229 mg, 2.60 mmol, 5.2 eq.) were used as substrates and the electrolysis was carried out using 11.5 *F*. After purification by flash column chromatography (cyclohexane/ethyl acetate = 96/4 → 80/10), the desired product (86.0 mg, 276 µmol, 55%) was obtained as a colorless oil.

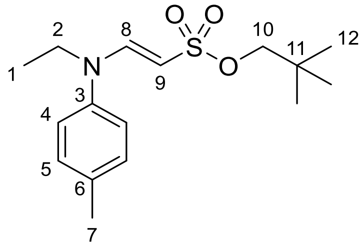

^{1}H NMR (500 MHz, CDCl$_3$) δ = 7.54 (d, J = 13.0 Hz, 1H, 8-*Hz*), 7.20 – 7.15 (m, 2H. 4-*H*), 7.07 – 6.98 (m, 2H, 5-*H*), 5.01 (brs, 1H, 9-*H*), 3.69 – 3.58 (m, 4H, 2-*H*, 10-*H*), 2.34 (s, 3H, 7-*H*), 1.22 (t, J = 7.2 Hz, 3H, 1-*H*), 0.96 (s, 9H, 12-*H*) ppm.
^{13}C NMR (126 MHz, CDCl$_3$) δ = 148.1, 142.1, 135.9, 130.4, 129.9, 121.7, 89.9, 78.1, 31.6, 29.8, 26.3, 20.9 ppm.
HRMS (EI): *m/z* for $C_{16}H_{25}NO_3S^+$ [M]$^+$: calc.: 311.1550, found: 311.1549.

6.14 Neopentyl (*E*)-2-(*N*-methylacetamido)ethene sulfonate (3n)

According to GP1, *N*-methyl-N-vinyl acetamide (49.6 mg, 52 µL, 500 µmol, 1 eq.), SO_2 stock solution (3.90 mmol, 7.8 eq.) and neopentyl alcohol (229 mg, 2.60 mmol, 5.2 eq.) were used as substrates and the electrolysis was carried out using 5.75 *F*. After purification by flash column chromatography (cyclohexane/ethyl acetate = 95/5 → 55/45), the desired product (83.7 mg, 336 µmol, 67%) was obtained as a colorless solid.

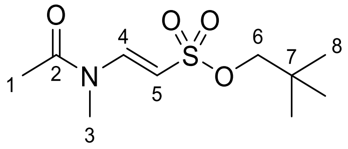

^{1}H NMR (500 MHz, CDCl$_3$) δ = 7.90 (brs, 1H, 4-*H*), 5.60 (d, J = 13.4 Hz, 1H, 5-*H*), 3.75 (s, 2H, 6-*H*), 3.13 (s, 3H, 3-*H*), 2.37 (s, 3H, 1-*H*), 0.98 (s, 9H, 8-*H*) ppm.
^{13}C NMR (126 MHz, CDCl$_3$) δ = 169.9, 143.3, 102.0, 79.3, 31.8, 29.9, 26.3, 21.8 ppm.
HRMS (ESI+): *m/z* for $C_{10}H_{19}NO_4SNa^+$ [M+Na]$^+$: calc.: 272.0927, found 272.0926.

6.15 Neopentyl (*E*)-2-(2-oxopyrrolidinyl)ethene sulfonate (3o)

According to GP1, *N*-vinylpyrrolidinone (55.6 mg, 53 µL, 500 µmol, 1 eq.), SO_2 stock solution (3.90 mmol, 7.8 eq.) and neopentyl alcohol (229 mg, 2.60 mmol, 5.2 eq.) were used as substrates and the electrolysis was carried out using 5.75 *F*. After purification by flash column chromatography (cyclohexane/ethyl acetate = 93/7 → 70/30), the desired product (60.5 mg, 231 µmol, 46%) was obtained as a colorless solid.

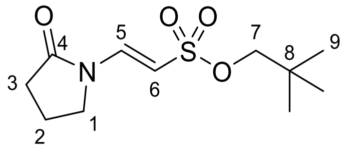

^{1}H NMR (500 MHz, CDCl$_3$) δ = 8.01 (d, J = 13.8 Hz, 1H, 5-*H*), 5.58 (d, J = 13.8 Hz, 1H, 6-*H),* 3.73 (s, 2H, 7-*H*), 3.60 – 3.53 (m, 2H, 1-*H),* 2.59 (dd, J = 8.6, 7.7 Hz, 2H, 3-*H*), 2.26 – 2.18 (m, 2H, 2-*H*), 0.97 (s, 9H, 9-*H*) ppm.
^{13}C NMR (126 MHz, CDCl$_3$) δ = 174.3, 137.6, 103.8, 79.3, 45.2, 31.8, 30.8, 26.3, 17.6 ppm.
HRMS (ESI+): m/z for $C_{11}H_{19}NO_4SNa^+$ [M+Na]$^+$: calc.: 284.0927, found 284.0926.

6.16 Neopentyl (*E*)-2-(2-oxopyrrolidinyl)ethene sulfonate (3p)

According to GP1, *N*-vinylcaprolactam (69.6 mg, 68 µL, 500 µmol, 1 eq.), SO_2 stock solution (3.90 mmol, 7.8 eq.) and neopentyl alcohol (229 mg, 2.60 mmol, 5.2 eq.) were used as substrates and the electrolysis was carried out using 5.75 *F*. After purification by flash column chromatography (cyclohexane/ethyl acetate = 96/4 → 54/56), the desired product (71.3 mg, 246 µmol, 49%) was obtained as a colorless solid.

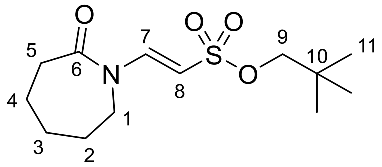

^{1}H NMR (500 MHz, CDCl$_3$) δ = 8.26 (d, J = 14.1 Hz, 1H, 7-*H*), 5.72 (d, J = 14.1 Hz, 1H, 8-*H*), 3.72 (s, 2H, 9-*H*), 3.66 – 3.52 (m, 2H, 1-*H*), 2.81 – 2.62 (m, 2H, 5-*H*), 1.95 – 1.71 (m, 6H, 2-*H*, 3-*H*, 4-*H*), 0.96 (s, 9H, 11-*H*) ppm.
^{13}C NMR (126 MHz, CDCl$_3$) δ = 174.9, 141.1, 102.6, 79.2, 46.8, 37.1, 31.8, 29.3, 27.5, 26.3, 23.4 ppm.
HRMS (ESI+): *m/z* for $C_{13}H_{23}NO_4SNa^+$ [M+Na]$^+$: calc.: 312.1240, found 312.1239.

6.17 Neopentyl (*E*)-2-(9H-carbazol-9-yl)ethene sulfonate (3q)

Similar to GP1, *N*-vinylcarbazole (96.6 mg, 500 µmol, 1 eq.), SO_2 stock solution (3.90 mmol, 7.8 eq., in MeCN) and neopentyl alcohol (229 mg, 2.60 mmol, 5.2 eq.) were used as substrates with benzonitrile as solvent and the electrolysis was carried out using 5.75 *F*. After twofold purification by flash column chromatography (cyclohexane/ethyl acetate = 98/2 → 90/10 followed by cyclohexane/ethyl acetate = 100/0 → 88/12), the desired product (47.5 mg, 138 µmol, 28%) was obtained as a colorless solid.

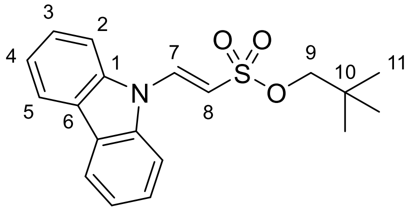

^{1}H NMR (500 MHz, CDCl$_3$) δ = 8.40 (d, J = 14.0 Hz, 1H, 7-*H*), 8.06 (ddd, J = 7.7, 1.2, 0.7 Hz, 2H, 5-*H*), 7.68 (dt, J = 8.4, 0.8 Hz, 2H, 3-*H*), 7.55 (ddd, J = 8.4, 7.3, 1.3 Hz, 2H, 4-*H*), 7.43 (td, J = 7.5, 0.9 Hz, 2H, 2-*H*), 6.60 (d, J = 13.9 Hz, 1H. 8-*H*), 3.85 (s, 2H, 9-*H*), 1.01 (s, 9H, 11-*H*) ppm.
^{13}C NMR (126 MHz, CDCl$_3$) δ = 138.9, 136.9, 127.5, 126.0, 123.7, 120.8, 111.8, 103.9, 79.5, 31.9, 26.3 ppm.
HRMS (EI): m/z for $C_{19}H_{21}NO_3S^+$ [M]$^+$: calc.: 343.1237, found: 343.1240.

Note: Despite extensive purification efforts, the compound could not be isolated in a fully pure state, as indicated by the presence of carbazole as a minor impurity in the NMR spectra. This may also be attributed to potential degradation of the compound during the column chromatography process.

6.18 Methyl (*E*)-2-(2,2,6,6-tetramethylpiperidinyl)ethene sulfonate (4a)

According to GP1, 1-ethyl-2,2,6,6-tetramethylpiperidine (84.7 mg, 500 µmol, 1 eq.), SO_2 stock solution (3.90 mmol, 7.8 eq.) and methanol (83.0 mg, 105 µL, 2.60 mmol, 5.2 eq.) were used as substrates and the electrolysis was carried out using 11.5 *F*. After purification by flash column chromatography (cyclohexane/ethyl acetate = 98/2 → 60/40), the desired product (83.3 mg, 319 µmol, 64%) was obtained as a colorless solid.

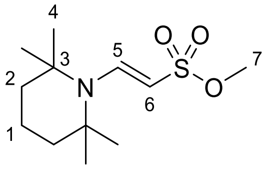

^{1}H NMR (500 MHz, CDCl$_3$) δ = 7.48 (d, J = 13.8 Hz, 1H, 5-*H*), 5.00 (d, J = 13.8 Hz, 1H, 6-*H*), 3.69 (s, 3H, 7-*H*), 1.68 – 1.61 (m, 6H, 1-*H*, 2-*H*), 1.37 (s, 12H, 4-*H*) ppm.
^{13}C NMR (126 MHz, CDCl$_3$) δ = 147.3, 89.7, 58.0, 54.6, 41.0, 28.8, 16.3 ppm.
HRMS (EI): *m/z* for $C_{12}H_{23}NO_3S^+$ [M]$^+$: calc.: 261.1393, found: 261.1396.

6.19 Ethyl (*E*)-2-(2,2,6,6-tetramethylpiperidinyl)ethene sulfonate (4b)

According to GP1, 1-ethyl-2,2,6,6-tetramethylpiperidine (84.7 mg, 500 µmol, 1 eq.), SO_2 stock solution (3.90 mmol, 7.8 eq.) and ethanol (120 mg, 152 µL, 2.60 mmol, 5.2 eq.) were used as substrates and the electrolysis was carried out using 11.5 *F*. After purification by flash column chromatography (cyclohexane/ethyl acetate = 96/4 → 72/28), the desired product (94.7 mg, 344 µmol, 69%) was obtained as a colorless solid.

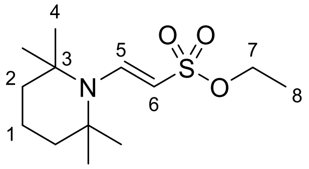

^{1}H NMR (500 MHz, $CDCl_3$) δ = 7.47 (d, J = 13.8 Hz, 1H, 5-*H*), 5.04 (d, J = 13.8 Hz, 1H, 6-*H*), 4.04 (q, J = 7.2 Hz, 2H, 7-*H*), 1.66 – 1.60 (m, 6H, 1-*H*, 2-*H*), 1.37 – 1.32 (m, 15H, 4-*H*, 8-*H*) ppm.
^{13}C NMR (126 MHz, $CDCl_3$) δ = 146.8, 91.2, 64.6, 57.9, 41.0, 28.8, 16.3, 15.0 ppm.
HRMS (EI): *m/z* for $C_{13}H_{25}NO_3S^+$ $[M]^+$: calc.: 275.1550, found: 275.1549.

6.20 ⁿDecyl (*E*)-2-(2,2,6,6-tetramethylpiperidinyl)ethene sulfonate (4c)

According to GP1, 1-ethyl-2,2,6,6-tetramethylpiperidine (84.7 mg, 500 µmol, 1 eq.), SO_2 stock solution (3.90 mmol, 7.8 eq.) and ^{n}decanol (412 mg, 496 µL, 2.60 mmol, 5.2 eq.) were used as substrates and the electrolysis was carried out using 11.5 *F*. After purification by flash column chromatography (cyclohexane/ethyl acetate = 99/1 → 89/11), the desired product (133 mg, 343 µmol, 69%) was obtained as a colorless liquid.

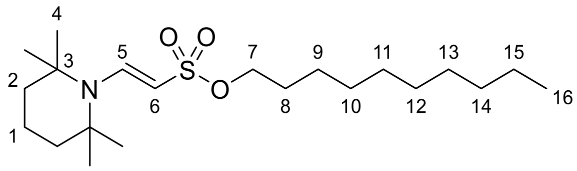

^{1}H NMR (500 MHz, $CDCl_3$) δ = 7.46 (d, J = 13.8 Hz, 1H, 5-*H*), 5.02 (d, J = 13.9 Hz, 1H, 6-*H*), 3.95 (t, J = 6.6 Hz, 2H, 7-*H*), 1.73 – 1.58 (m, 9H, 1-*H*, 2-*H*, 8-*H*, 9-*H*), 1.35 (s, 12H, 4-*H*), 1.33 – 1.18 (m, 13H, 9-15-*H*), 0.87 (t, J = 6.9 Hz, 3H, 16-*H*) ppm.
^{13}C NMR (126 MHz, $CDCl_3$) δ = 146.7, 91.2, 68.8, 57.8, 41.0, 32.0, 29.6, 29.6, 29.4, 29.2, 29.1, 28.8, 25.8, 22.8, 16.3, 14.2 ppm.
HRMS (ESI+): *m/z* for $C_{21}H_{41}NO_3SNa^+$ $[M+Na]^+$: calc.: 410.2699, found: 410.2702.

6.21 2-Methylbutyl (*E*)-2-(2,2,6,6-tetramethylpiperidinyl)ethene sulfonate (4d)

According to GP1, 1-ethyl-2,2,6,6-tetramethylpiperidine (84.7 mg, 500 µmol, 1 eq.), SO_2 stock solution (3.90 mmol, 7.8 eq.) and racemic 2-methylbutanol (229 mg, 281 µL, 2.60 mmol, 5.2 eq.) were used as substrates and the electrolysis was carried out using 11.5 *F*. After purification by flash column chromatography (cyclohexane/ethyl acetate = 99/1 → 84/16), the desired product (135 mg, 426 µmol, 85%) was obtained as a colorless liquid.

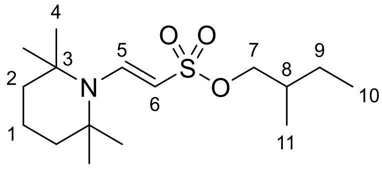

^{1}H NMR (500 MHz, $CDCl_3$) δ = 7.46 (d, J = 13.8 Hz, 1H, 5-*H*), 5.03 (d, J = 13.8 Hz, 1H, 6-*H*), 3.83 (dd, J = 9.4, 5.9 Hz, 1H, 7-*H*), 3.75 (dd, J = 9.4, 6.5 Hz, 1H, 7-*H*), 1.79 – 1.73 (m, 1H, 8-*H*), 1.69 – 1.58 (m, 6H, 1-*H*, 2-*H*), 1.51 – 1.41 (m, 1H, 9-*H*), 1.36 (s, 12H, 4-*H*), 1.28 – 1.14 (m, 1H 9-*H*), 0.94 (d, J = 6.8 Hz, 3H, 11-*H*), 0.89 (t, J = 7.5 Hz, 3H, 10-*H*).
^{13}C NMR (126 MHz, $CDCl_3$) δ = 146.7, 91.2, 73.1, 57.9, 41.1, 34.5, 28.8, 25.8, 16.4, 16.3, 11.3 ppm.
HRMS (ESI+): *m/z* for $C_{16}H_{31}NO_3SNa^+$ $[M+Na]^+$: calc.: 340.1917, found: 340.1918.

6.22 Isopropyl (*E*)-2-(2,2,6,6-tetramethylpiperidinyl)ethene sulfonate (4e)

According to GP1, 1-ethyl-2,2,6,6-tetramethylpiperidine (84.7 mg, 500 µmol, 1 eq.), SO_2 stock solution (3.90 mmol, 7.8 eq.) and isoproanol (156 mg, 200 µL, 2.60 mmol, 5.2 eq.) were used as substrates and the electrolysis was carried out using 11.5 *F*. After purification by flash column chromatography (cyclohexane/ethyl acetate = 98/2 → 85/15), the desired product (83.3 mg, 288 µmol, 58%) was obtained as a colorless solid.

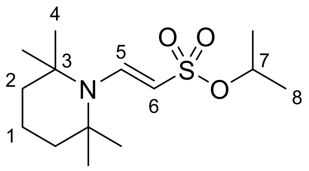

^{1}H NMR (500 MHz, CDCl$_3$) δ = δ 7.47 (d, J = 13.8 Hz, 1H, 5-*H*), 5.08 (d, J = 13.8 Hz, 1H, 6-*H*), 4.57 (hept, J = 6.3 Hz, 1H, 7-*H*), 1.68 – 1.60 (m, 6H, 1-*H*, 2-*H*), 1.36 (s, 12H, 4-*H*), 1.34 (d, J = 6.3 Hz, 6H, 8-*H*) ppm.
^{13}C NMR (126 MHz, CDCl$_3$) δ = 146.2, 92.9, 74.3, 57.8, 41.1, 28.8, 23.2, 16.4 ppm.
HRMS (EI): *m/z* for $C_{14}H_{27}NO_3S^+$ [M]$^+$: calc.: 289.1706, found: 289.1704

6.23 Cyclohexyl (*E*)-2-(2,2,6,6-tetramethylpiperidinyl)ethene sulfonate (4f)

According to GP1, 1-ethyl-2,2,6,6-tetramethylpiperidine (84.7 mg, 500 µmol, 1 eq.), SO_2 stock solution (3.90 mmol, 7.8 eq.) and cyclohexanol (260 mg, 274 µL, 2.60 mmol, 5.2 eq.) were used as substrates and the electrolysis was carried out using 11.5 *F*. After purification by flash column chromatography (cyclohexane/ethyl acetate = 98/2 → 79/21), the desired product (110.6 mg, 336 µmol, 67%) was obtained as a colorless oil.

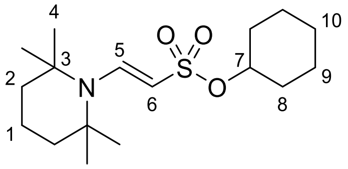

^{1}H NMR (500 MHz, CDCl$_3$) δ = 7.46 (d, J = 13.8 Hz, 1H, 5-*H*), 5.08 (d, J = 13.8 Hz, 1H, 6-*H*), 4.30 (tt, J = 8.8, 3.9 Hz, 1H, 7-*H*), 1.92 (dt, J = 13.7, 4.3 Hz, 2H, 8-*H*), 1.79 – 1.73 (m, 2H, 8-*H*), 1.65 – 1.56 (m, 8H, 1-*H*, 2-*H*, 10-*H*), 1.35 (s, 12H, 4-*H*), 1.35 – 1.19 (m, 4H, 9-*H*) ppm.
^{13}C NMR (126 MHz, CDCl$_3$) δ = 146.1, 93.2, 79.2, 57.8, 41.1, 32.8, 28.8, 25.2, 23.9, 16.4 ppm.
HRMS (ESI+): *m/z* for $C_{17}H_{31}NO_3SNa^+$ [M+Na]$^+$: calc.: 352.1917, found: 352.1916.

6.24 Adamantan-2-yl (*E*)-2-(2,2,6,6-tetramethylpiperidinyl)ethene sulfonate (4g)

According to GP1, 1-ethyl-2,2,6,6-tetramethylpiperidine (84.7 mg, 500 µmol, 1 eq.), SO_2 stock solution (3.90 mmol, 7.8 eq.) and 2-adamantol (396 mg, 2.60 mmol, 5.2 eq.) were used as substrates and the electrolysis was carried out using 11.5 *F*. After twofold purification by flash column chromatography (cyclohexane/ethyl acetate = 98/2 → 82/18 followed by cyclohexane/ethyl acetate = 99/1 → 84/16), the desired product (110 mg, 289 µmol, 58%) was obtained as a colorless solid.

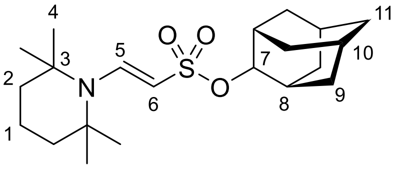

^{1}H NMR (500 MHz, CDCl$_3$) δ = 7.46 (d, J = 13.8 Hz, 1H, 5-*H*), 5.09 (d, J = 13.8 Hz, 1H, 6-*H*), 4.47 (d, J = 3.5 Hz, 1H, 7-*H*), 2.16 – 2.10 (m, 4H, Ad-*H*), 1.92 – 1.79 (m, 4H, Ad-*H*), 1.76 – 1.67 (m, 6H, Ad-*H*), 1.66 – 1.59 (m, 6H, 1-*H*, 2-*H*), 1.35 (s, 12H, 4-*H*) ppm.
^{13}C NMR (126 MHz, CDCl$_3$) δ = 146.0, 93.4, 83.8, 57.7, 57.1, 41.1, 37.4, 36.8, 36.7, 36.5, 35.5, 34.7, 33.2, 33.0, 31.4, 31.2, 28.8, 27.9, 27.1, 26.9, 16.4.
HRMS (ESI+): *m/z* for $C_{21}H_{35}NO_3SNa^+$ [M+Na]$^+$: calc.: 404.2230, found: 404.2230.

Note: Despite extensive purification efforts, the compound could not be isolated in a fully pure state, as indicated by the presence of minor impurities in the NMR spectra. This may also be attributed to potential degradation of the compound during the column chromatography process.

6.25 *S*-Methyllactyl (*E*)-2-(2,2,6,6-tetramethylpiperidinyl)ethene sulfonate (4h)

According to GP1, 1-ethyl-2,2,6,6-tetramethylpiperidine (84.7 mg, 500 µmol, 1 eq.), SO_2 stock solution (3.90 mmol, 7.8 eq.) and *S*-methyl lactate (271 mg, 246 µL, 2.60 mmol, 5.2 eq.) were used as substrates and the electrolysis was carried out using 11.5 *F*. After twofold purification by flash column chromatography (cyclohexane/ethyl acetate = 98/2 → 66/34 followed by cyclohexane/ethyl acetate = 97/3 → 58/42), the desired product (63.0 mg, 189 µmol, 38%) was obtained as a colorless liquid.

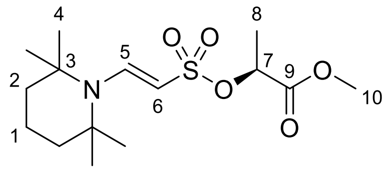

^{1}H NMR (500 MHz, $CDCl_3$) δ = 7.49 (d, J = 13.8 Hz, 1H, 5-*H*), 5.07 (d, J = 13.8 Hz, 1H, 6-*H*), 4.79 (q, J = 7.0 Hz, 1H, 7-*H*), 3.75 (s, 3H, 10-*H*), 1.66 – 1.61 (m, 6H, 1-*H*, 2-*H*), 1.55 (d, J = 7.0 Hz, 3H, 8-*H*), 1.35 (d, J = 2.8 Hz, 12H, 4-*H*) ppm.
^{13}C NMR (126 MHz, $CDCl_3$) δ = 171.0, 147.3, 91.0, 72.6, 58.1, 52.6, 40.9, 28.9, 18.8, 16.3 ppm.
HRMS (EI): *m/z* for $C_{15}H_{27}NO_5S^+$ [M]$^+$: calc.: 333.1604, found: 333.1605.

6.26 *N*-Butyloxycarbonylprolinyl (*E*)-2-(2,2,6,6-tetramethylpiperidinyl)ethene sulfonate (4i)

According to GP1, 1-ethyl-2,2,6,6-tetramethylpiperidine (84.7 mg, 500 µmol, 1 eq.), SO_2 stock solution (3.90 mmol, 7.8 eq.) and *N*-Boc prolinol (523 mg, 2.60 mmol, 5.2 eq.) were used as substrates and the electrolysis was carried out using 11.5 *F*. After twofold purification by flash column chromatography (cyclohexane/ethyl acetate = 97/3 → 79/21 followed by cyclohexane/acetone = 97/3 → 85/15), the desired product (133 mg, 308 µmol, 62%) was obtained as a colorless solid.

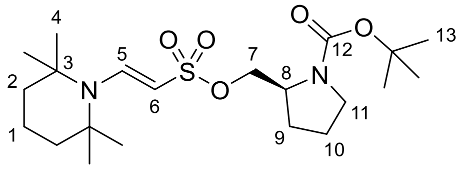

^{1}H NMR (500 MHz, CD_3CN) δ = 7.43 (d, J = 13.8 Hz, 1H, 5-*H*), 5.07 (dd, J = 14.2, 8.2 Hz, 1H, 6-*H*), 4.03 – 3.88 (m, 2H, 7-*H*), 3.82 (t, J = 9.0 Hz, 1H, 8-*H*), 3.32 – 3.20 (m, 2H, 11-*H*), 2.00 – 1.75 (m, 4H, 9-*H*, 10-*H*), 1.70 – 1.58 (m, 6H, 1-*H*, 2-*H*), 1.42 (s, 9H, 13-*H*), 1.34 (s, 12H, 4-*H)* ppm.
^{13}C NMR (126 MHz, CD_3CN) δ = 155.2, 154.9, 147.9, 147.8, 118.3, 91.0, 90.7, 79.9, 79.8, 69.4, 68.7, 58.7, 56.6, 47.7, 47.3, 41.2, 29.2, 28.9, 28.9, 28.6, 28.4, 24.4, 23.5, 16.7 ppm.
HRMS (ESI+): *m/z* for $C_{21}H_{38}N_2O_5SNa^+$ [M+Na]$^+$: calc.: 453.2394, found: 453.2393.

Note: Due to hindered rotation around the amide bond N-12, doublets were observed in the ^{13}C NMR spectra for some of the signals.

6.27 Tert-butyl (*E*)-4-(((2-(2,2,6,6-tetramethylpiperidinyl)vinyl)sulfonyl)oxy)piperidine carboxylate (4j)

According to GP1, 1-ethyl-2,2,6,6-tetramethylpiperidine (84.7 mg, 500 µmol, 1 eq.), SO_2 stock solution (3.90 mmol, 7.8 eq.) and *N*-Boc piperidinol (523 mg, 2.60 mmol, 5.2 eq.) were used as substrates and the electrolysis was carried out using 11.5 *F*. After twofold purification by flash column chromatography (cyclohexane/ethyl acetate = 97/3 → 72/28 followed by cyclohexane/ethyl acetate = 98/2 → 72/28), the desired product (101 mg, 235 µmol, 47%) was obtained as a colorless oil.

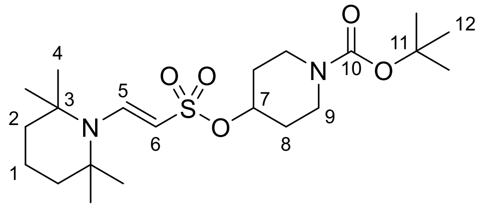

^{1}H NMR (500 MHz, $CDCl_3$) δ = 7.49 (d, J = 13.8 Hz, 1H, 5-*H*), 5.08 (d, J = 13.8 Hz, 1H, 6-*H),* 4.51 (tt, J = 7.5, 3.7 Hz, 1H, 7-*H*), 3.65 (ddd, J = 12.0, 6.8, 3.7 Hz, 2H, 9-*H*), 3.29 (ddd, J = 13.5, 7.6, 3.8 Hz, 2H, 9-*H*), 1.92 – 1.83 (m, 2H, 8-*H*), 1.82 – 1.72 (m, 2H, 8-*H*), 1.69 – 1.59 (m, 6H, 1-*H*, 2-*H*), 1.45 (s, 9H, 12-*H*), 1.36 (s, 12H, 4-*H*) ppm.
^{13}C NMR (126 MHz, $CDCl_3$) δ = 154.9, 146.5, 92.5, 79.9, 75.5, 58.0, 41.0, 31.7, 28.9, 28.5, 16.3 ppm.
HRMS (ESI+): *m/z* for $C_{21}H_{38}N_2O_5SNa^+$ [M+Na]$^+$: calc.: 453.2394, found: 453.2394.

6.28 Cyclohex-3-enyl (*E*)-2-(2,2,6,6-tetramethylpiperidin-1-yl)ethene sulfonate (4k)

According to GP1, 1-ethyl-2,2,6,6-tetramethylpiperidine (84.7 mg, 500 µmol, 1 eq.), SO_2 stock solution (3.90 mmol, 7.8 eq.) and 3-cyclohexenol (255 mg, 258 µL, 2.60 mmol, 5.2 eq.) were used as substrates and the electrolysis was carried out using 11.5 *F*. After purification by flash column chromatography (cyclohexane/ethyl acetate = 98/2 → 76/24), the desired product (33.8 mg, 103 µmol, 21%) was obtained as a colorless liquid.

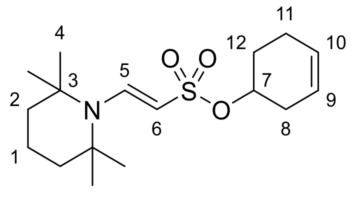

^{1}H NMR (500 MHz, $CDCl_3$) δ = 7.47 (d, J = 13.8 Hz, 1H, 5-*H*), 5.69 – 5.61 (m, 1H, 9-*H*), 5.58 – 5.50 (m, 1H, 10-*H*), 5.09 (d, J = 13.8 Hz, 1H, 6-*H*), 4.55 (dddd, J = 9.8, 7.4, 5.4, 3.2 Hz, 1H, 7-*H*), 2.48 – 2.33 (m, 1H, 8-*H*), 2.32 – 2.17 (m, 2H, 8-*H*, 11-*H*), 2.16 – 2.03 (m, 1H, 11-*H*), 2.03 – 1.93 (m, 1H, 12-*H*), 1.90 – 1.78 (m, 1H, 12-*H*), 1.69 – 1.56 (m, 6H, 1-*H*, 2-*H*), 1.35 (s, 12H, 4-*H*) ppm.
^{13}C NMR (126 MHz, $CDCl_3$) δ = 146.2, 126.9, 123.5, 92.8, 76.0, 57.8, 41.0, 31.9, 28.8 (d), 28.5, 23.6, 16.3 ppm.
HRMS (ESI+): *m/z* for $C_{17}H_{29}NO_3SNa^+$ [M+Na]$^+$: calc.: 350.1760, found: 350.1759.

6.29 (*E*)-4-((2-(2,2,6,6-tetramethylpiperidinyl)vinyl)sulfonyl)morpholine (5a)

According to GP1, 1-ethyl-2,2,6,6-tetramethylpiperidine (84.7 mg, 500 µmol, 1 eq.), SO_2 stock solution (3.90 mmol, 7.8 eq.) and morpholine (227 mg, 227 µL, 2.60 mmol, 5.2 eq.) were used as substrates and the electrolysis was carried out using 11.5 *F*. After purification by flash column chromatography (cyclohexane/ethyl acetate = 96/4 → 50/50), the desired product (17.7 mg, 55.9 µmol, 11%) was obtained as a colorless oil.

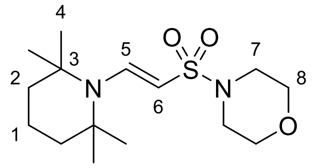

^{1}H NMR (400 MHz, $CDCl_3$) δ = 7.32 (d, J = 13.8 Hz, 1H, 5-*H*), 4.93 (d, J = 13.8 Hz, 1H, 6-*H*), 3.78 (t, J = 4.3 Hz, 4H, 8-*H*), 3.00 (t, J = 4.6 Hz, 4H, 7-*H*), 1.69 – 1.59 (m, 6H, 1-*H*, 2-*H*), 1.36 (s, 12H, 4-*H*) ppm.
HRMS (ESI+): *m/z* for $C_{15}H_{28}N_2O_3SNa^+$ [M+Na]$^+$: calc.: 339.1713, found: 339.1716.

Note: An ^{1}H NMR yield of 44% was calculated by addition of 1,3,5-trimethoxybenzene as internal standard to the reaction mixture after the electrolysis. We therefore suspect a rapid degradation of the enaminyl sulfonamide product during workup and column chromatography. The synthetic application of an amidosulfinate-intermediate formed from an amine and SO_2 with the help of a base, similar to the monoalkylsulfite, has been demonstrated before, forming stable alkyl aryl sulfonamides.[6] Enaminyl sulfonamides on the other hand seem to be unstable, probably due to rapid hydrolysis. Due to degradation and the small quantity of the isolated product, a ^{13}C NMR could not be measured in sufficient quality.

6.30 (1*R*,5*S*)-8-(((*E*)-2-(2,2,6,6-tetramethylpiperidinyl)vinyl)sulfonyl)-8-azabicyclo[3.2.1]octane (5b)

According to GP1, 1-ethyl-2,2,6,6-tetramethylpiperidine (84.7 mg, 500 µmol, 1 eq.), SO_2 stock solution (3.90 mmol, 7.8 eq.) and 8-azabicyclo[3.2.1]octane hydrochloride (384 mg, 2.60 mmol, 5.2 eq.) were used as substrates. Differently to GP1, 1,8-diaza-bicyclo[5.4.0]undec-7-ene (DBU, 1.08 g, 1.06 mL, 7.10 mmol, 14.2 eq.) was used in excess to account for the additional hydrochloride from 8-azabicyclo[3.2.1]octane salt. The electrolysis was carried out using 11.5 *F*. After purification by flash column chromatography (cyclohexane/ethyl acetate = 98/2 → 71/29), the desired product (11.2 mg, 32.9 µmol, 7%) was obtained as a colorless oil.

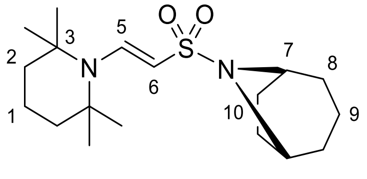

^{1}H NMR (400 MHz, $CDCl_3$) δ = 7.38 (d, J = 13.9 Hz, 1H, 5-*H*), 4.99 (d, J = 13.9 Hz, 1H, 6-*H*), 4.08 (dq, J = 5.4, 2.9 Hz, 2H, 7-*H*), 2.05 – 1.95 (m, 2H, 10-*H*), 1.88 – 1.77 (m, 2H, 8-*H*), 1.75 – 1.43 (m, 12H, 1-*H*, 2-*H*, 8-*H*, 9-*H*, 10-*H)*, 1.33 (s, 12H, 4-*H*) ppm.
HRMS (ESI+): *m/z* for $C_{18}H_{32}N_2O_2SNa^+$ [M+Na]$^+$: calc.: 363.2077, found: 363.2080z.

Note: We suspect similar degradation of the enaminyl sulfonamide, due to which only a small quantity of the desired product was isolated. A ^{13}C NMR could not be measured in sufficient quality.

6.31 Limitations of the Scope/Unsuccessful substrates

The following substrates were also tried, but for the reason stated below, the desired product could not be isolated in sufficient quantities.

(En)amines:

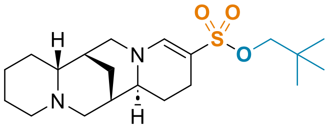

degradation of s.m.
from (-)-sparteine

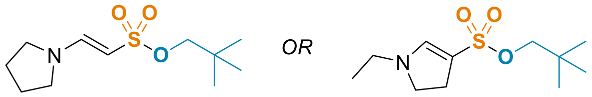

overoxidation to pyrrole; complex product mixture
from N-ethylpyrrolidine

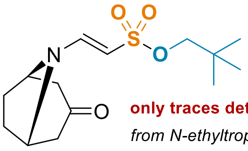

only traces detected
from N-ethyltropinone

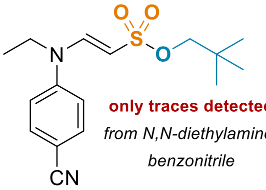

only traces detected
from N,N-diethylamino-benzonitrile

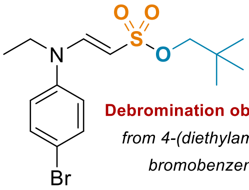

Debromination observed
from 4-(diethylamino)-bromobenzene

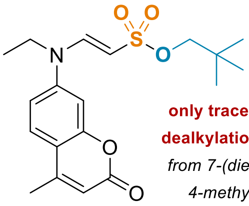

only traces detected
dealkylation observed
from 7-(diethylamino)-4-methylcumarin

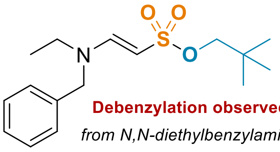

Debenzylation observed
from N,N-diethylbenzylamine

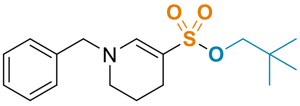

Debenzylation observed
from N-benzylpiperidine

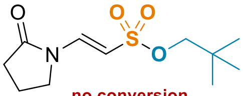

no conversion
from N-ethylpyrrolidinone

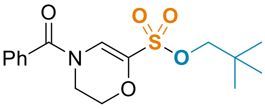

no conversion
from morpholino(phenyl)methanone

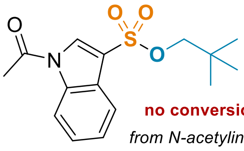

no conversion
from N-acetylindole

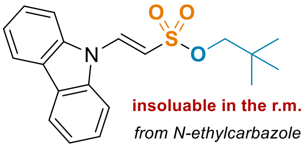

insoluable in the r.m.
from N-ethylcarbazole

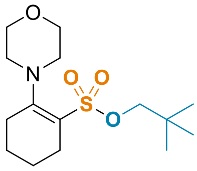

hydrolysis of enamine observed
from 1-morpholino-cyclohexene

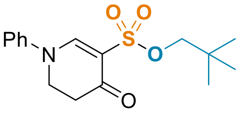

formation of enamine,
but no sulfonylation
from N-phenylpiperidinone

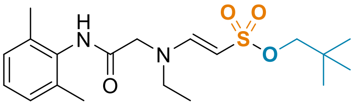

degredation (dealkylation) of s.m.
from lidocaine

Alcohols:

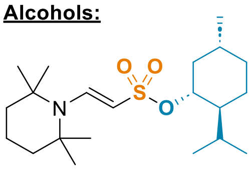

product detected; degradation during column chromatography
from (-)-menthol

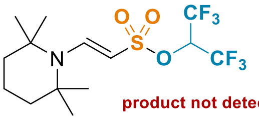

product not detected
from hexafluoroisopropanol

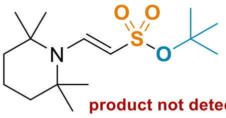

product not detected
no conductivity
from tert.-butanol

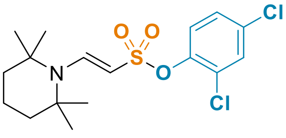

product not detected
complex product mixture
dimers observed
from 2,4-dichlorophenol

Other nucleophiles:

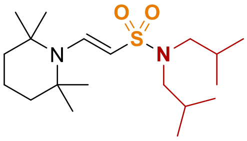

product detected; isolation via column chromatography failed
from diisobutylamine

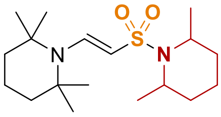

product not detected
from 2,6-dimethylpiperidine

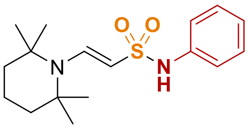

product not detected
from aniline

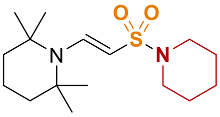

product detected; isolation via column chromatography failed
from piperidine

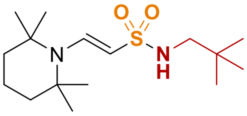

product detected; isolation via column chromatography failed
from neopentylamine

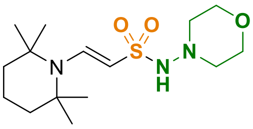

product not detected
from 4-aminomorphiline

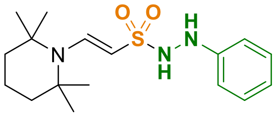

product not detected
from phenylhydrazine

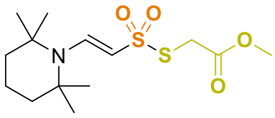

product not detected
from methylthioglycolat

Figure S11 : Unsuccessful substrates.

7 Crystallographic Data

Crystallization was carried out by dissolving compound **3d** in ^{n}hexane using heat and a vortexer. Slow but incomplete (!) evaporation of the solvent resulted in crystal formation.

Table S10: Crystallographic data for neopentyl (*E*)-2-(2,2,6,6-tetramethylpiperidinyl)ethene sulfonate (**3d**).

Parameter	Value
CCDC Number	2407541
Empirical formular	$C_{16}H_{31}NO_3S$
Formular weight	317.48 g/mol
Temperature	100(2) K
Wavelength	0.71073 Å
Crystal system	Monoclinic
Crystal size and habit	0.061 x 0.099 x 0.384 mm clear colourless needle
Space group name	P 1 21/c 1 (Nr. 14)
Unit cell dimensions	a = 6.3217(2) Å α = 90°
	b = 14.4116(4) Å β = 97.7009(9)°
	c = 19.7403(5) Å γ = 90°
Volume	1782.24(9) $Å^3$
Number of reflections	114870
Z	4
Density (calculated)	1.183 g×cm^{-3}
Absorption coefficient	0.191/mm
Method of absorption correction	Numerical Mu From Formula
Transmission (min. and max.)	0.9300 and 0.9880
F(000)	696
Theta range for data collection	2.08° ≤ Θ ≤ 36.36°
Index ranges	-10 ≤ h ≤ 10, -24 ≤ k ≤ 24, -32 ≤ l ≤ 32
Number of reflections collected (indipendend)	114870 (8673); R(int) = 0.0367
observed [I>2sigma(I)]	7732
Refinement method	Full-matrix least-squares on F^2 (SHELXL-2019/1)
Data / restraints / parameters	8673 / 26 / 234
Goodness-of-fit on F^2	1.028
Final R indices [I>2sigma(I)]	R1 = 0.0343, wR2 = 0.0949
R indices (all data)	R1 = 0.0390, wR2 = 0.0997
Largest diff. peak and hole	0.537 and -0.385 e$Å^{-3}$

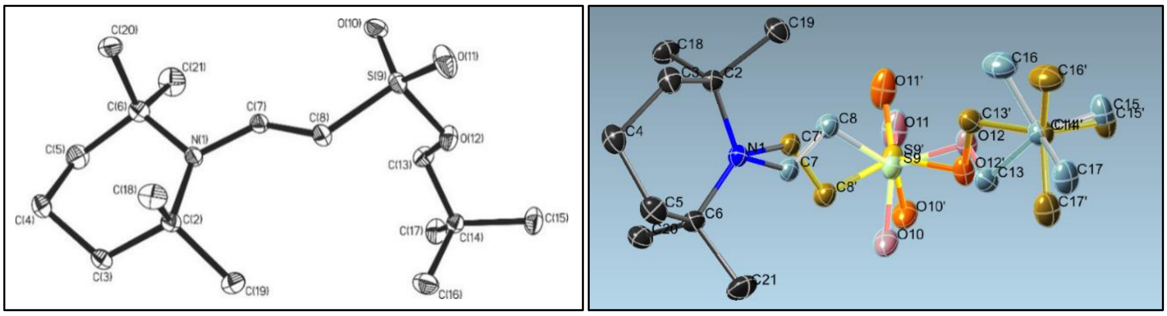

Figure S12: Molecular structure of neopentyl (*E*)-2-(2,2,6,6-tetramethylpiperidinyl)ethene sulfonate (**3d**) as determined by single crystal X-ray analysis. The side chain of the Me_4-Piperidine unit is disordered on two positions. Refinement results in an occupation ratio of approx. 0.85/0.15. The ORTEP diagram is drawn at the 40% probability level.

8 NMR Spectra

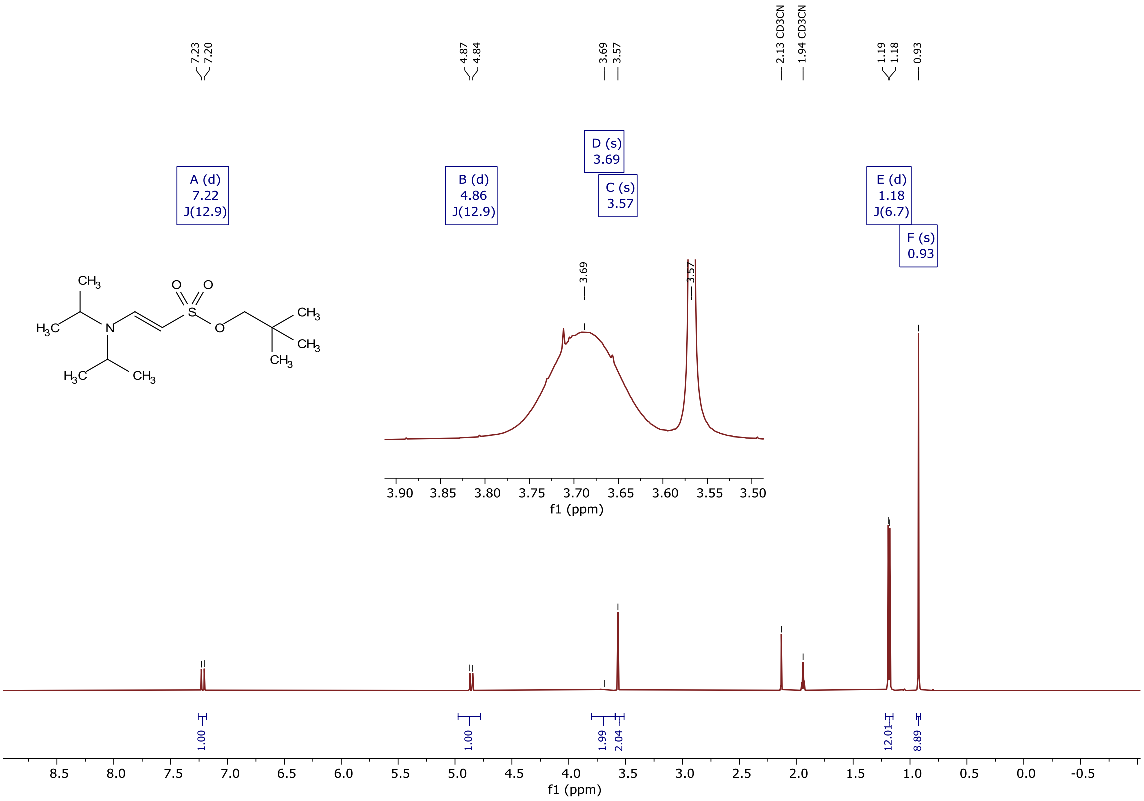

Figure S13: ^{1}H NMR spectrum (500 MHz, 25°C, CD_3CN) of **3a**.

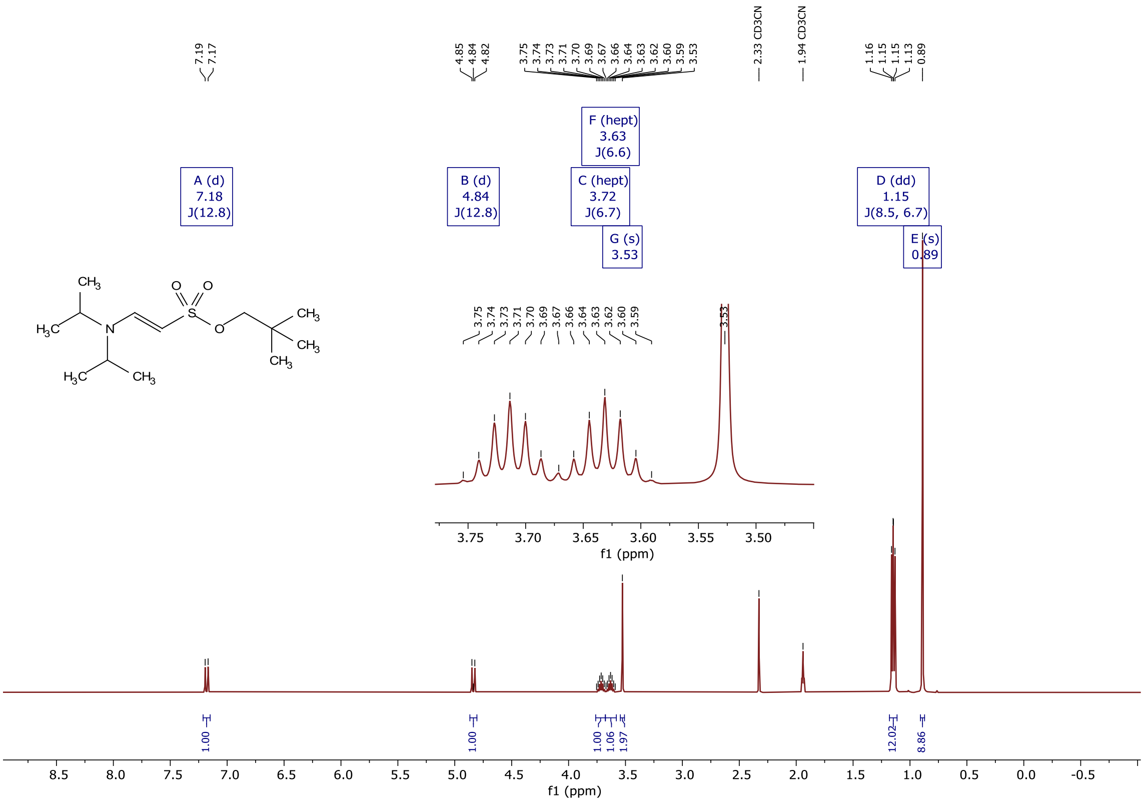

Figure S14: ^{1}H NMR spectrum (500 MHz, -30°C, CD_3CN) of **3a**.

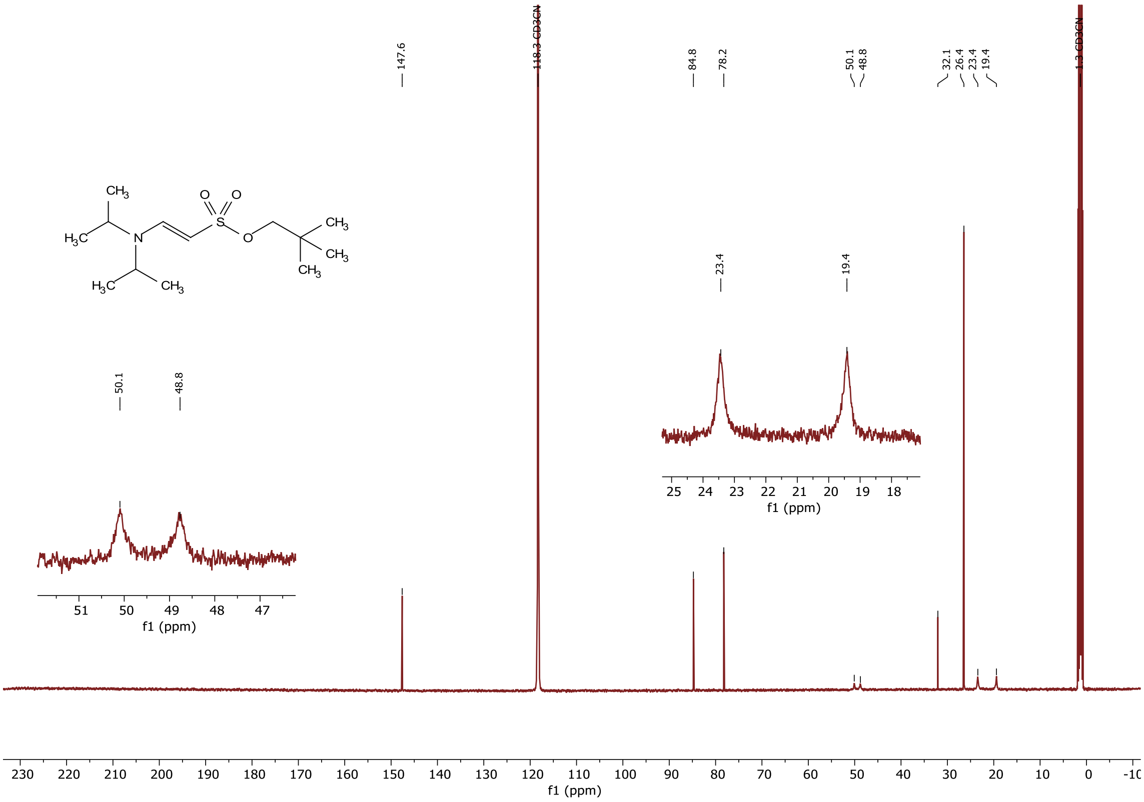

Figure S15: ^{13}C NMR spectrum (126 MHz, 25°C, CD_3CN) of **3a**.

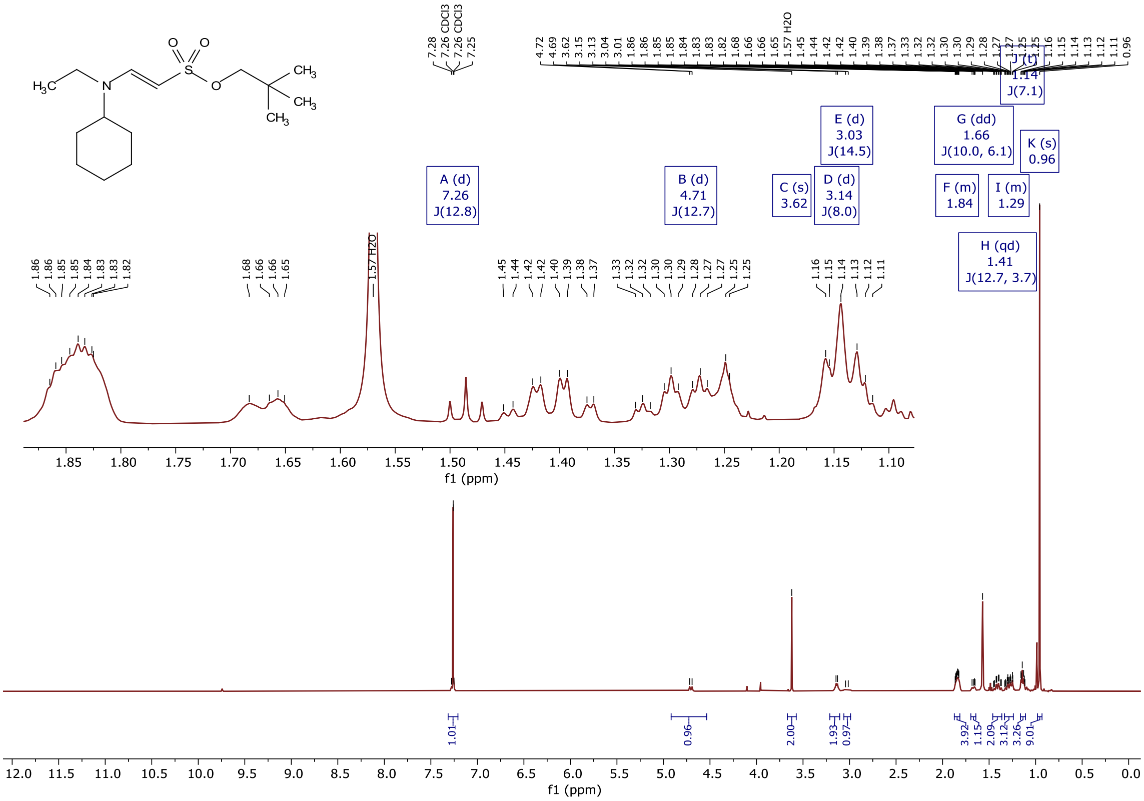

Figure S16: ^{1}H NMR spectrum (500 MHz, $CDCl_3$) of **3b**.

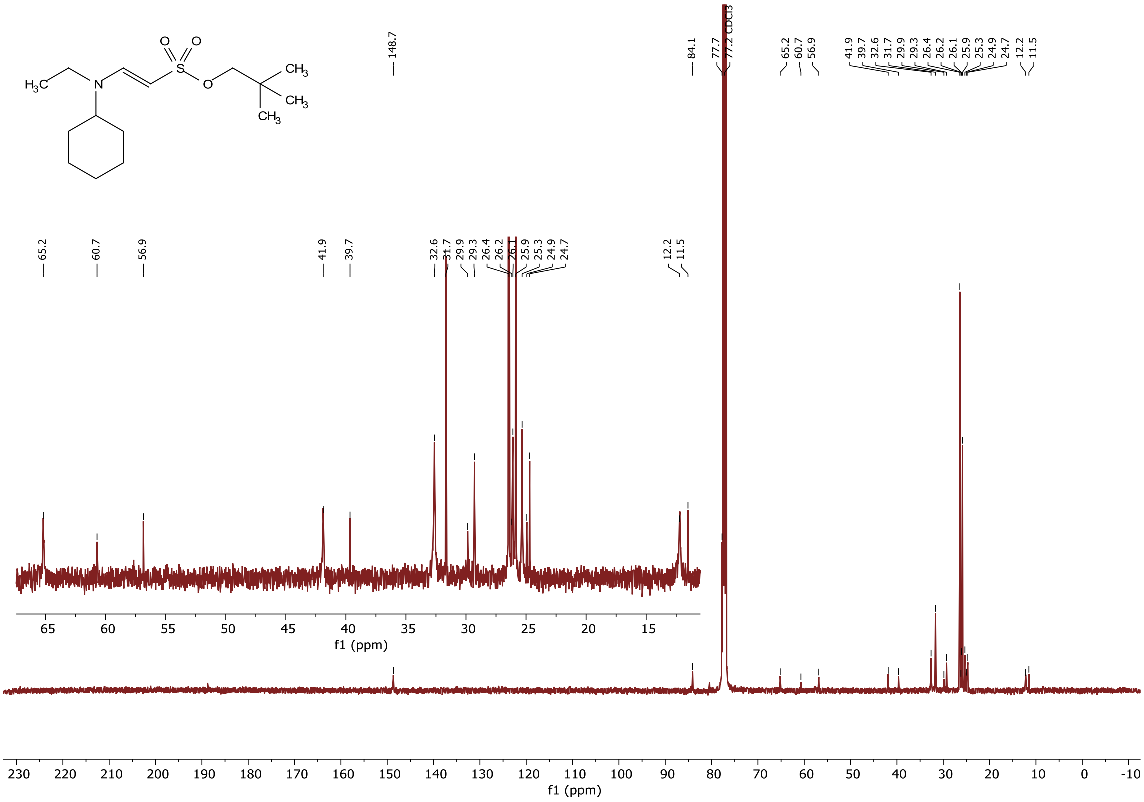

Figure S17: ^{13}C NMR spectrum (126 MHz, $CDCl_3$) of **3b**.

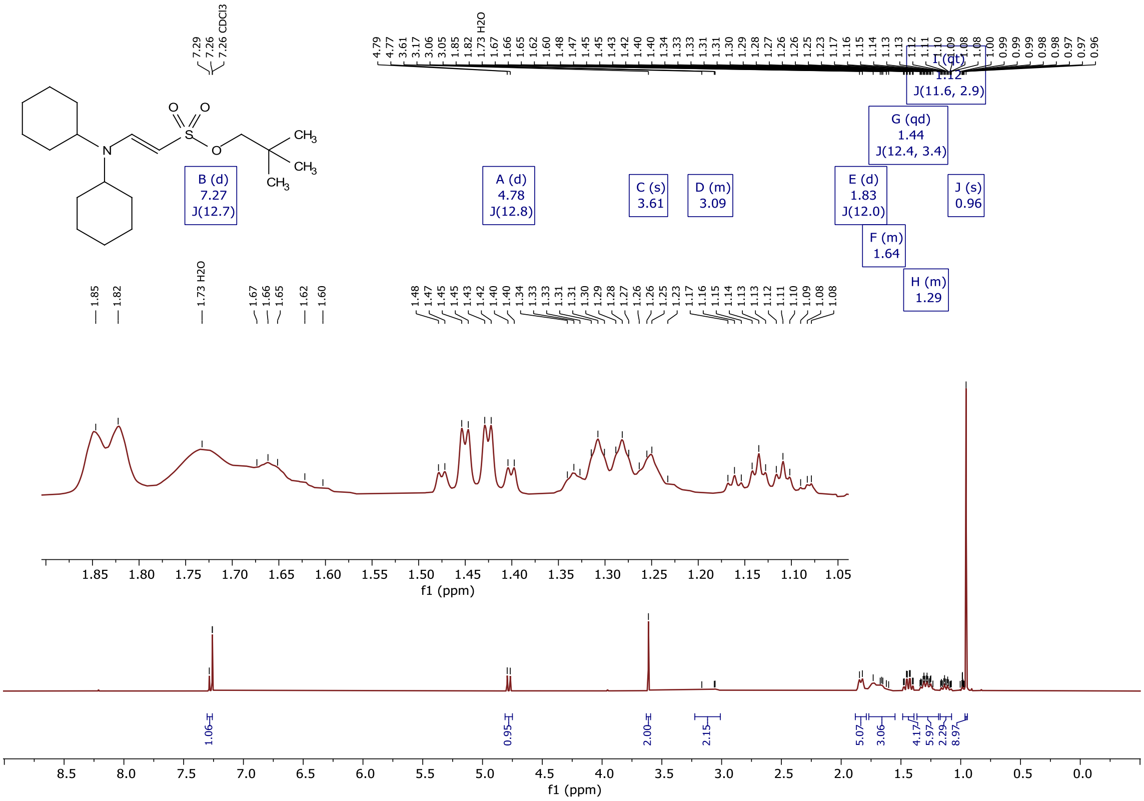

Figure S18: ^{1}H NMR spectrum (500 MHz, $CDCl_3$) of **3c**.

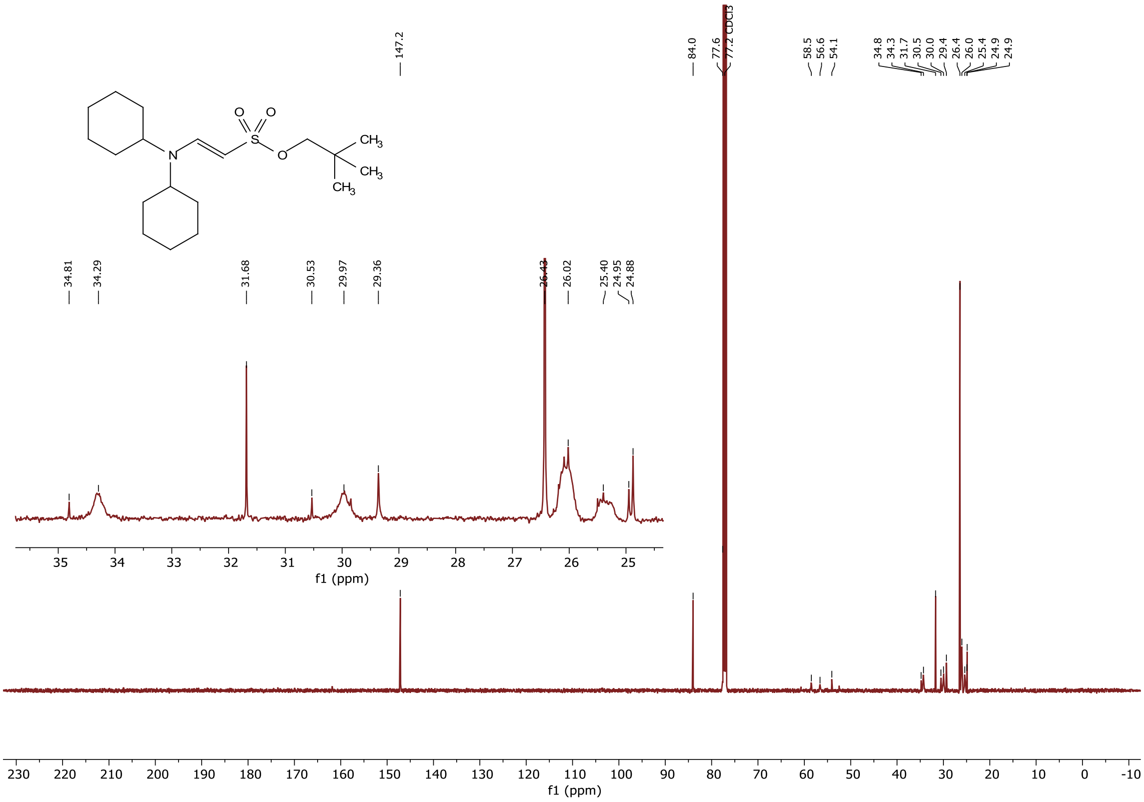

Figure S19: ^{13}C NMR spectrum (126 MHz, $CDCl_3$) of **3c**.

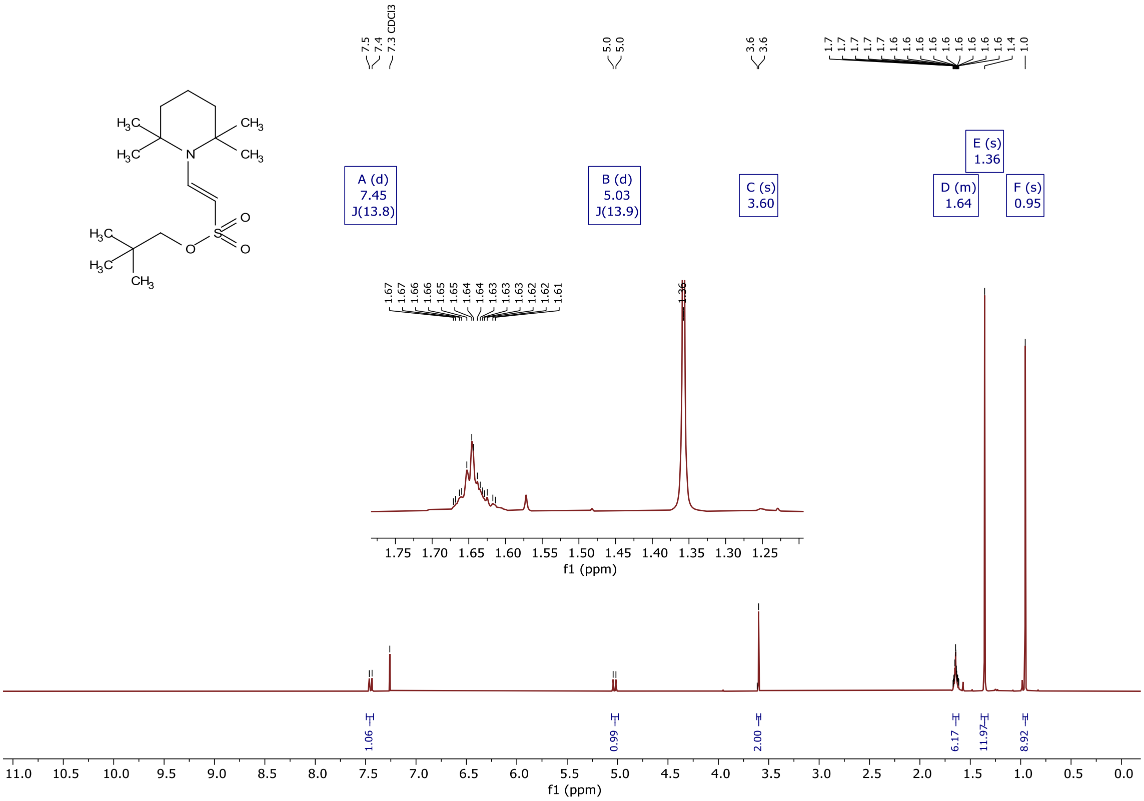

Figure S20: ^{1}H NMR spectrum (500 MHz, $CDCl_3$) of **3d**.

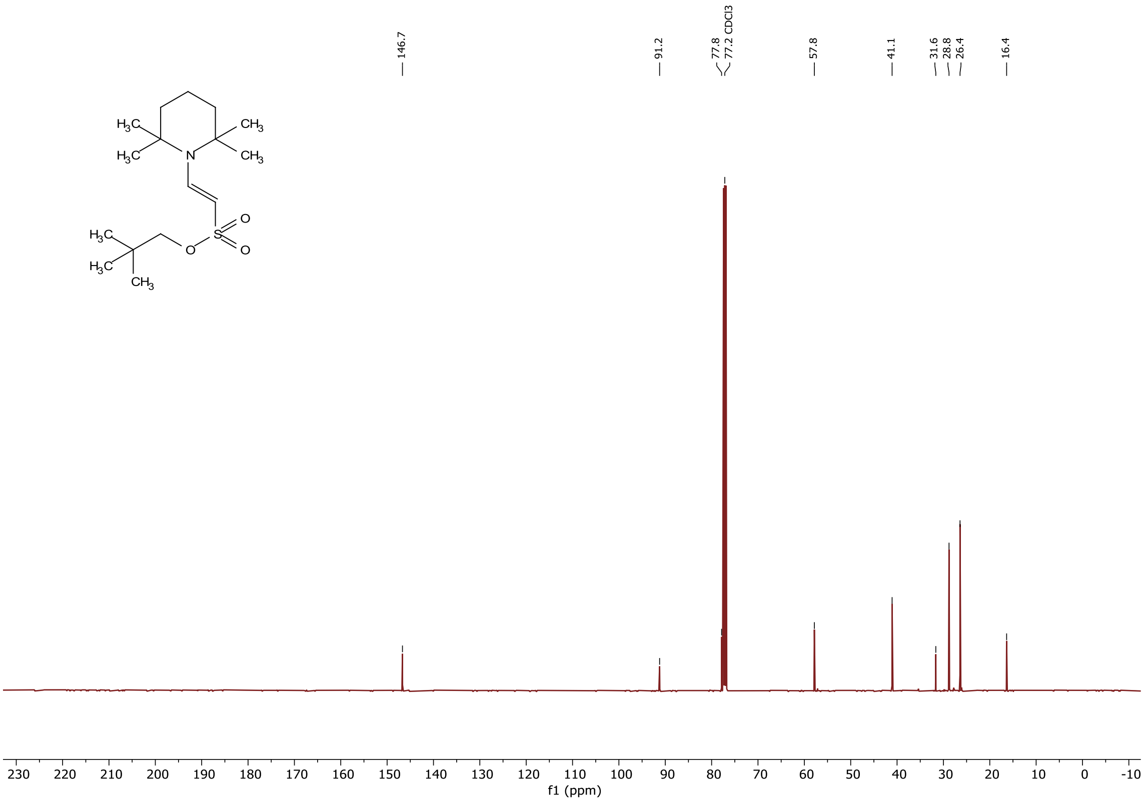

Figure S21: ^{13}C NMR spectrum (126 MHz, $CDCl_3$) of **3d**.

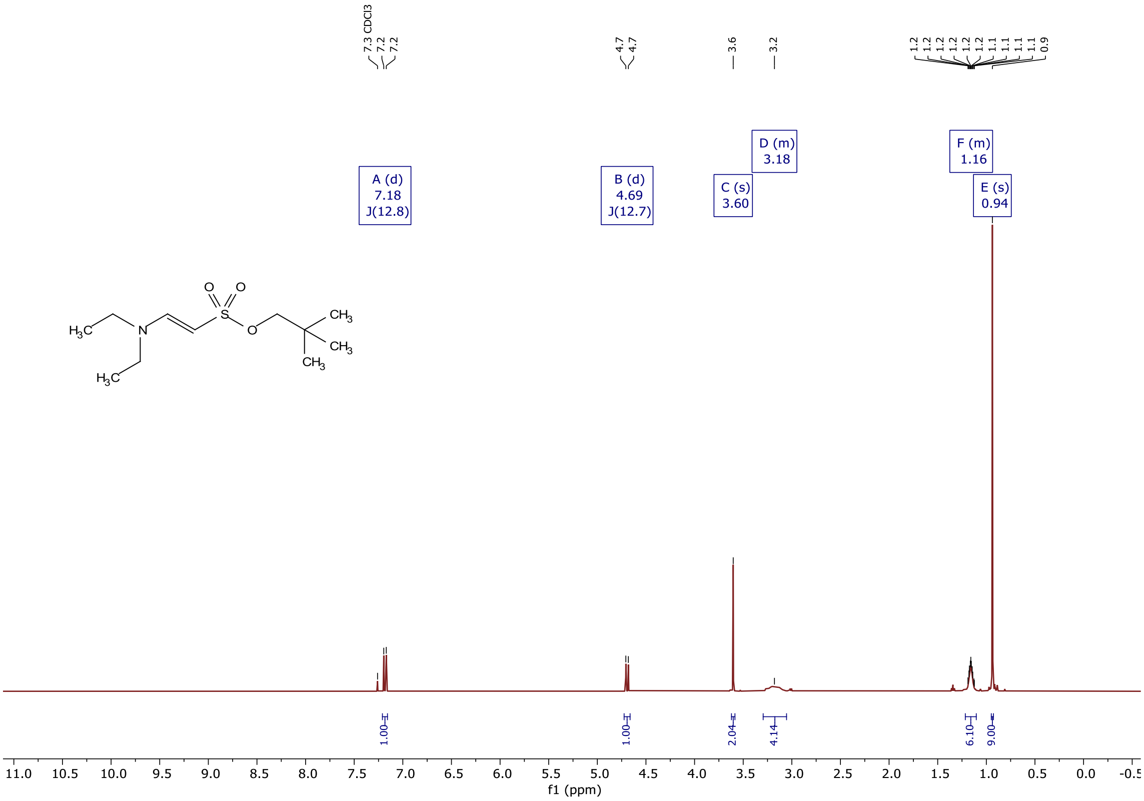

Figure S22: ^{1}H NMR spectrum (500 MHz, $CDCl_3$) of **3e**.

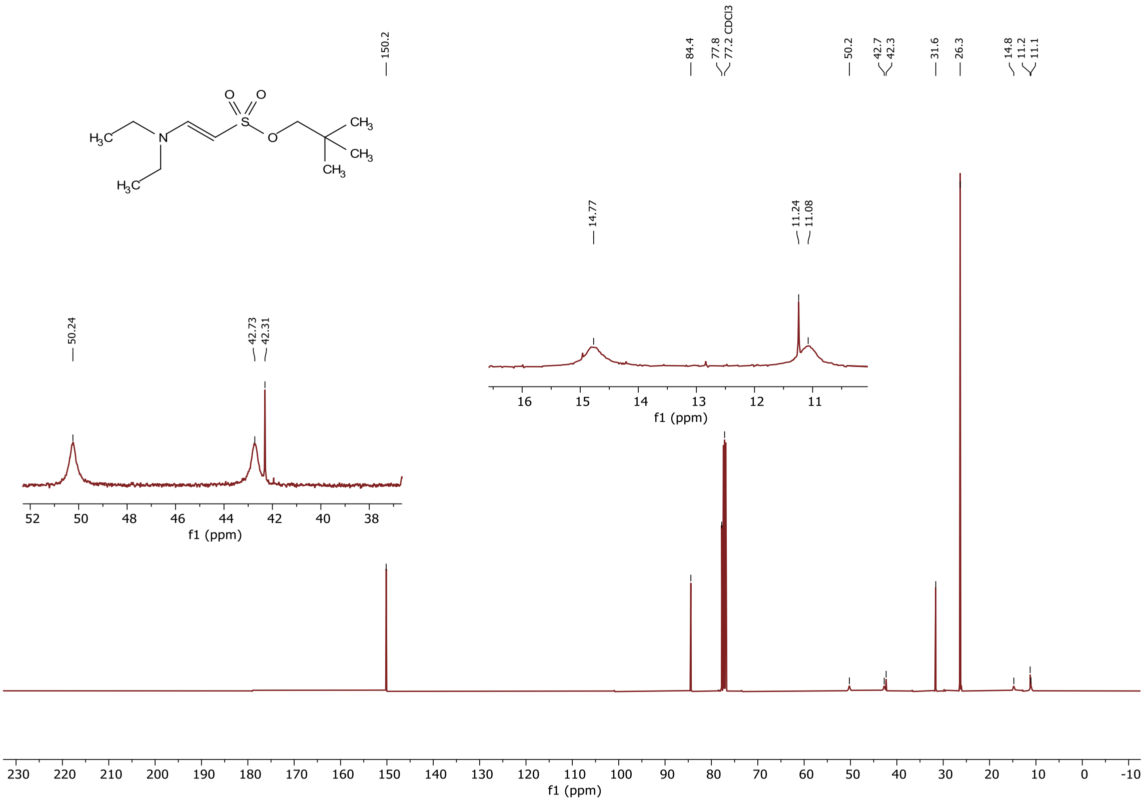

Figure S23: ^{13}C NMR spectrum (126 MHz, $CDCl_3$) of **3e**.

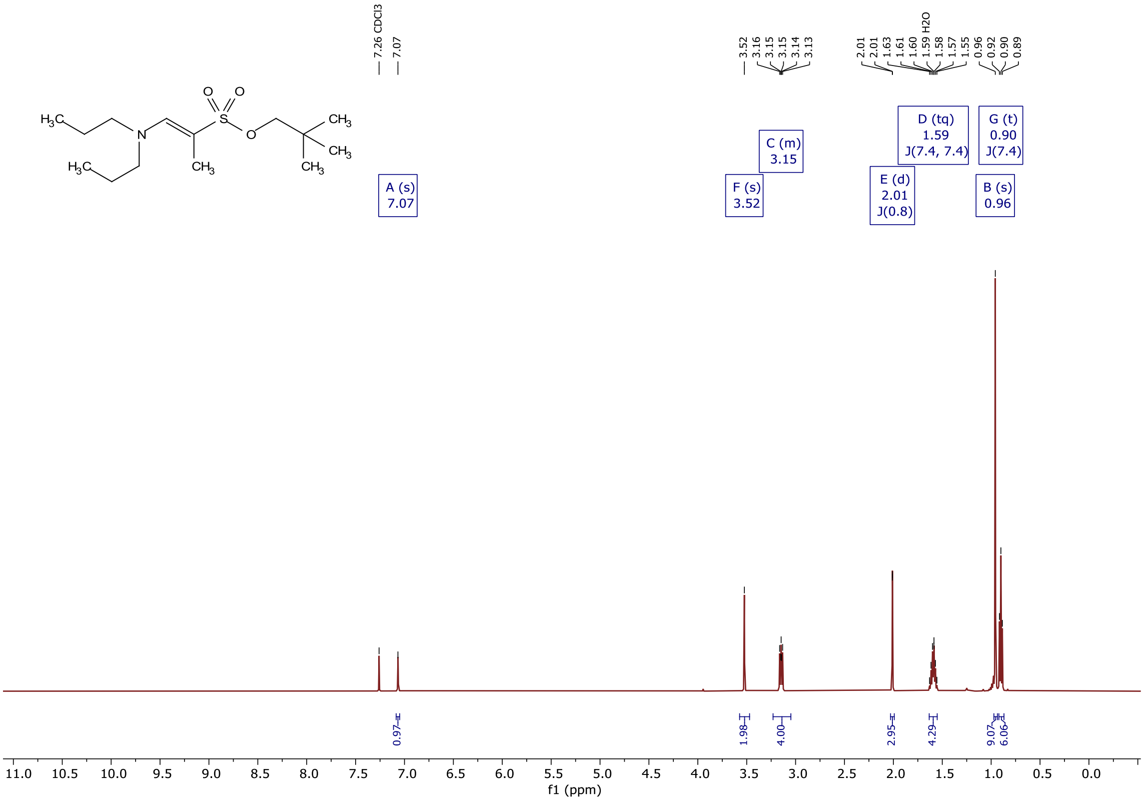

Figure S24: ^{1}H NMR spectrum (500 MHz, $CDCl_3$) of **3f**.

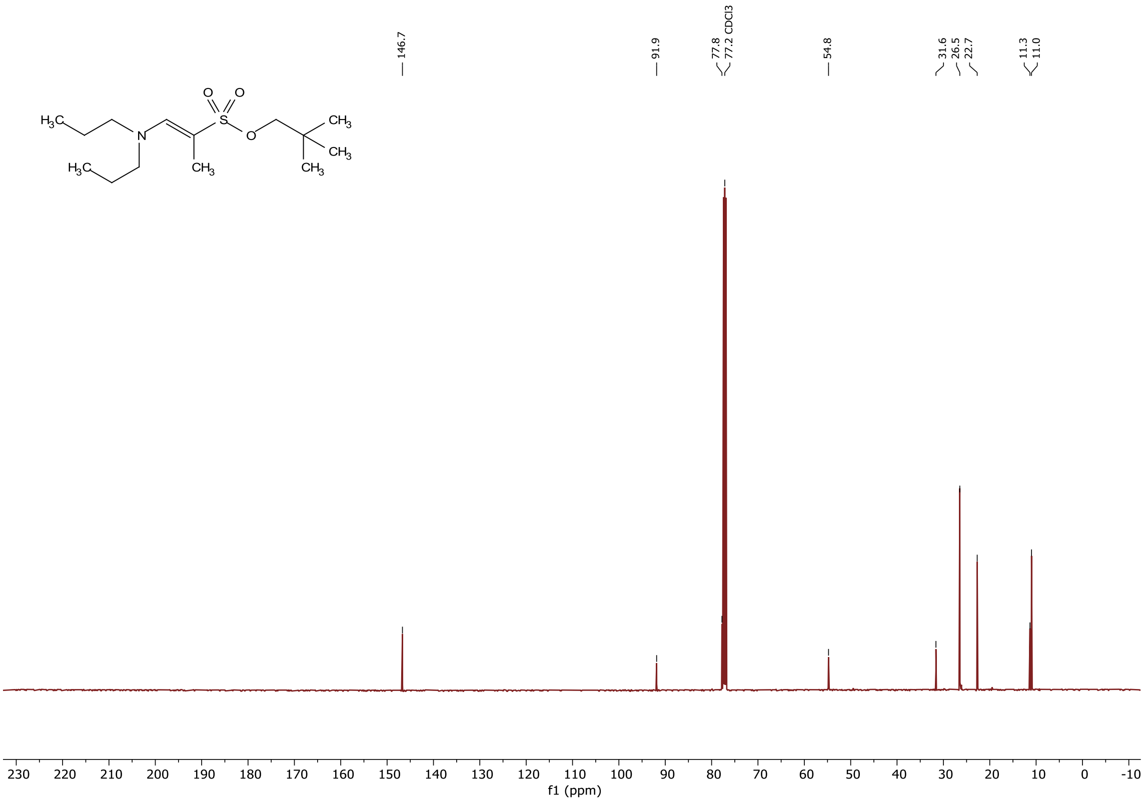

Figure S25: ^{13}C NMR spectrum (126 MHz, $CDCl_3$) of **3f**.

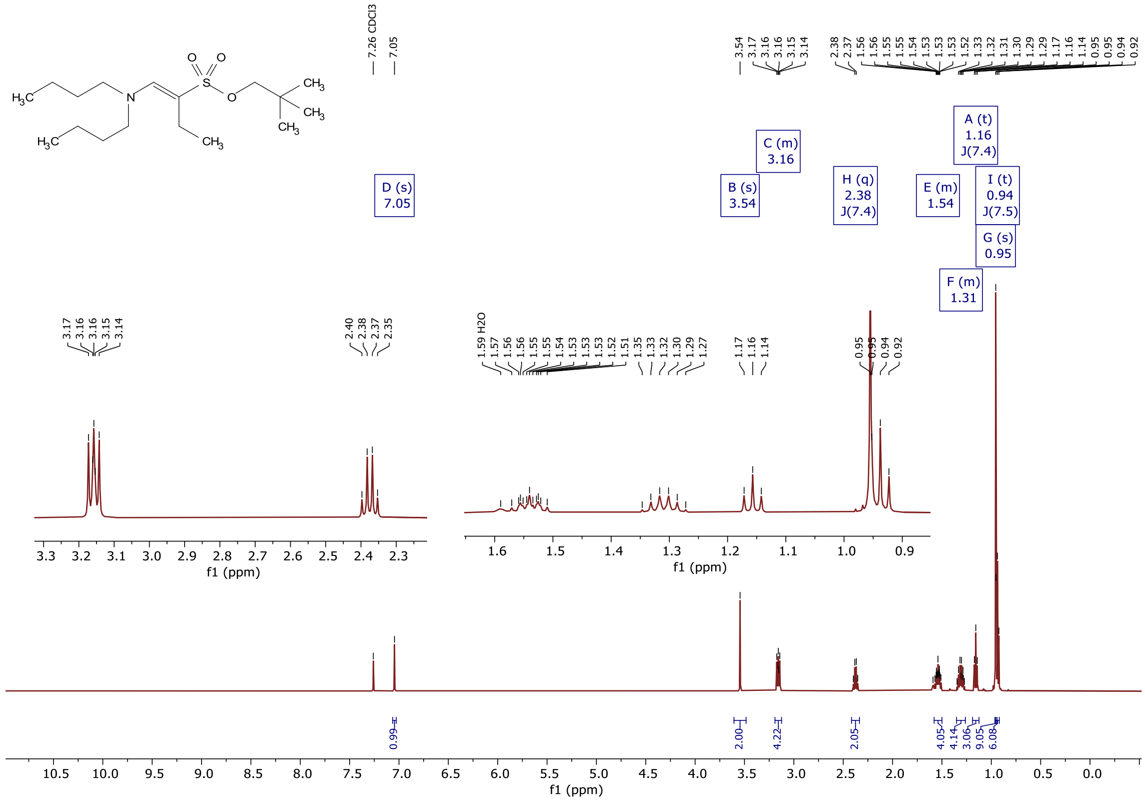

Figure S26: ^{1}H NMR spectrum (500 MHz, $CDCl_3$) of **3g**.

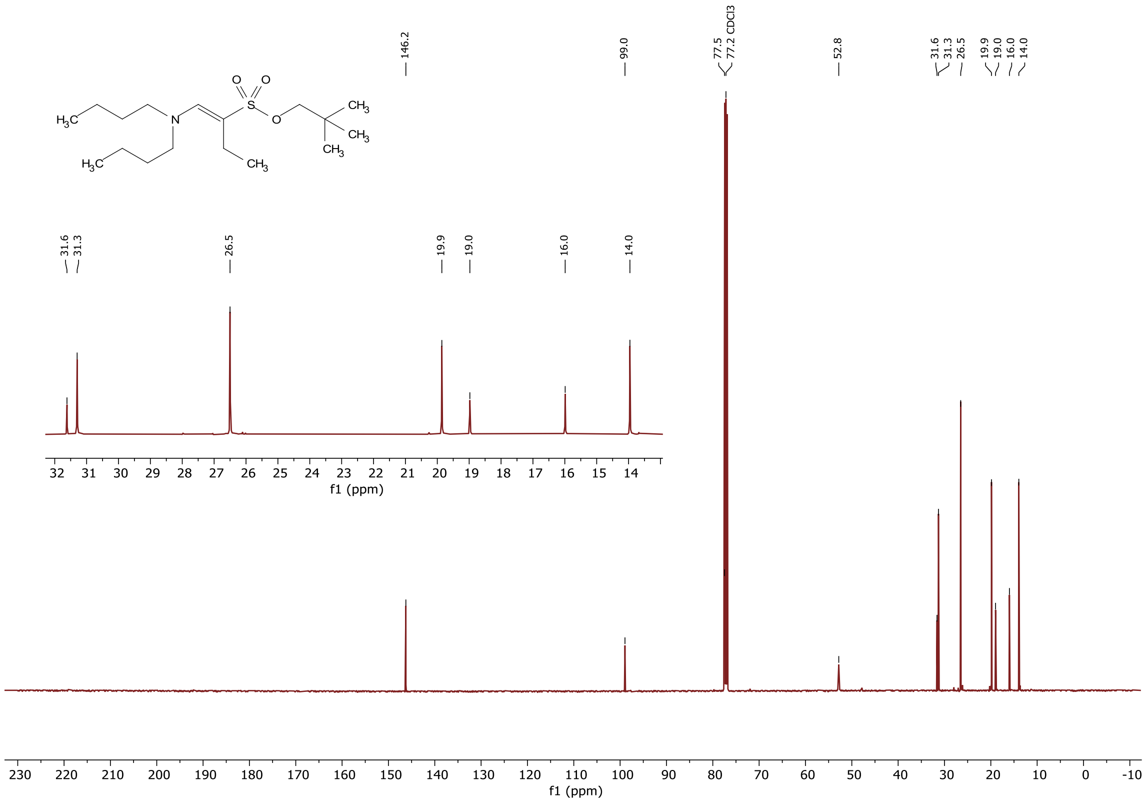

Figure S27: ^{13}C NMR spectrum (126 MHz, $CDCl_3$) of **3g**.

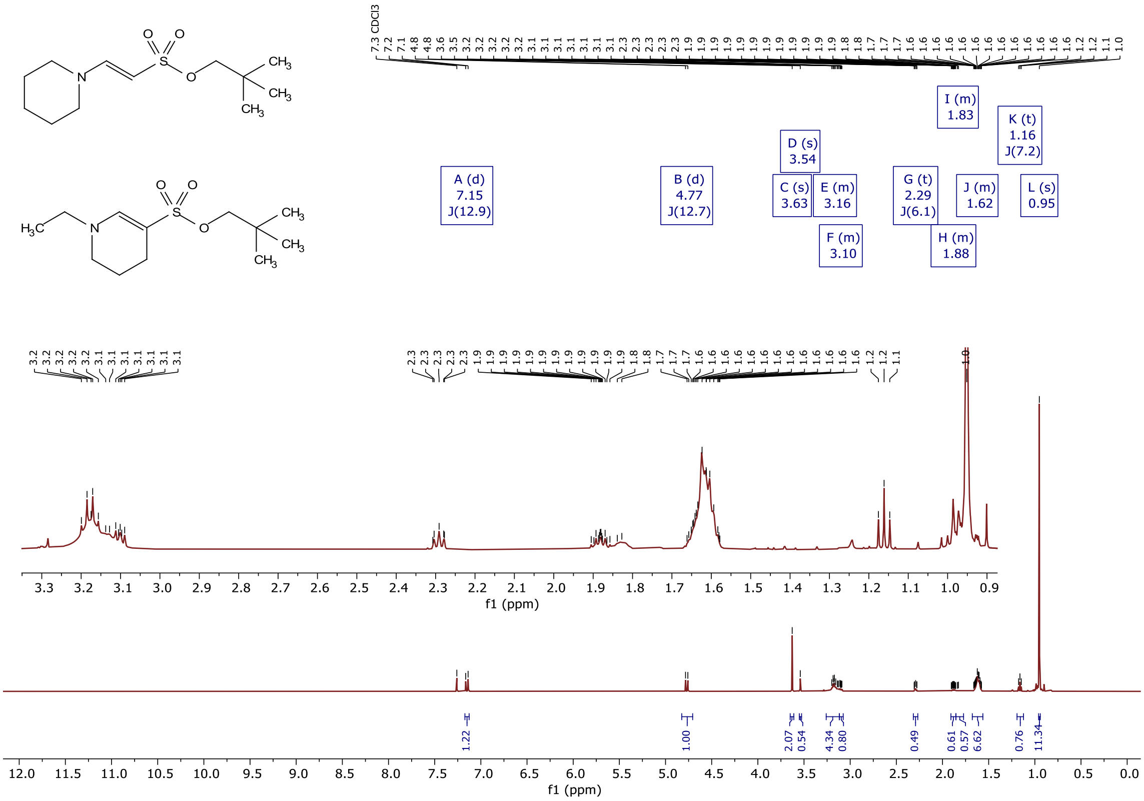

Figure S28: ^{1}H NMR spectrum (500 MHz, $CDCl_3$) of the product mixture of **3h-*exo*** and **3h-*endo***.

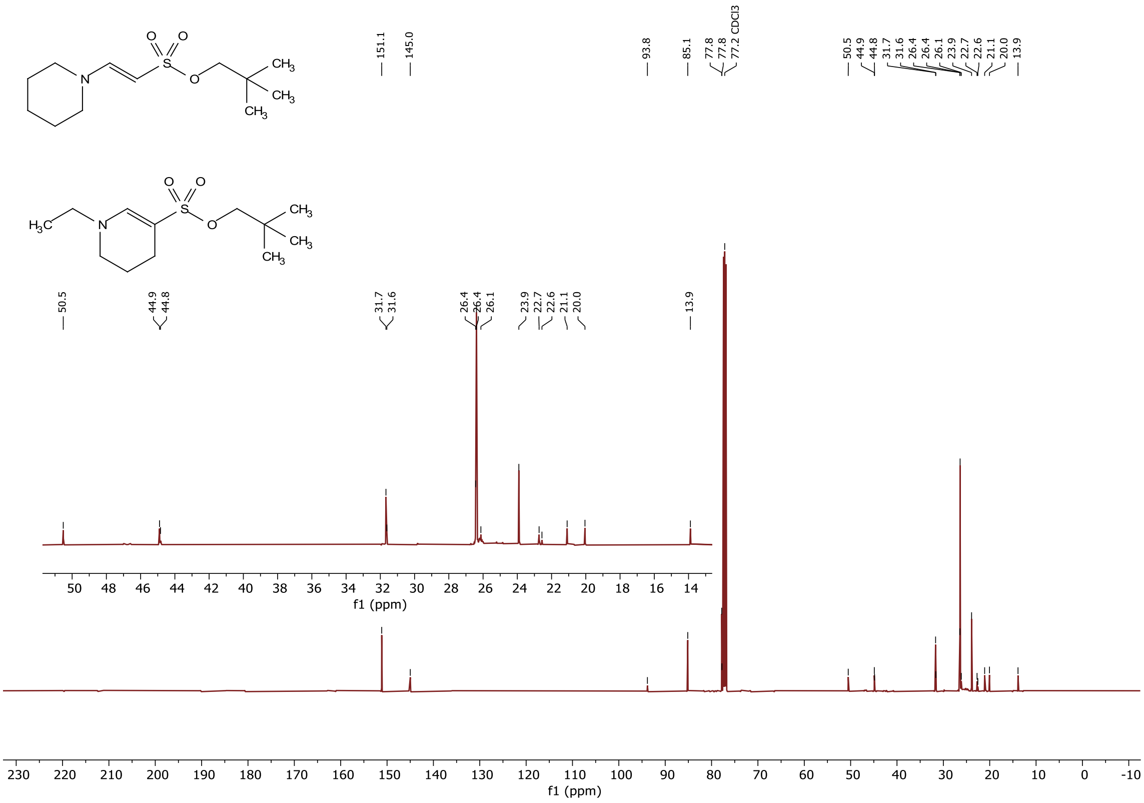

Figure S29: ^{13}C NMR spectrum (126 MHz, $CDCl_3$) of the product mixture of **3h-*exo*** and **3h-*endo***.

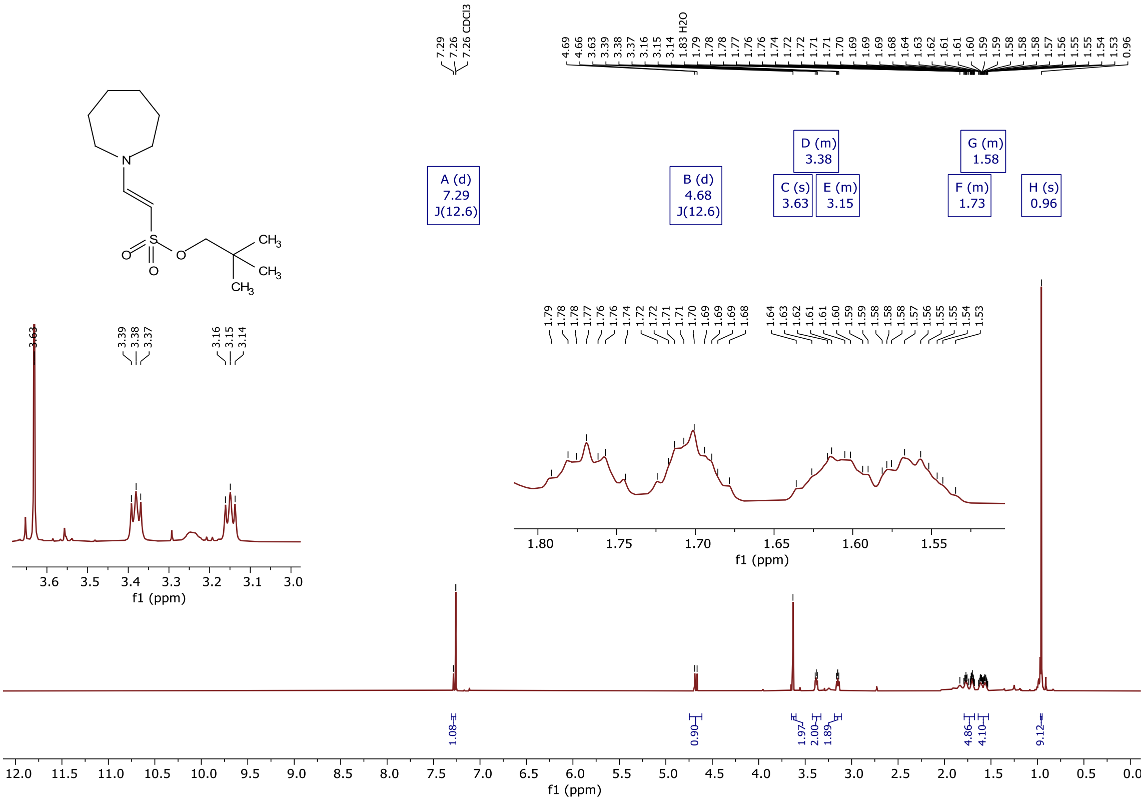

Figure S30: ^{1}H NMR spectrum (500 MHz, $CDCl_3$) of **3i-*exo***.

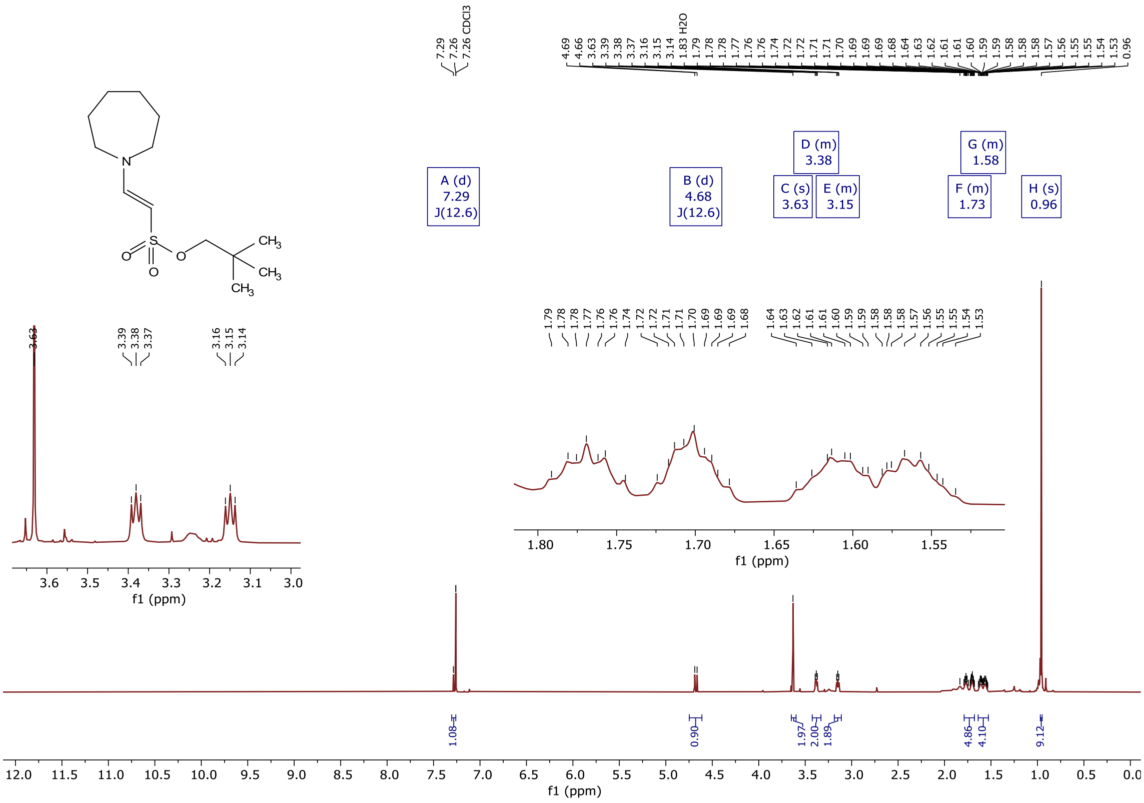

Figure S31: ^{13}C NMR spectrum (126 MHz, $CDCl_3$) of **3i-*exo***.

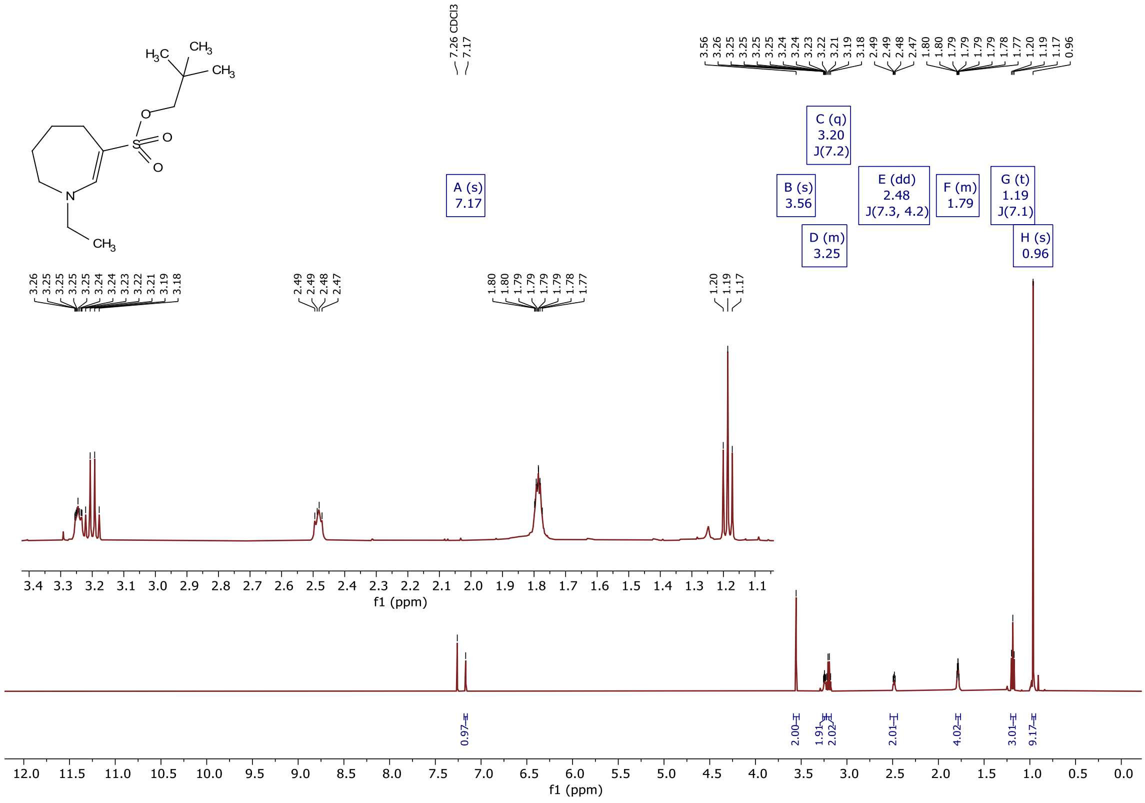

Figure S32: ^{1}H NMR spectrum (500 MHz, $CDCl_3$) of **3i-*endo***.

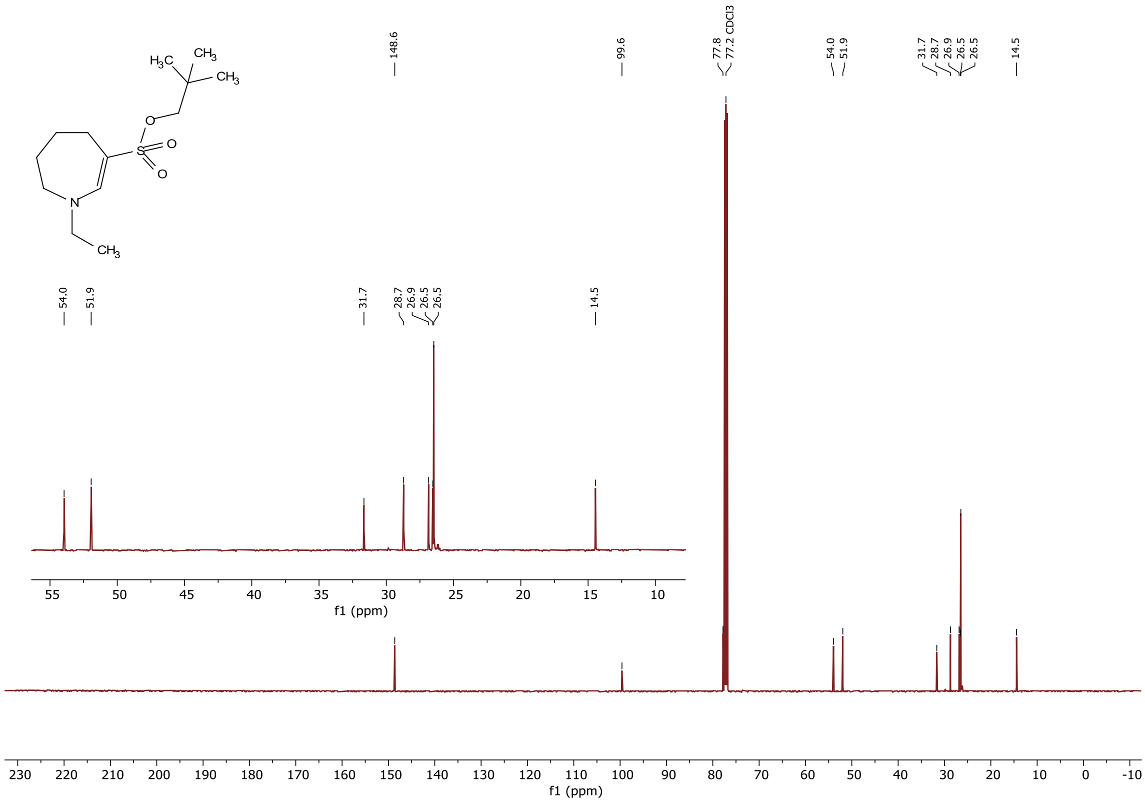

Figure S33: ^{13}C NMR spectrum (126 MHz, $CDCl_3$) of **3i-*endo***.

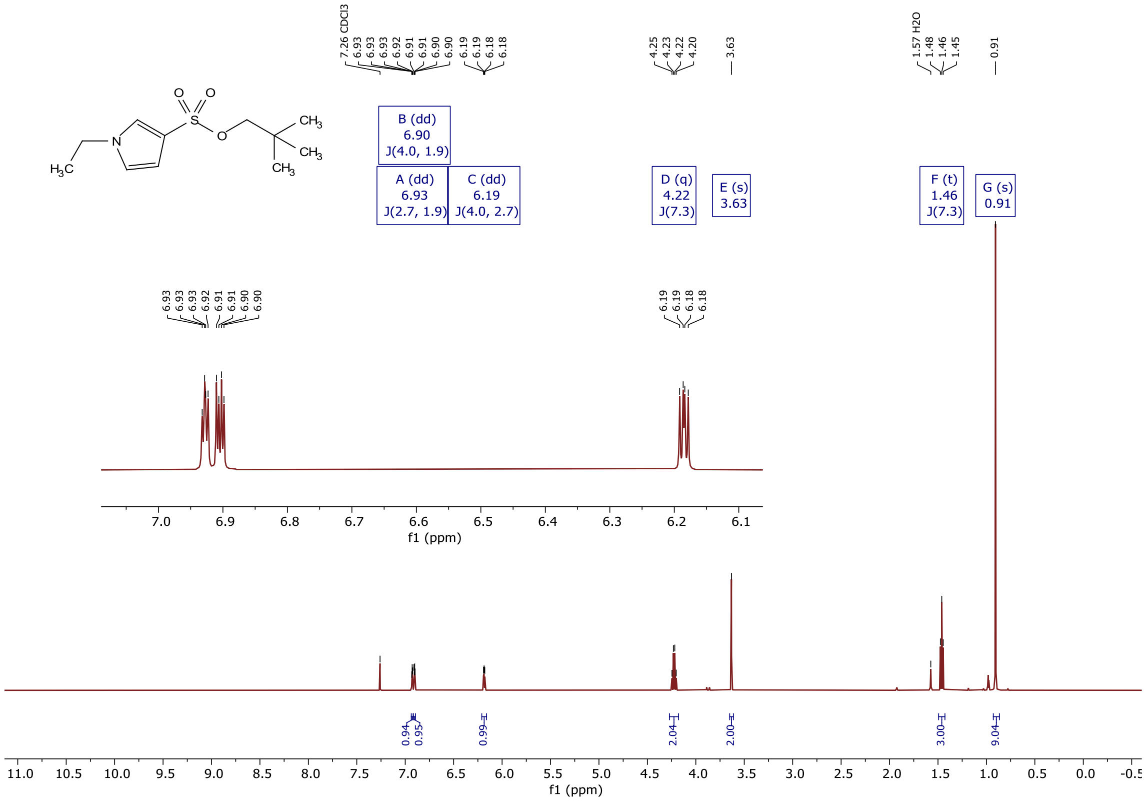

Figure S34: ^{1}H NMR spectrum (500 MHz, $CDCl_3$) of **3j**.

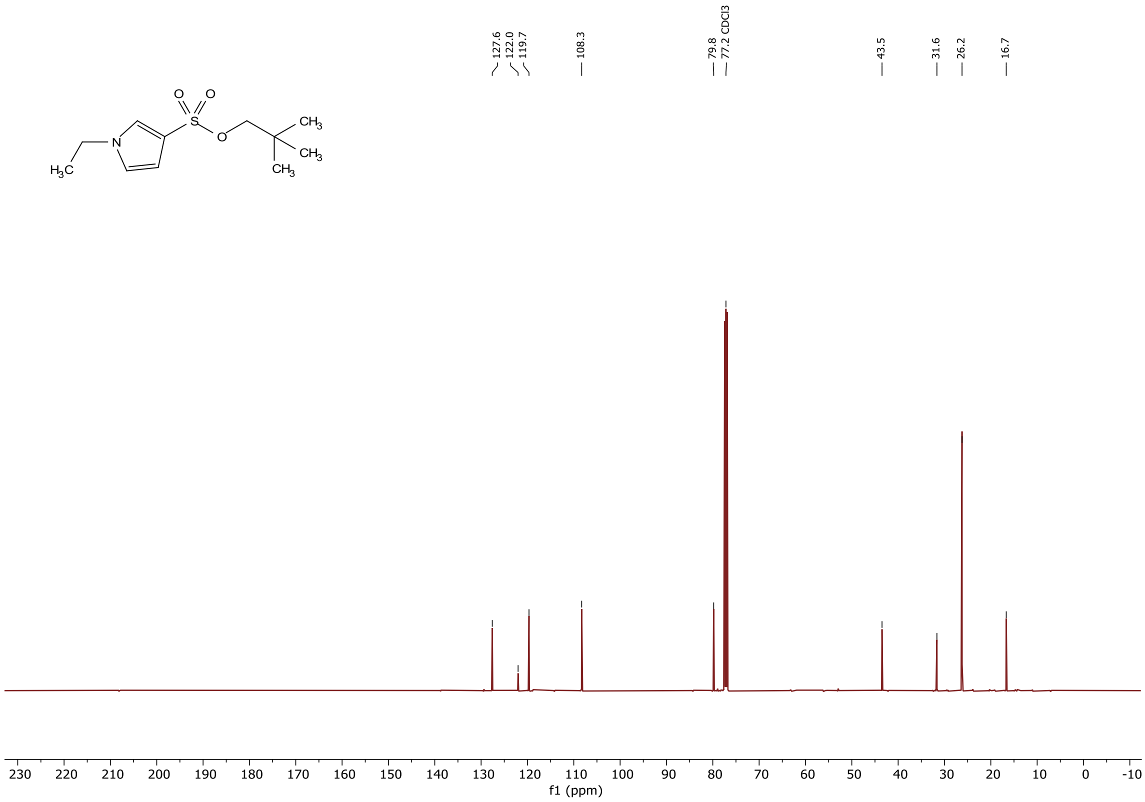

Figure S35: ^{13}C NMR spectrum (126 MHz, $CDCl_3$) of **3j**.

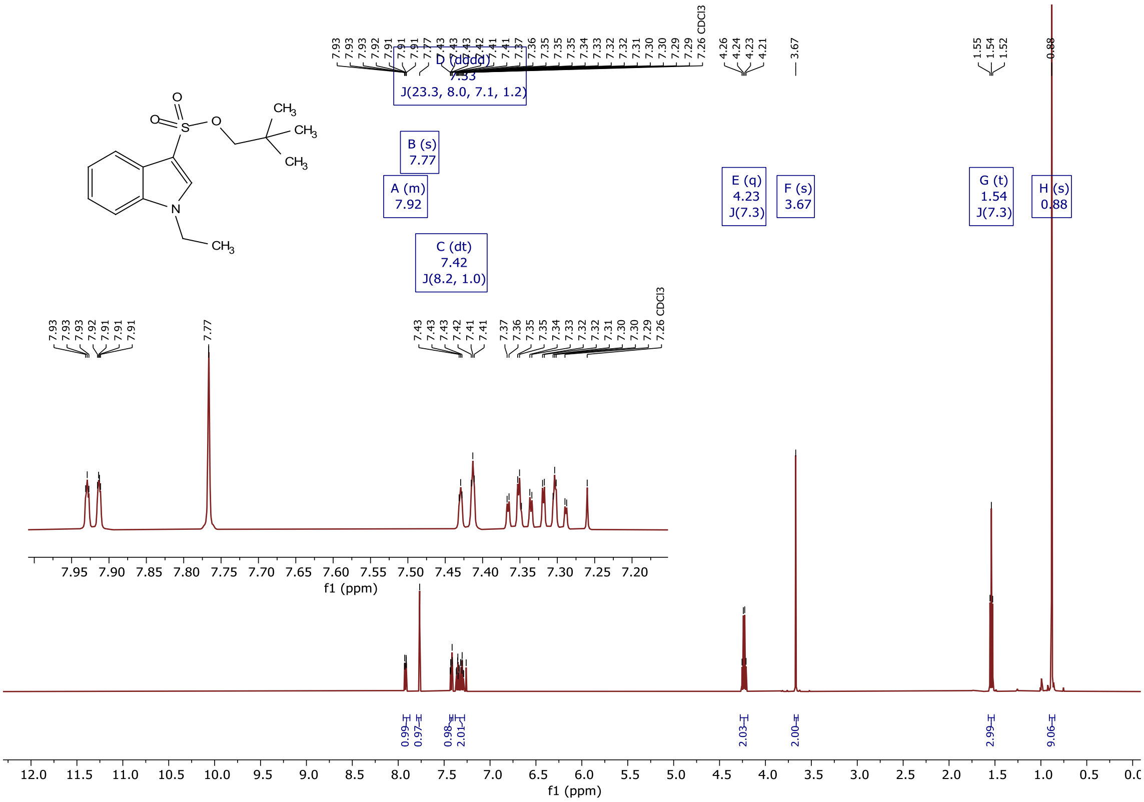

Figure S36: ^{1}H NMR spectrum (500 MHz, $CDCl_3$) of **3k**.

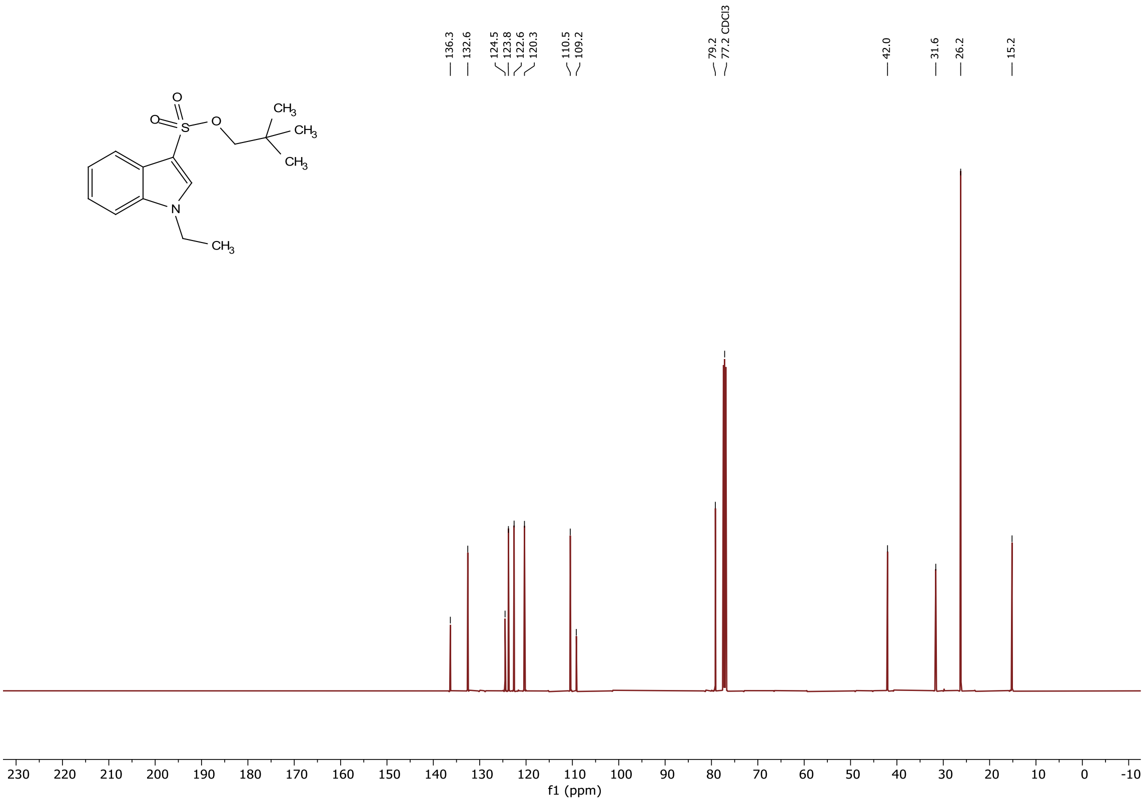

Figure S37: ^{13}C NMR spectrum (126 MHz, $CDCl_3$) of **3k**.

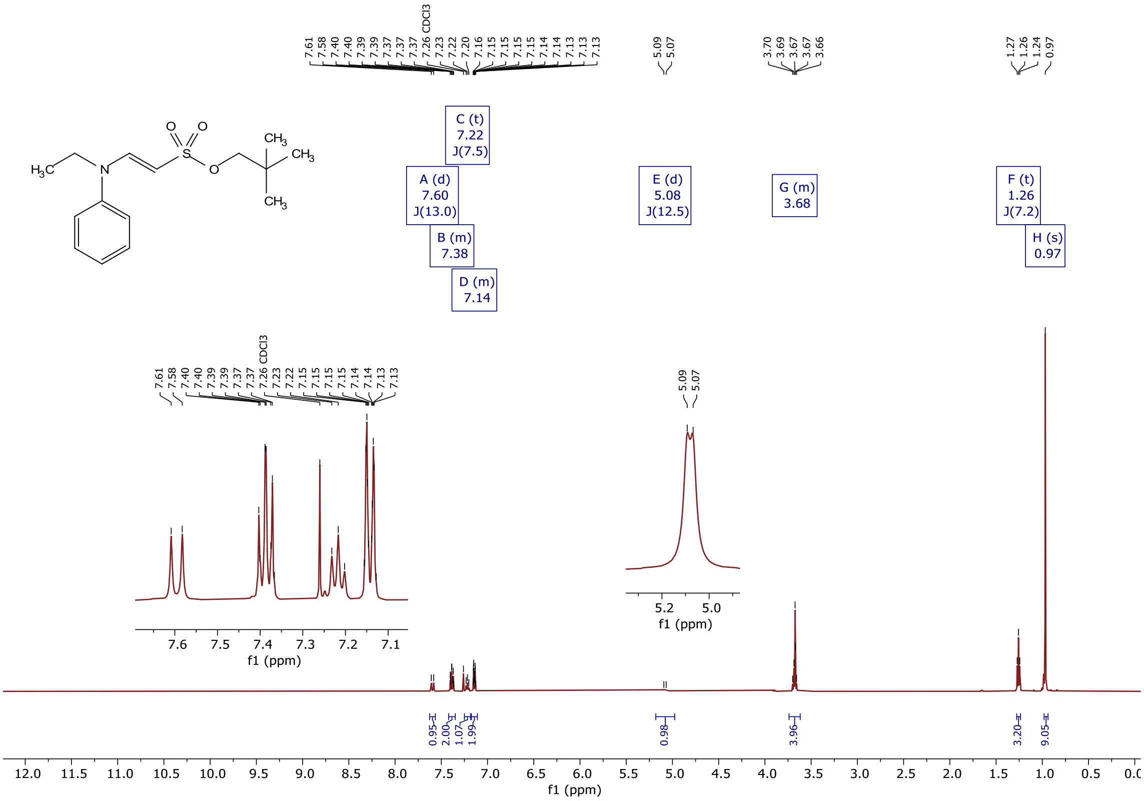

Figure S38: ^{1}H NMR spectrum (500 MHz, $CDCl_3$) of **3l**.

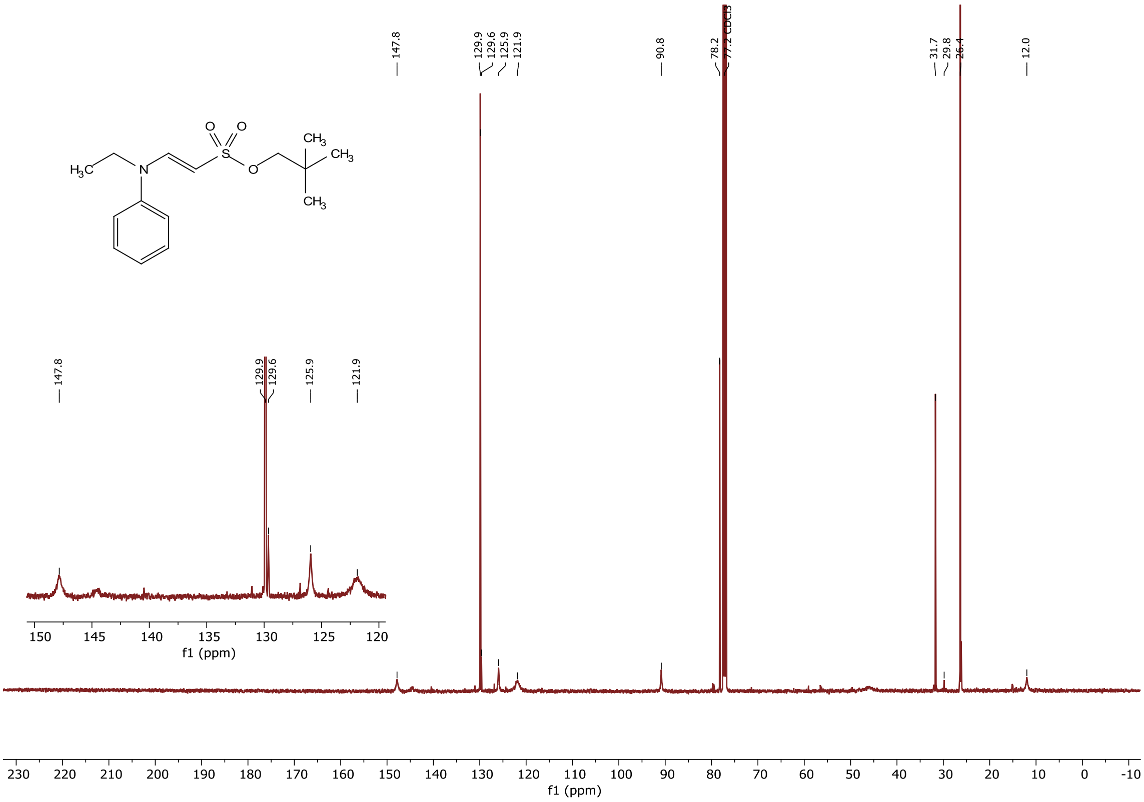

Figure S39: ^{13}C NMR spectrum (126 MHz, $CDCl_3$) of **3l**.

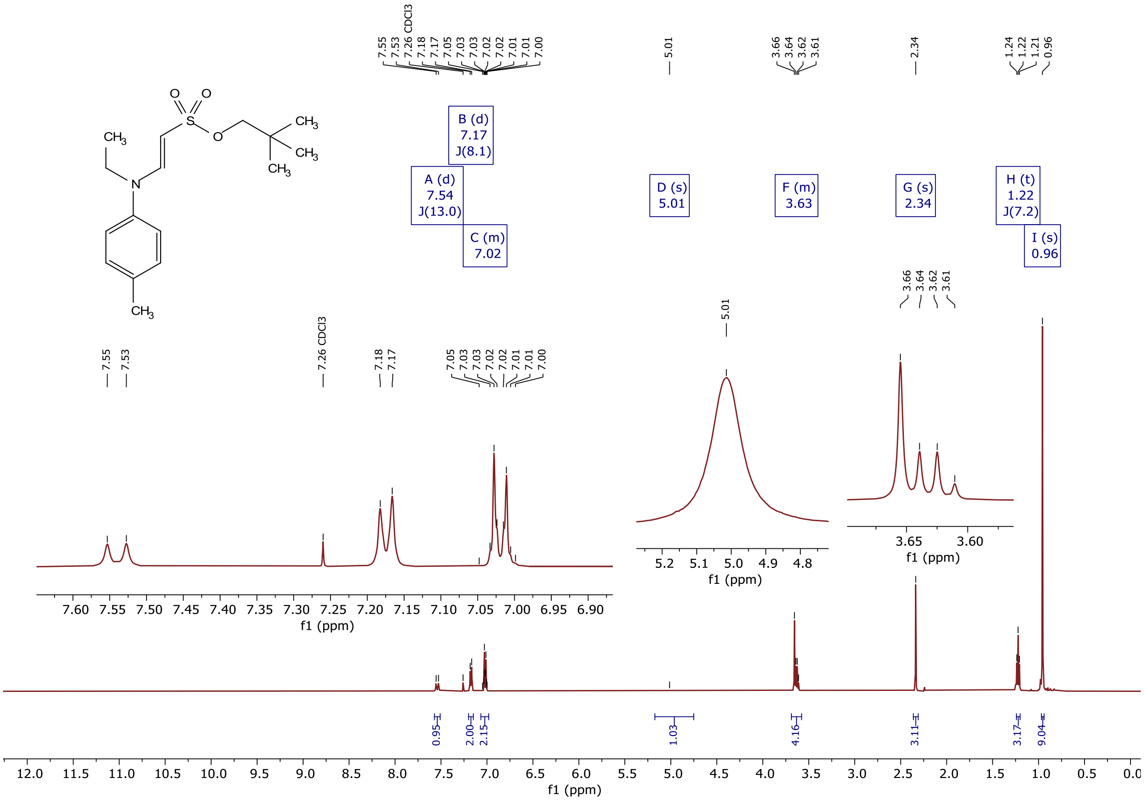

Figure S40: ^{1}H NMR spectrum (500 MHz, $CDCl_3$) of **3m**.

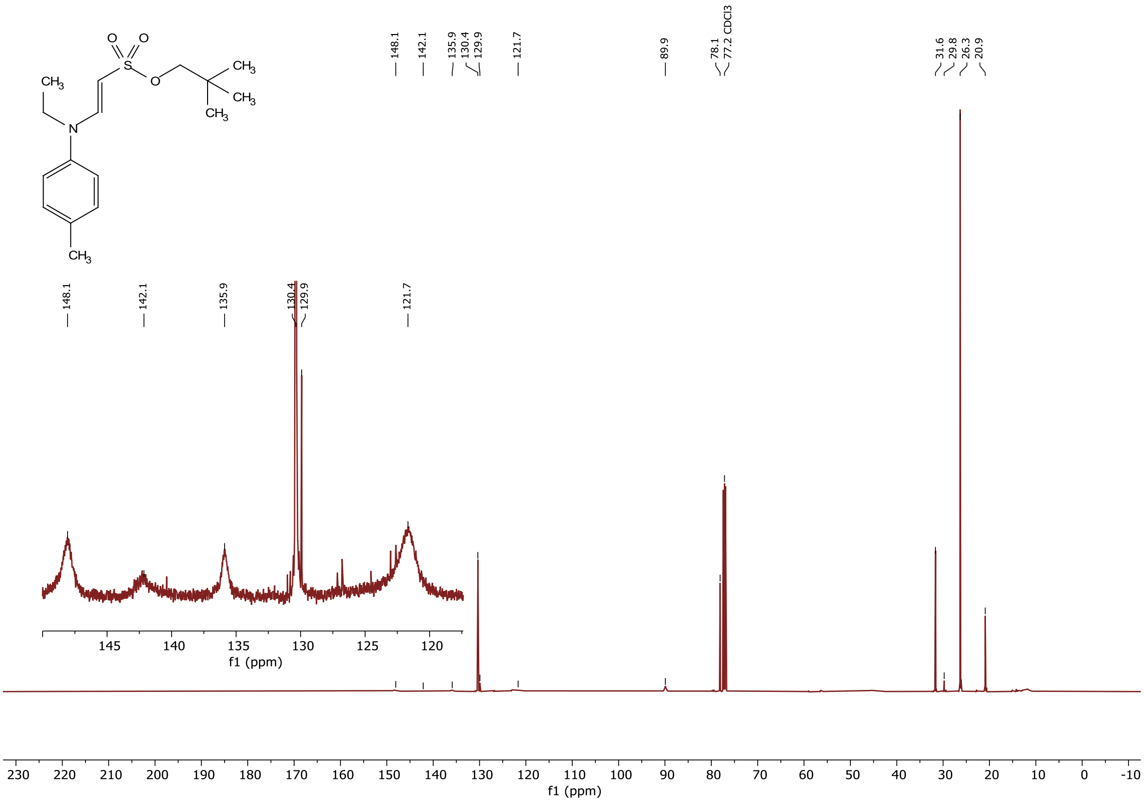

Figure S41: ^{13}C NMR spectrum (126 MHz, $CDCl_3$) of **3m**.

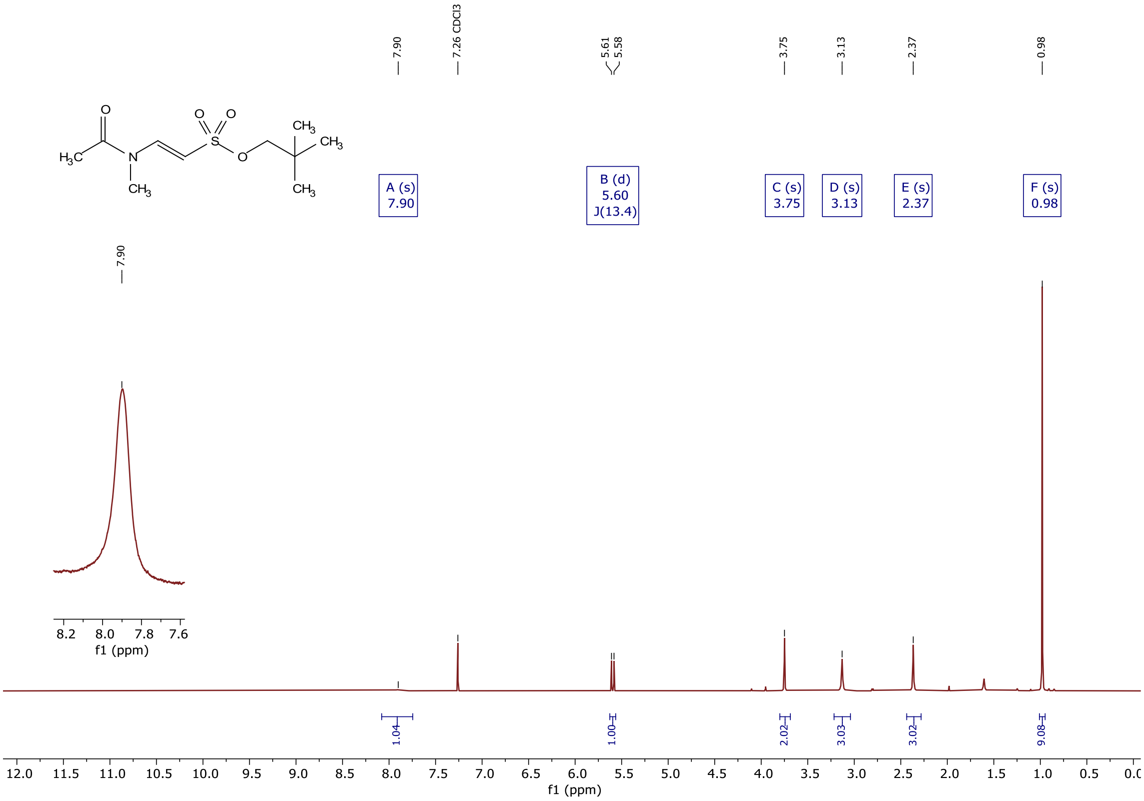

Figure S42: ^{1}H NMR spectrum (500 MHz, $CDCl_3$) of **3n**.

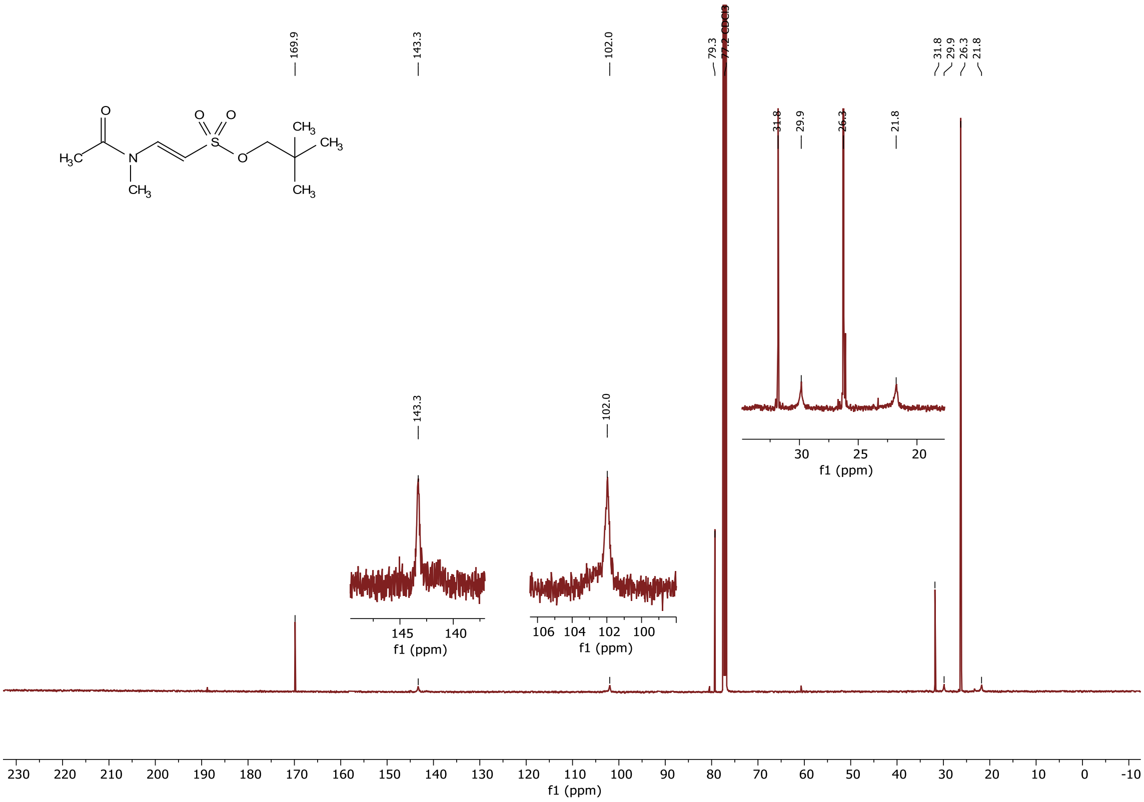

Figure S43: ^{13}C NMR spectrum (126 MHz, $CDCl_3$) of **3n**.

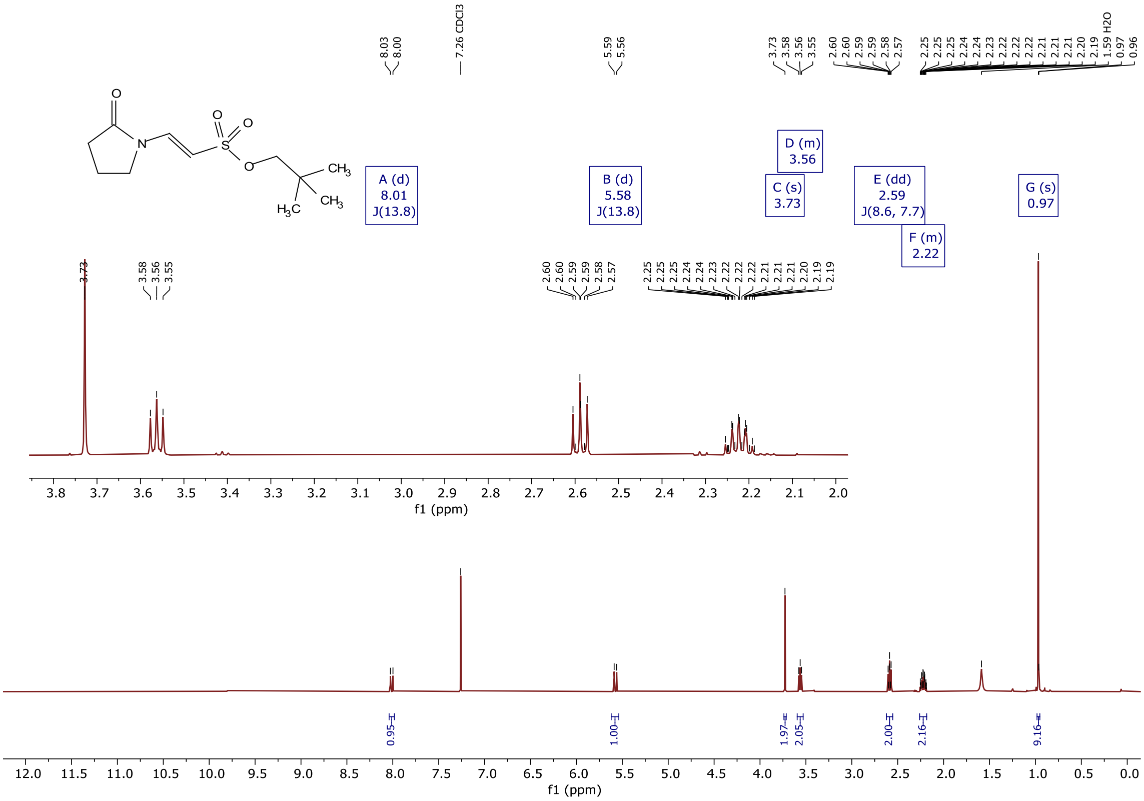

Figure S44: ^{1}H NMR spectrum (500 MHz, $CDCl_3$) of **3o**.

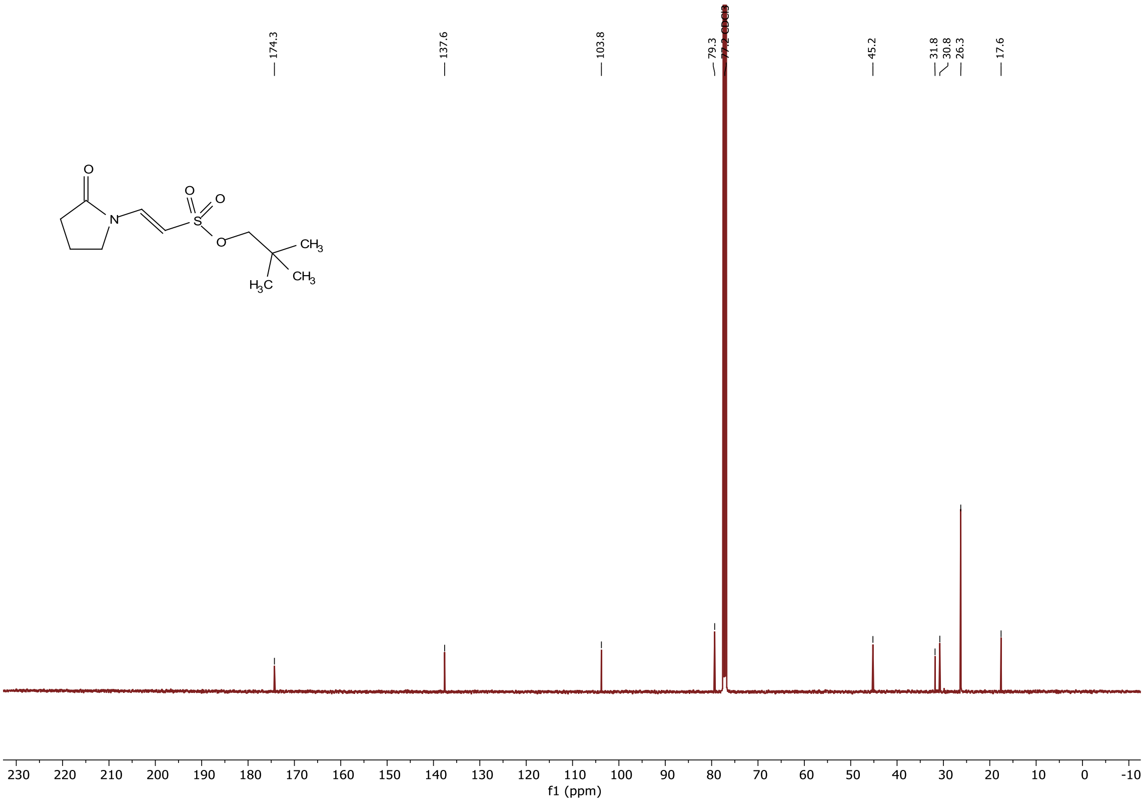

Figure S45: ^{13}C NMR spectrum (126 MHz, $CDCl_3$) of **3o**.

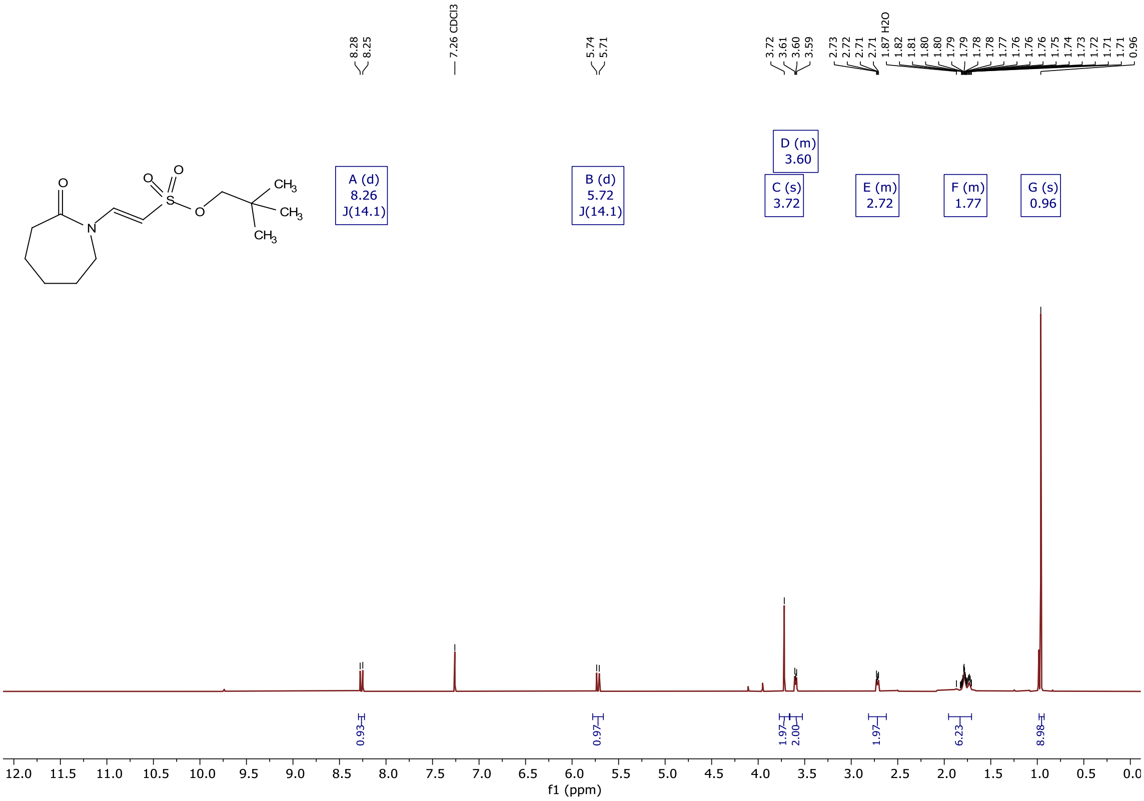

Figure S46: ^{1}H NMR spectrum (500 MHz, $CDCl_3$) of **3p**.

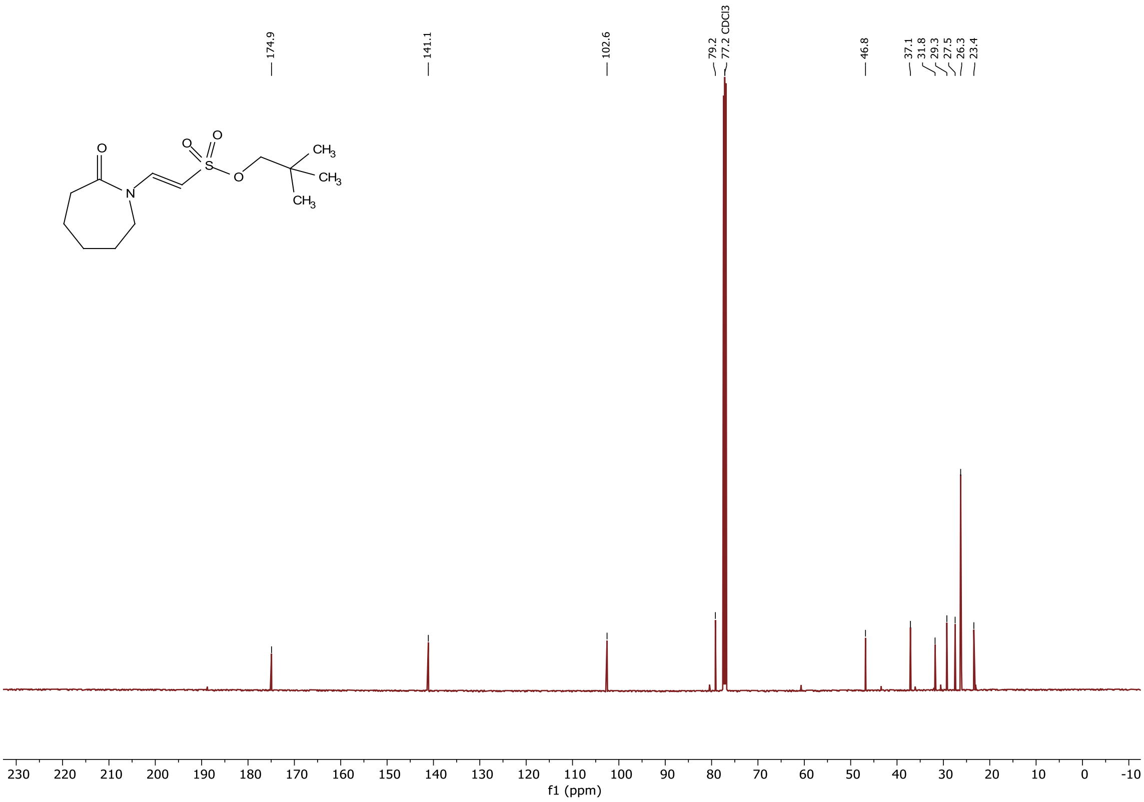

Figure S47: ^{13}C NMR spectrum (126 MHz, $CDCl_3$) of **3p**.

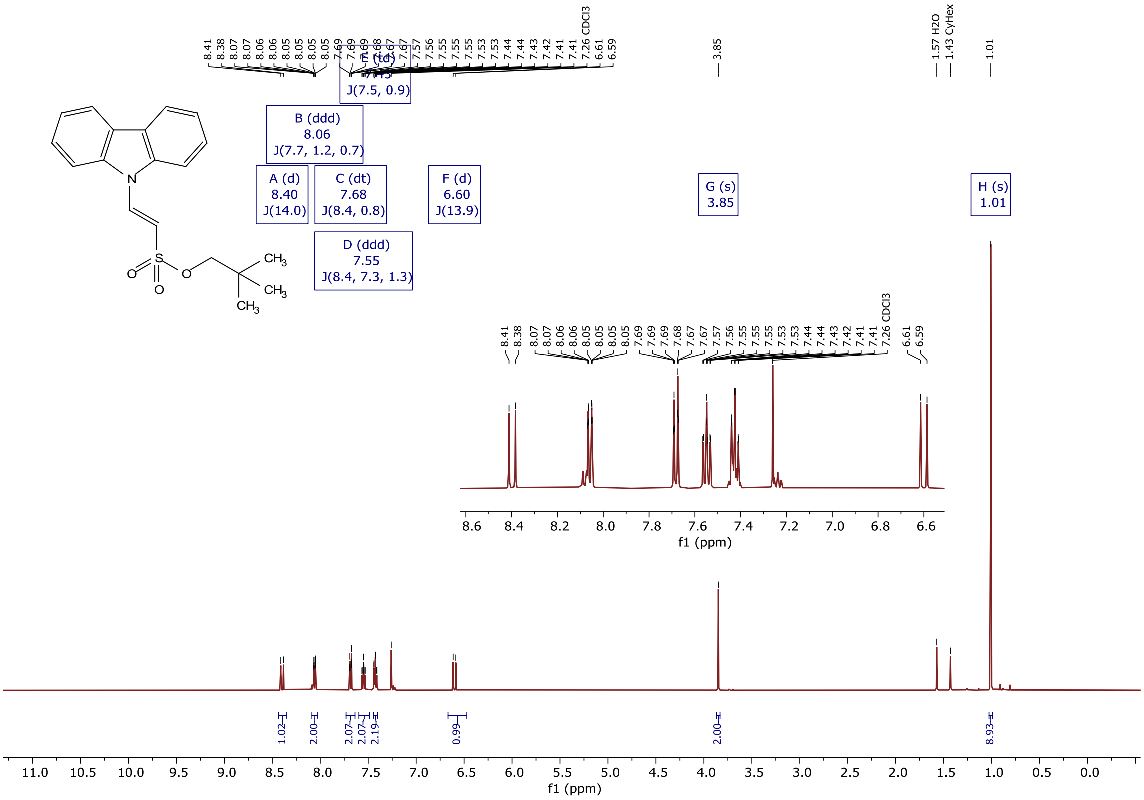

Figure S48: ^1H NMR spectrum (500 MHz, $CDCl_3$) of **3q**.

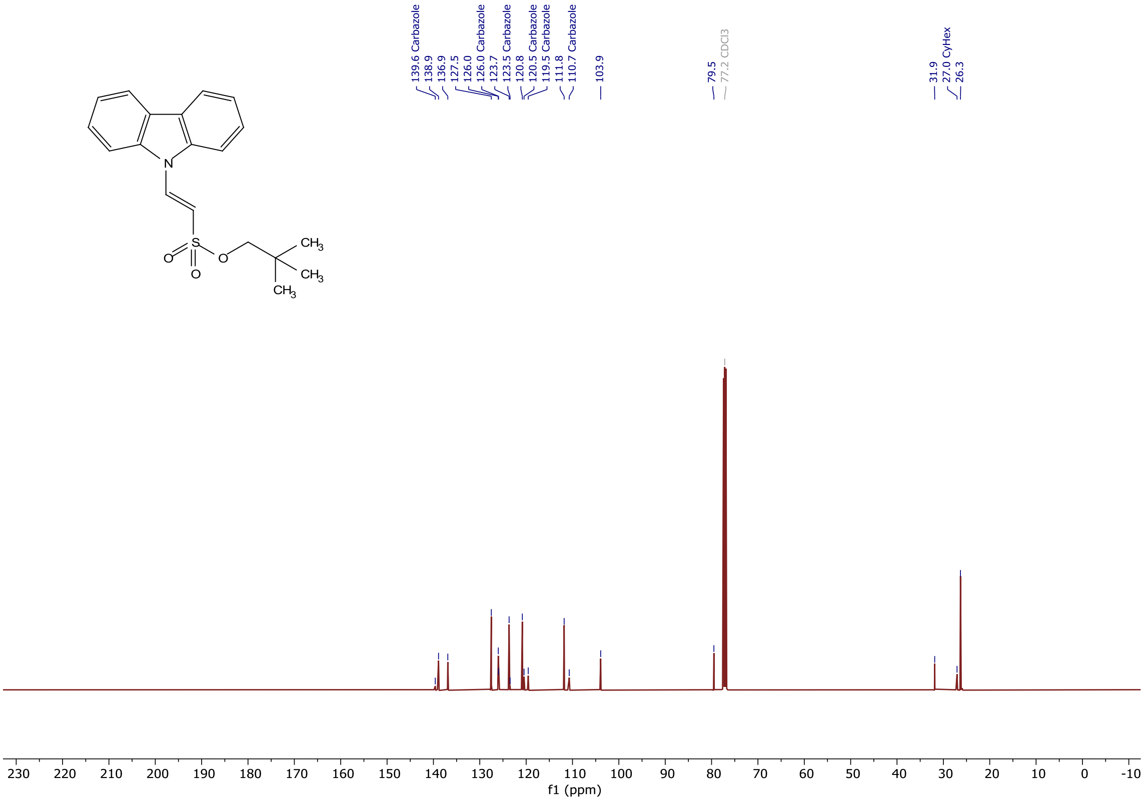

Figure S49: ^{13}C NMR spectrum (126 MHz, $CDCl_3$) of **3q**.

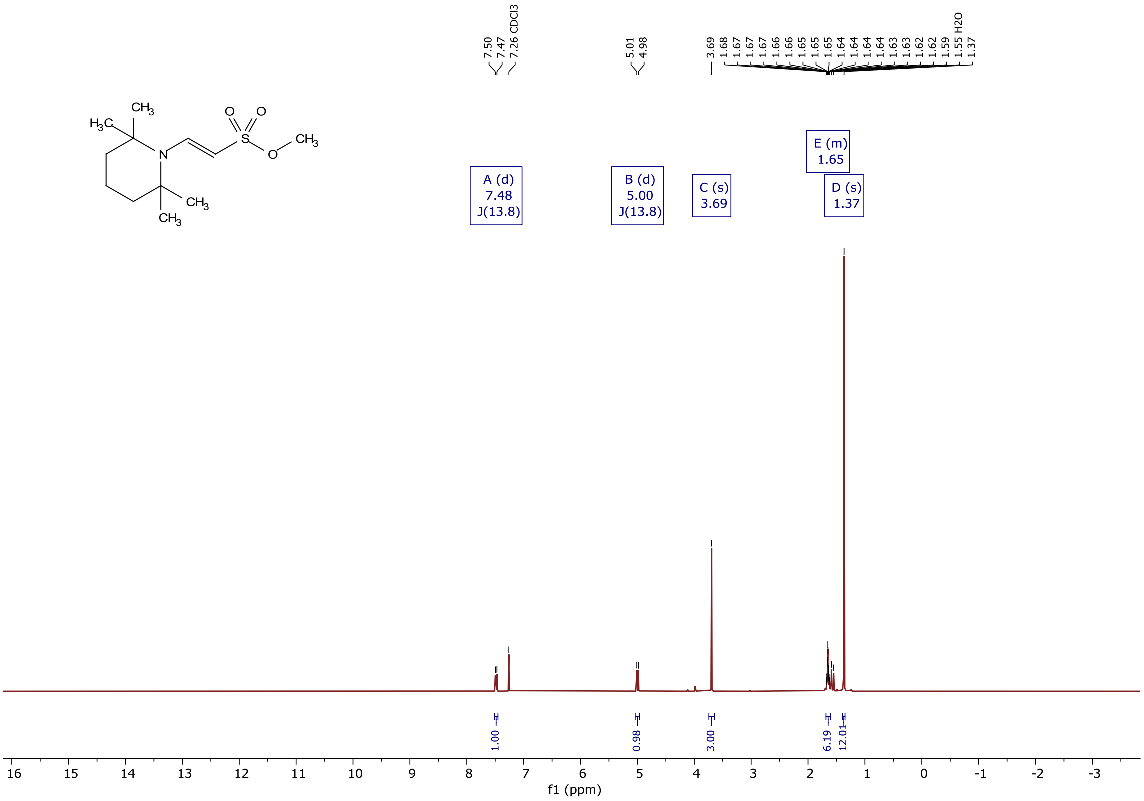

Figure S50: ^{1}H NMR spectrum (500 MHz, $CDCl_3$) of **4a**.

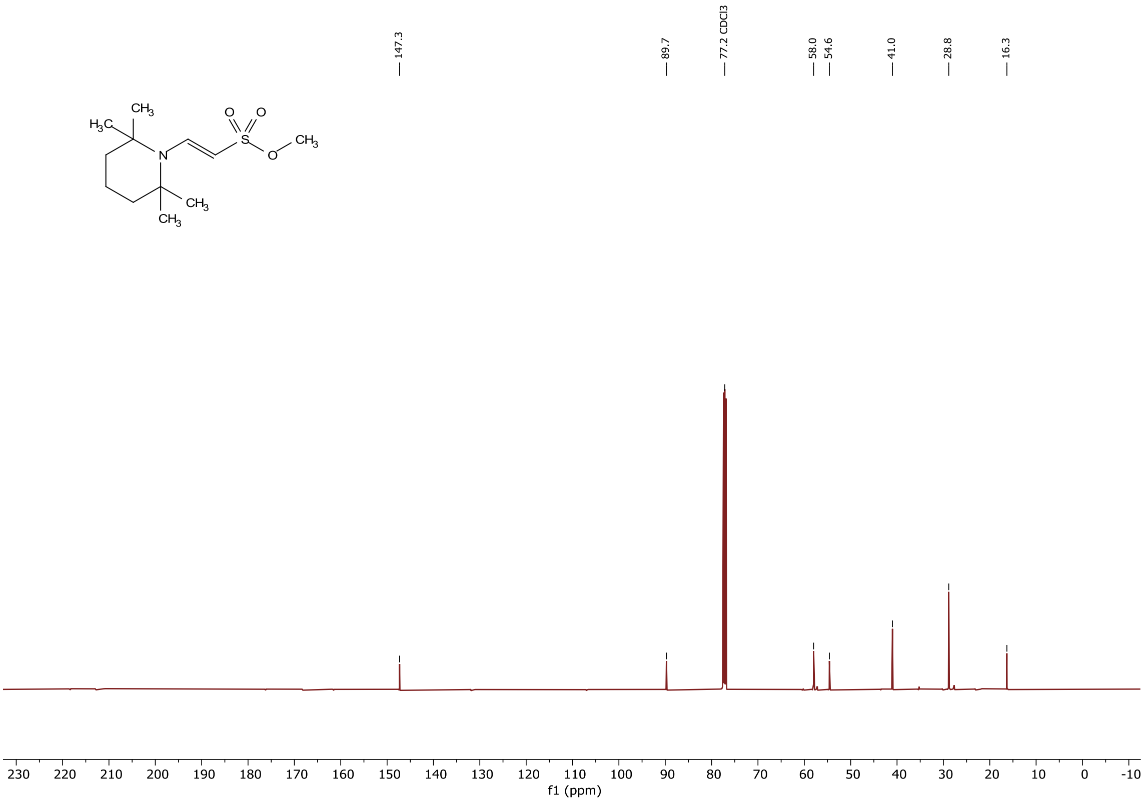

Figure S51: ^{13}C NMR spectrum (126 MHz, $CDCl_3$) of **4a**.

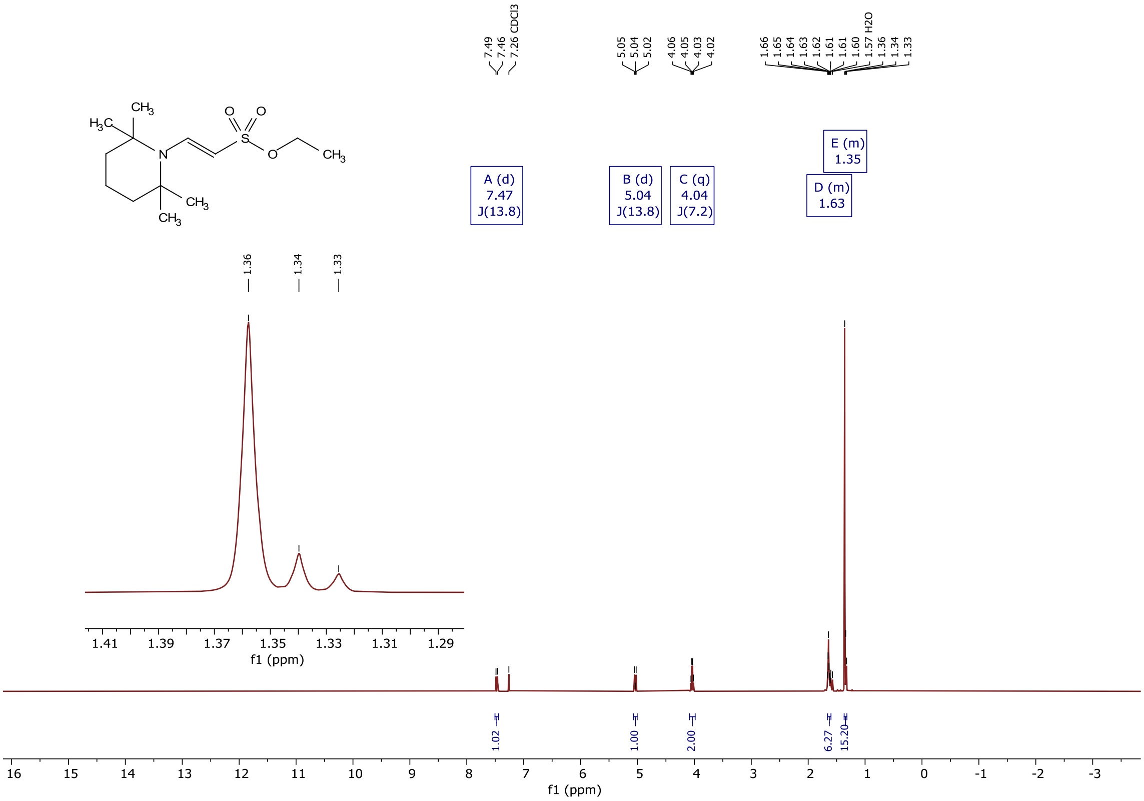

Figure S52: ^{1}H NMR spectrum (500 MHz, $CDCl_3$) of **4b**.

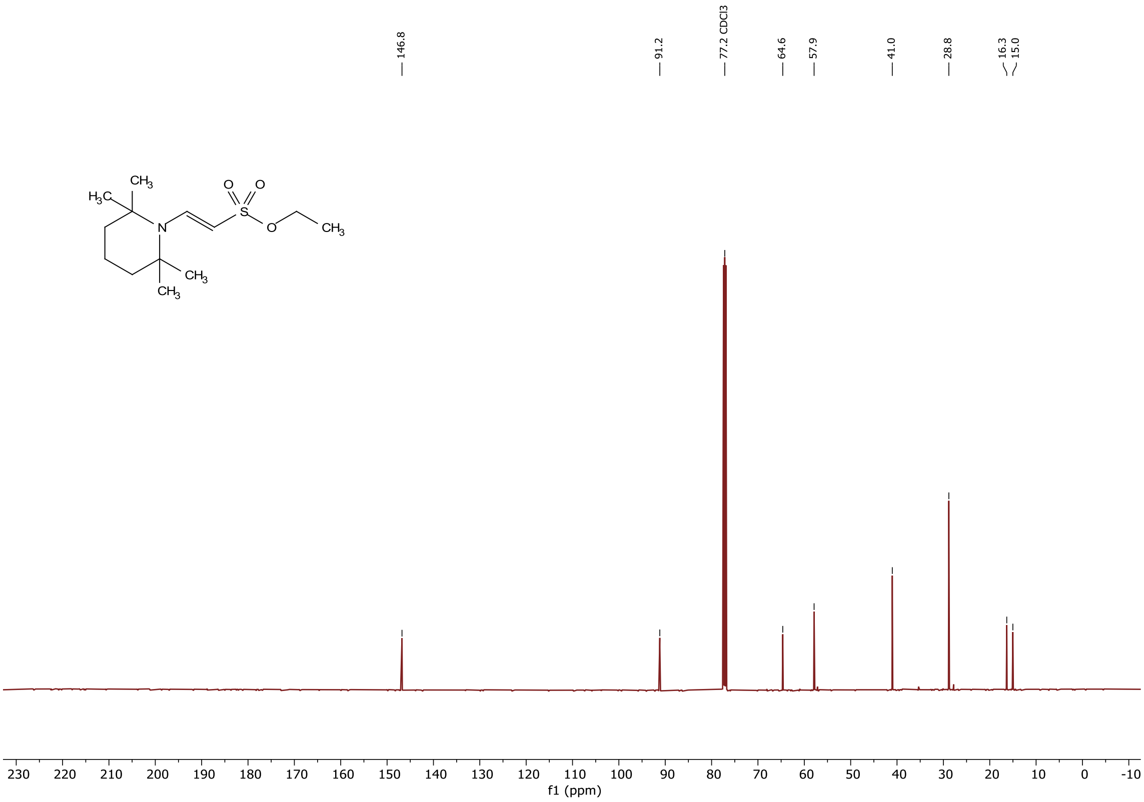

Figure S53: ^{13}C NMR spectrum (126 MHz, $CDCl_3$) of **4b**.

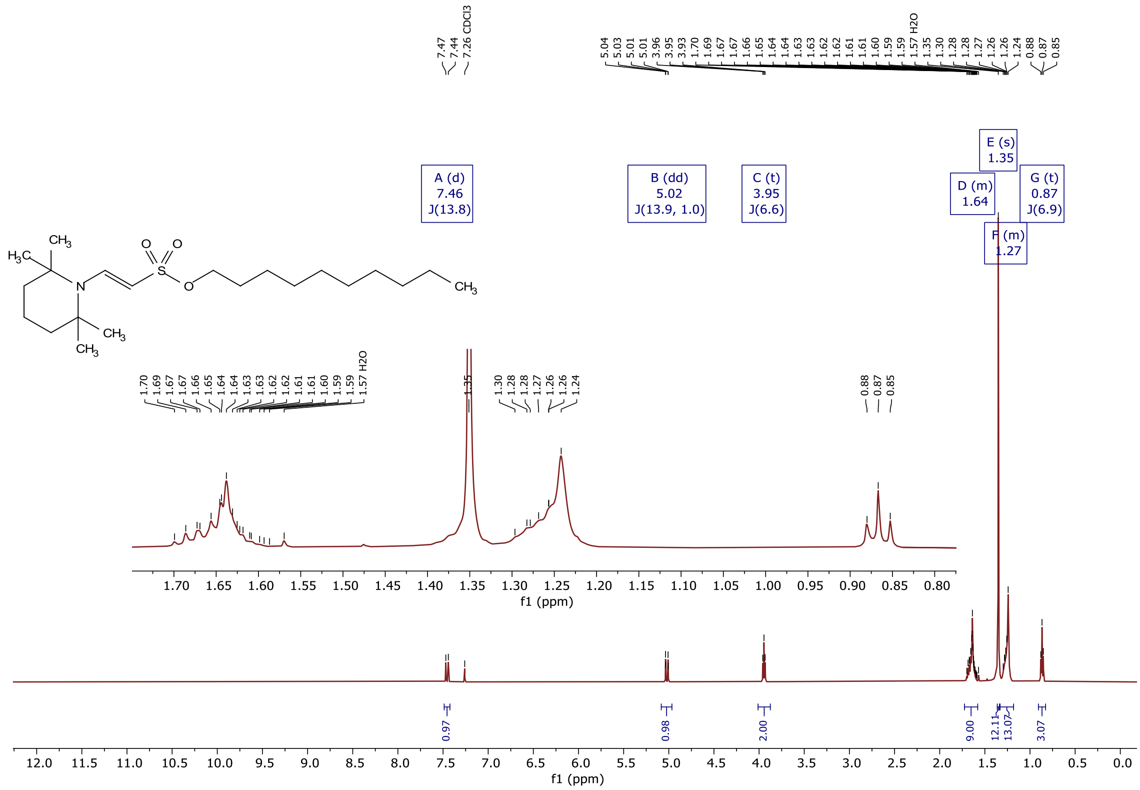

Figure S54: ^{1}H NMR spectrum (500 MHz, $CDCl_3$) of **4c**.

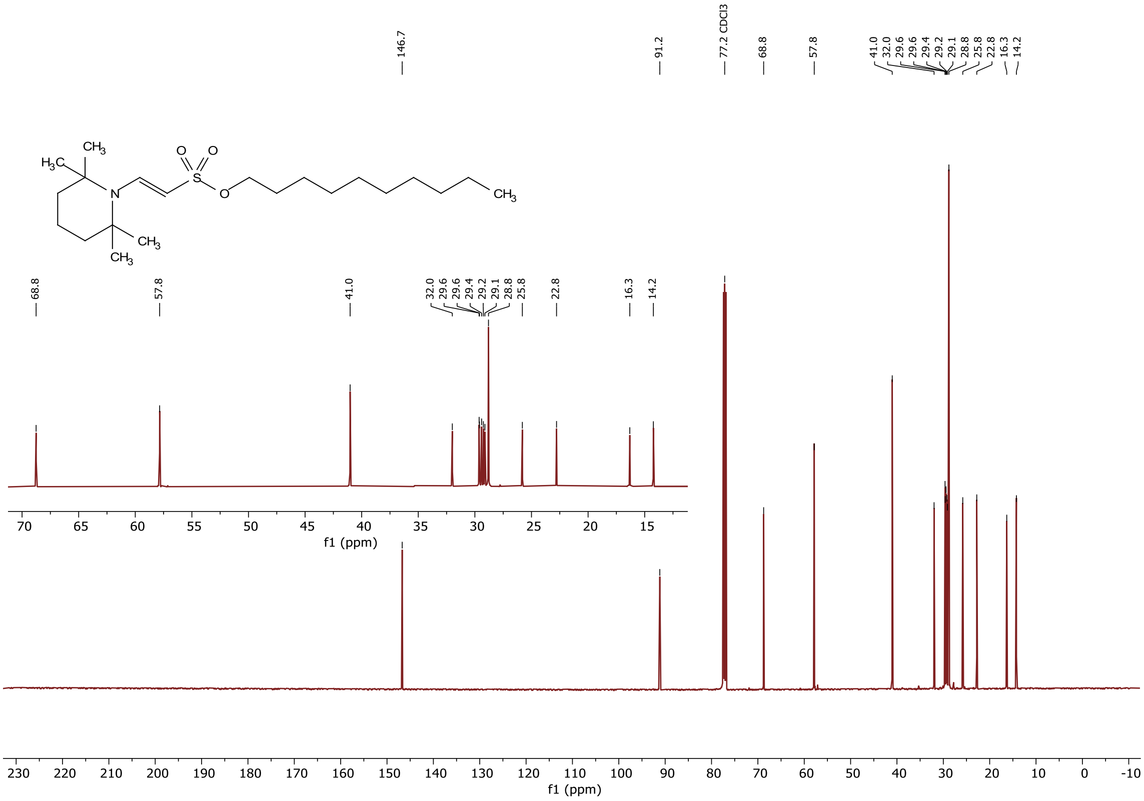

Figure S55: ^{13}C NMR spectrum (126 MHz, $CDCl_3$) of **4c**.

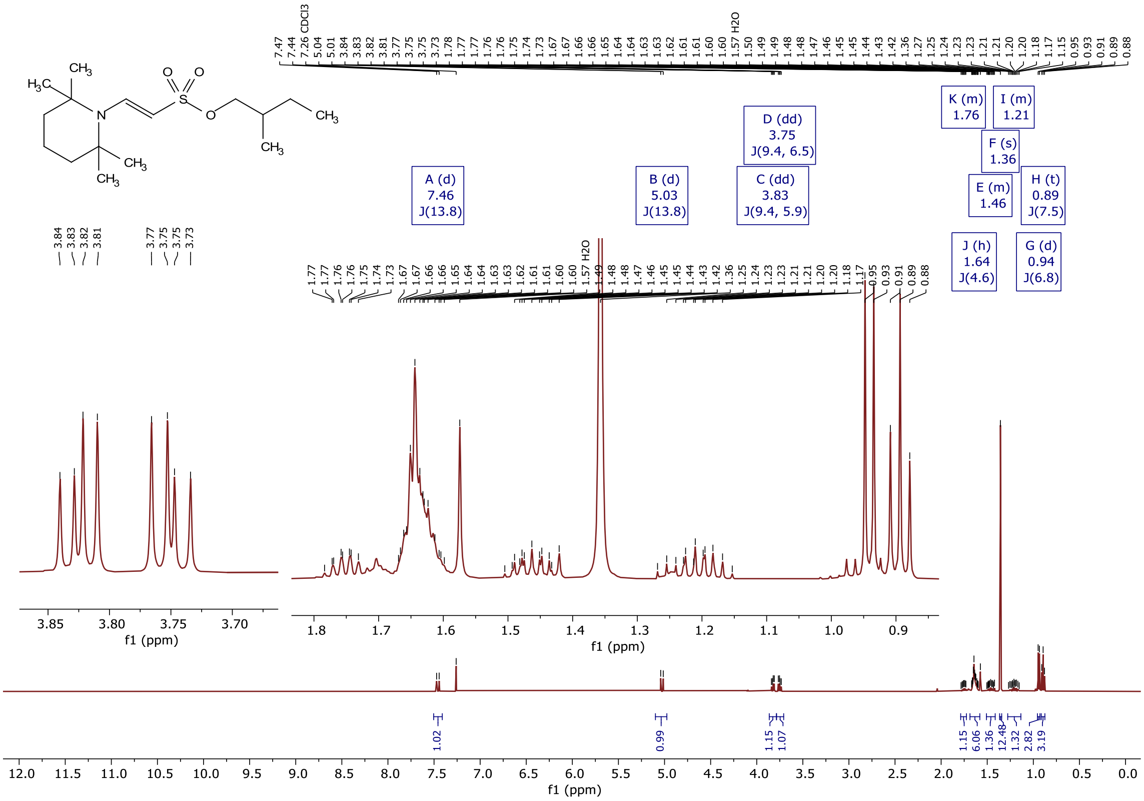

Figure S56: ^{1}H NMR spectrum (500 MHz, $CDCl_3$) of **4d**.

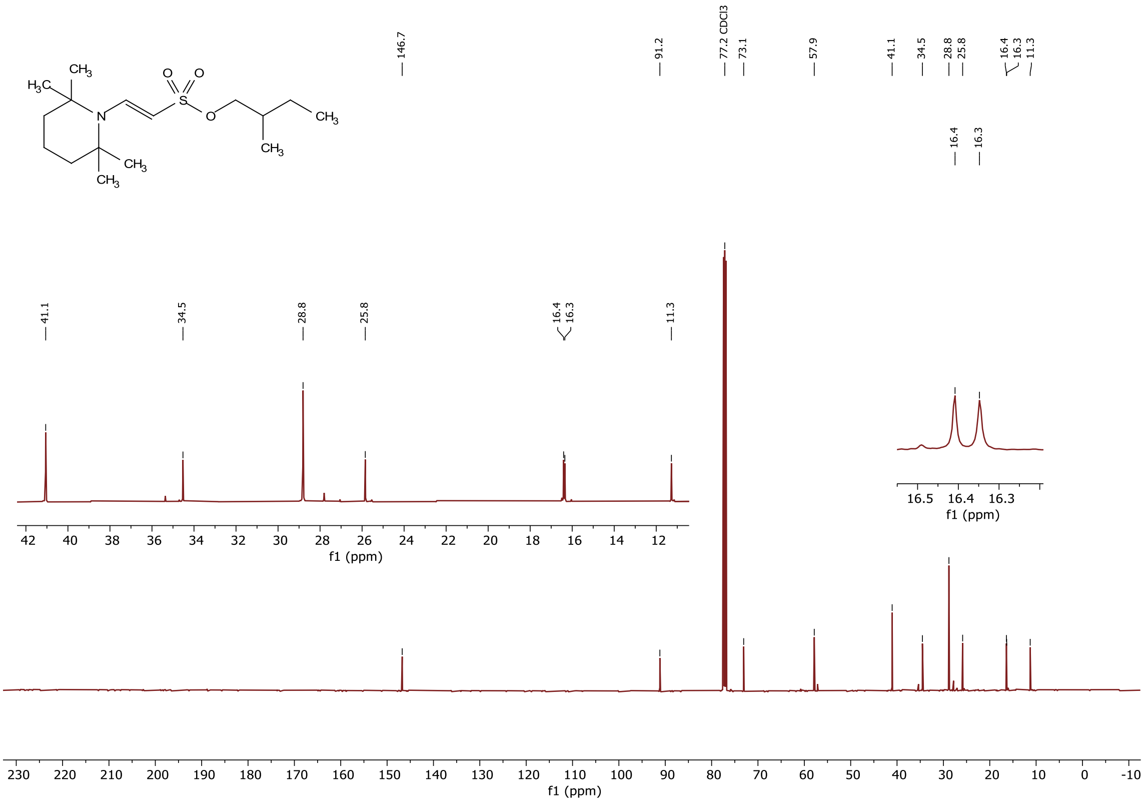

Figure S57: ^{13}C NMR spectrum (126 MHz, $CDCl_3$) of **4d**.

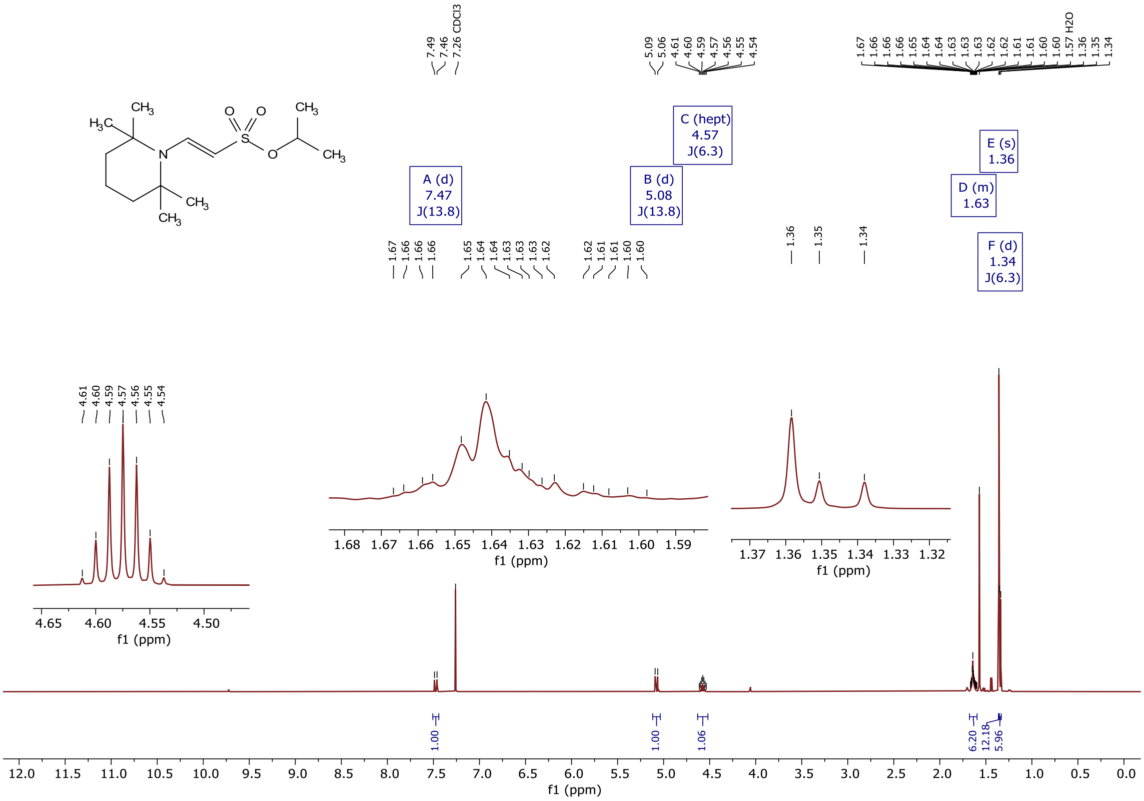

Figure S58: ^{1}H NMR spectrum (500 MHz, $CDCl_3$) of **4e**.

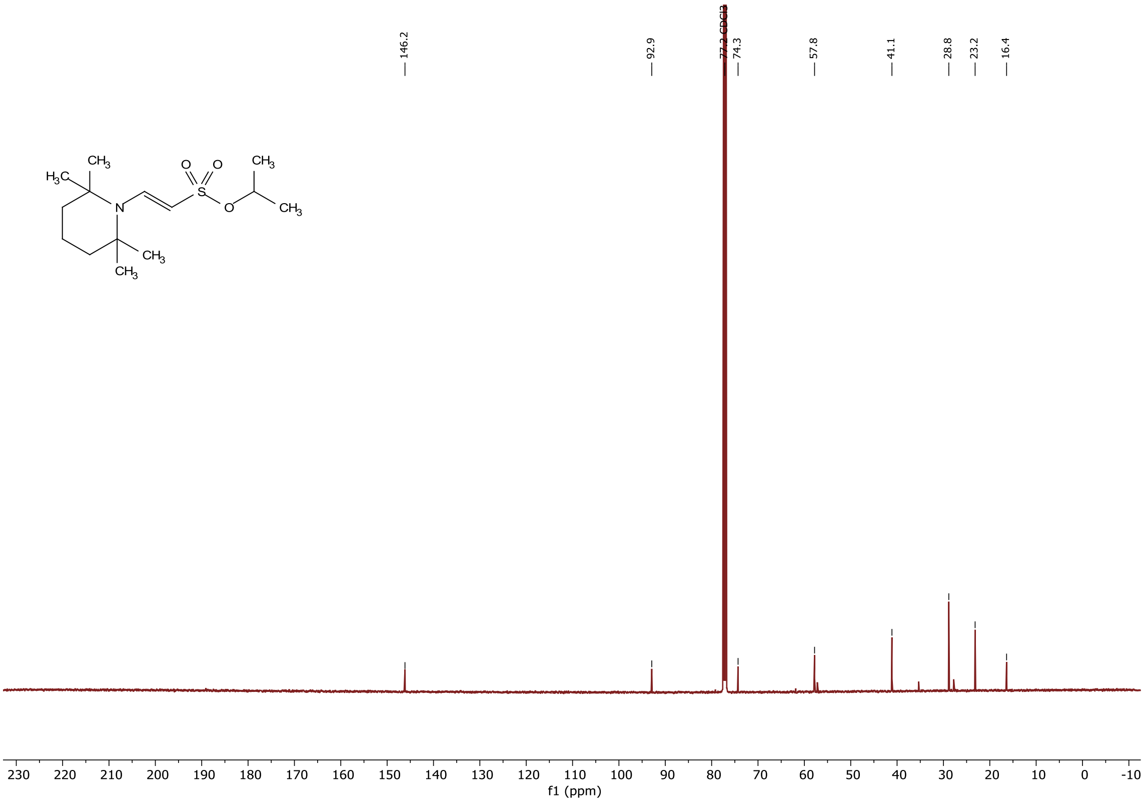

Figure S59: ^{13}C NMR spectrum (126 MHz, $CDCl_3$) of **4e**.

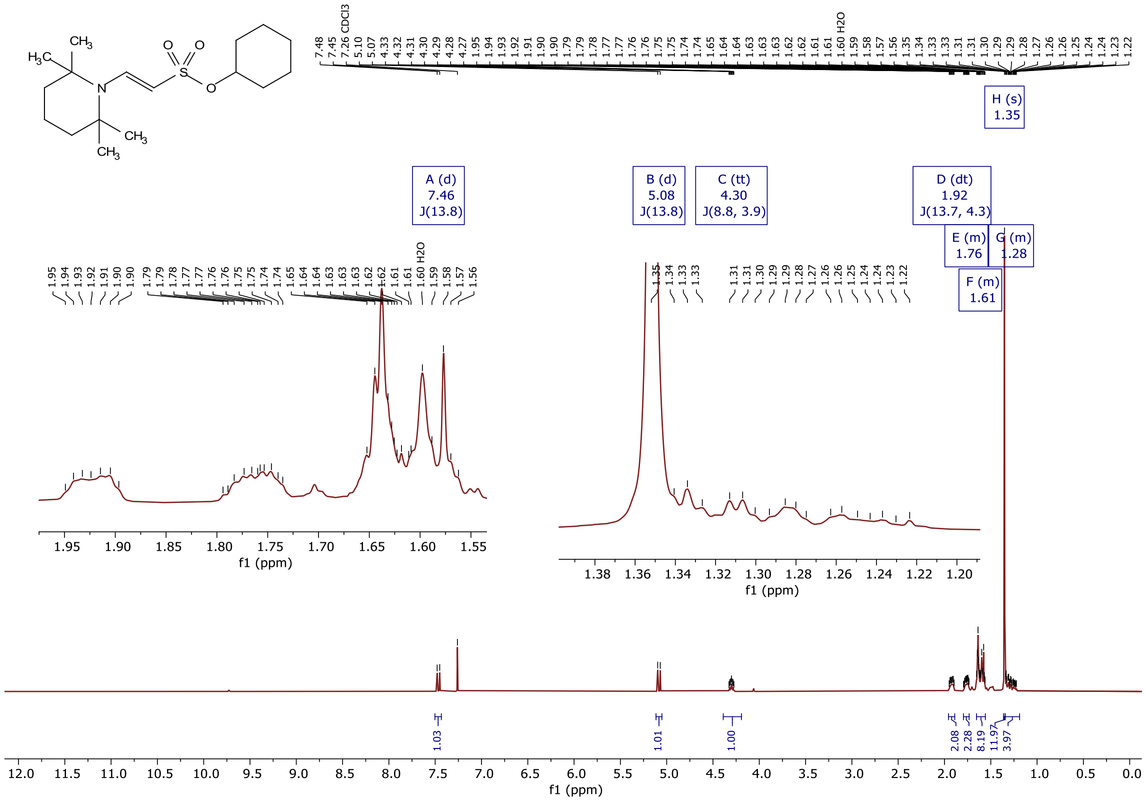

Figure S60: ^{1}H NMR spectrum (500 MHz, $CDCl_3$) of **4f**.

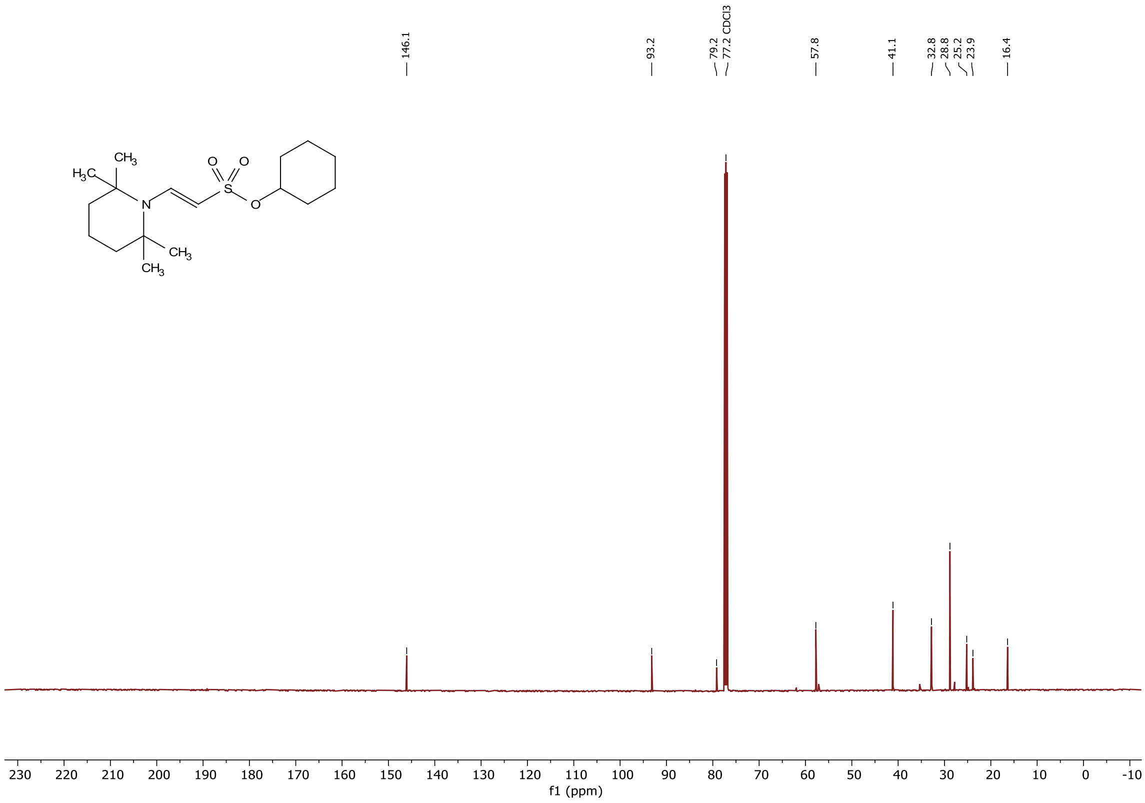

Figure S61: ^{13}C NMR spectrum (126 MHz, $CDCl_3$) of **4f**.

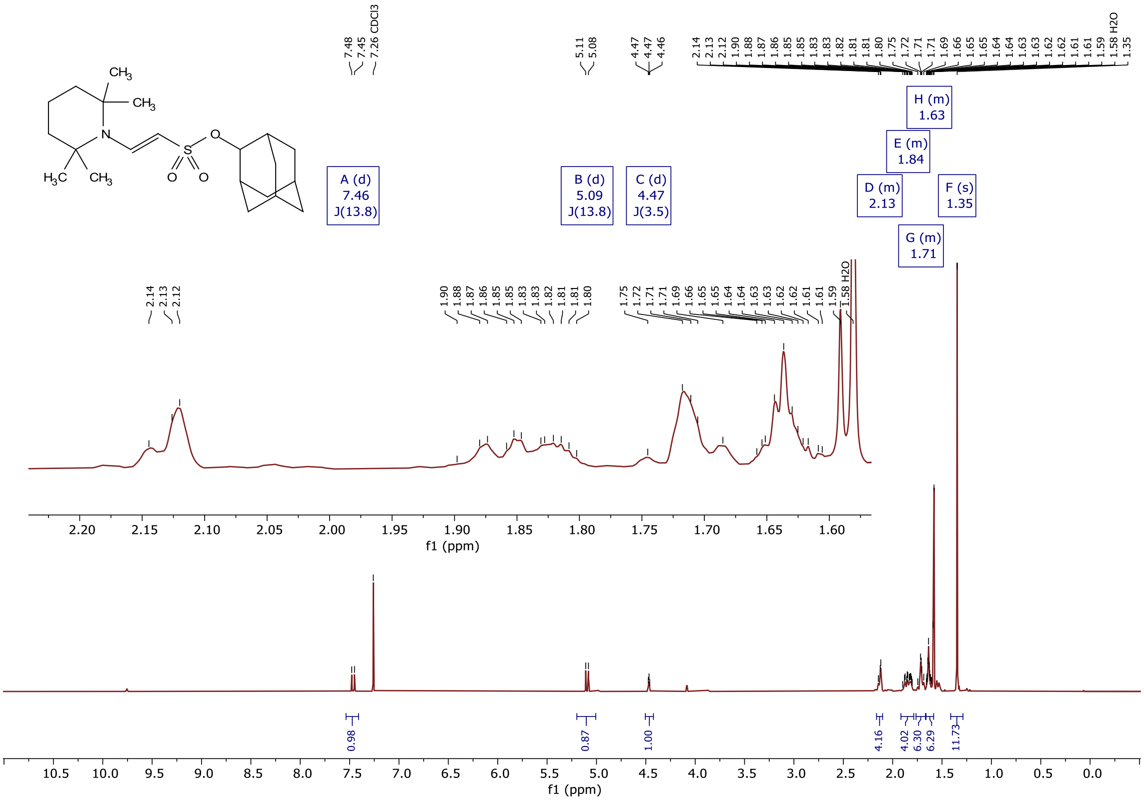

Figure S62: ^{1}H NMR spectrum (500 MHz, $CDCl_3$) of **4g**.

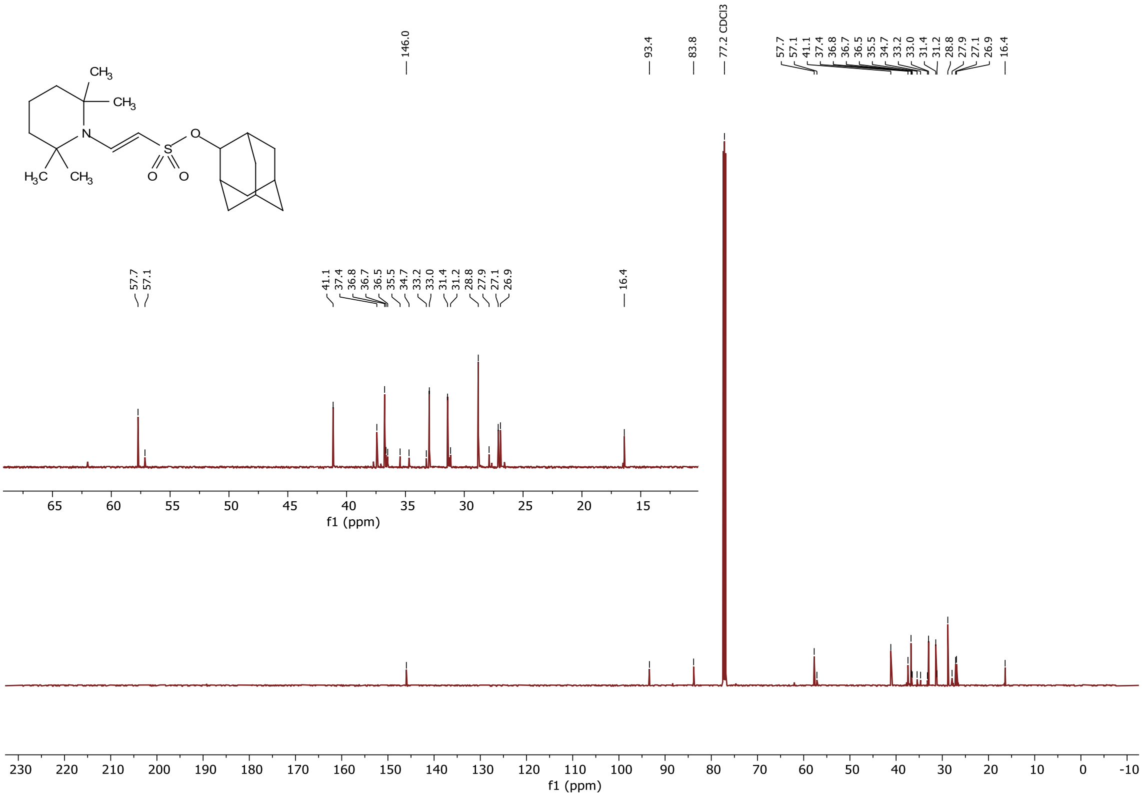

Figure S63: ^{13}C NMR spectrum (126 MHz, $CDCl_3$) of **4g**.

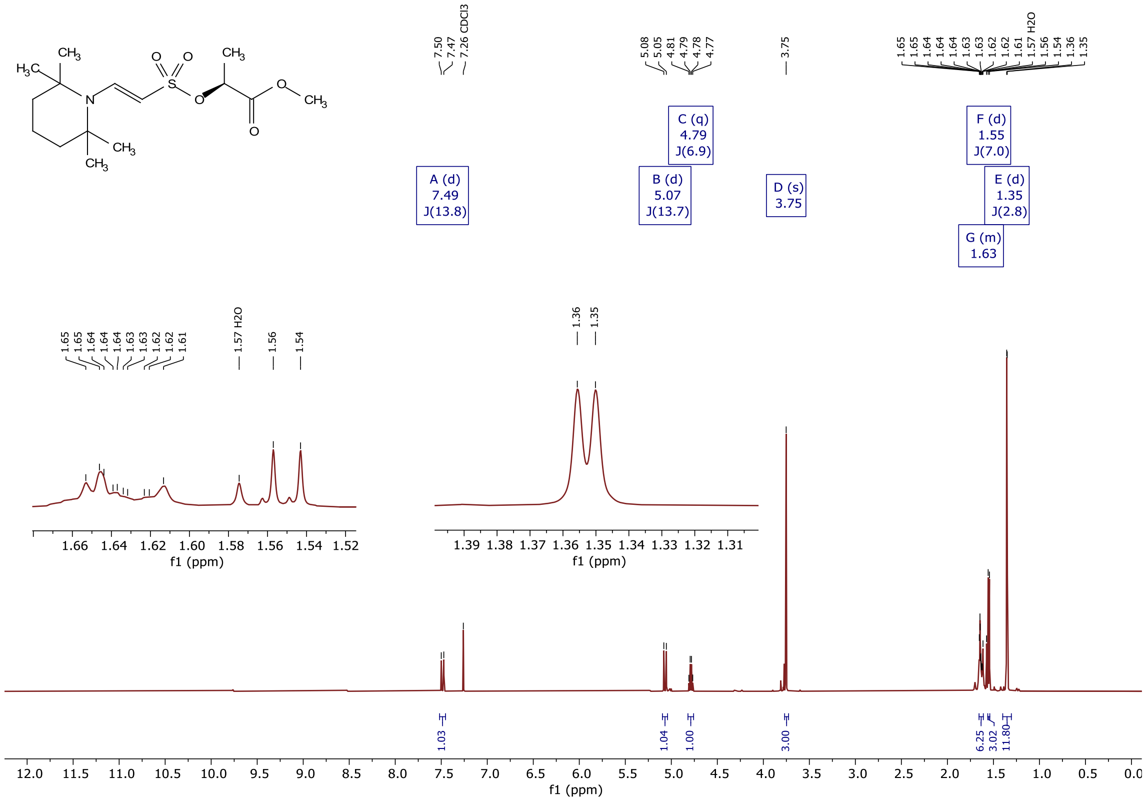

Figure S64: ^{1}H NMR spectrum (500 MHz, $CDCl_3$) of **4h**.

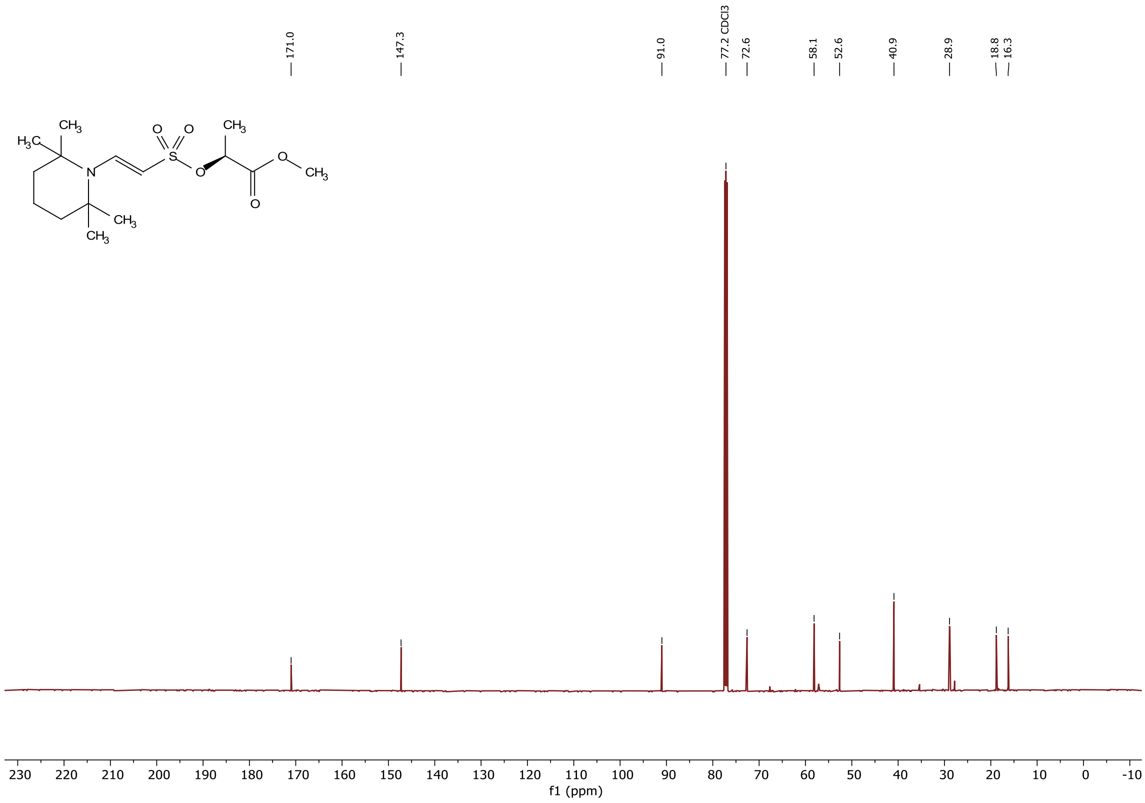

Figure S65: ^{13}C NMR spectrum (126 MHz, $CDCl_3$) of **4h**.

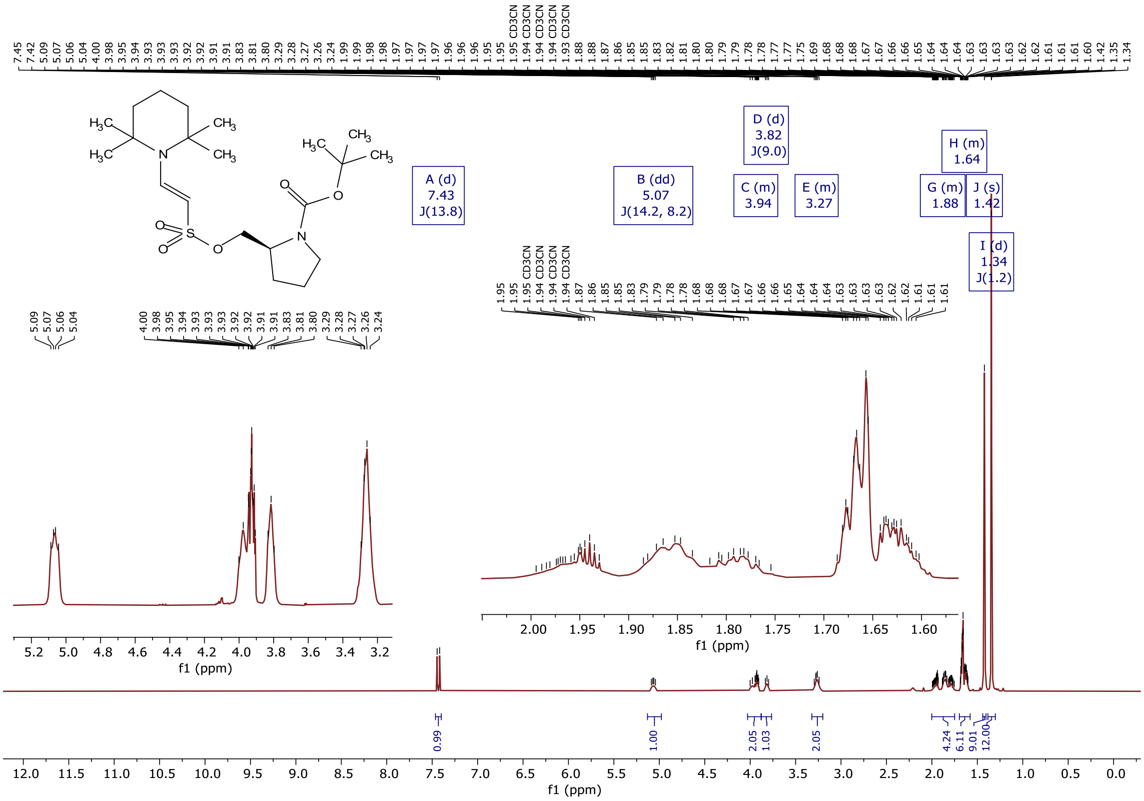

Figure S66: ^{1}H NMR spectrum (500 MHz, CD_3CN) of **4i**.

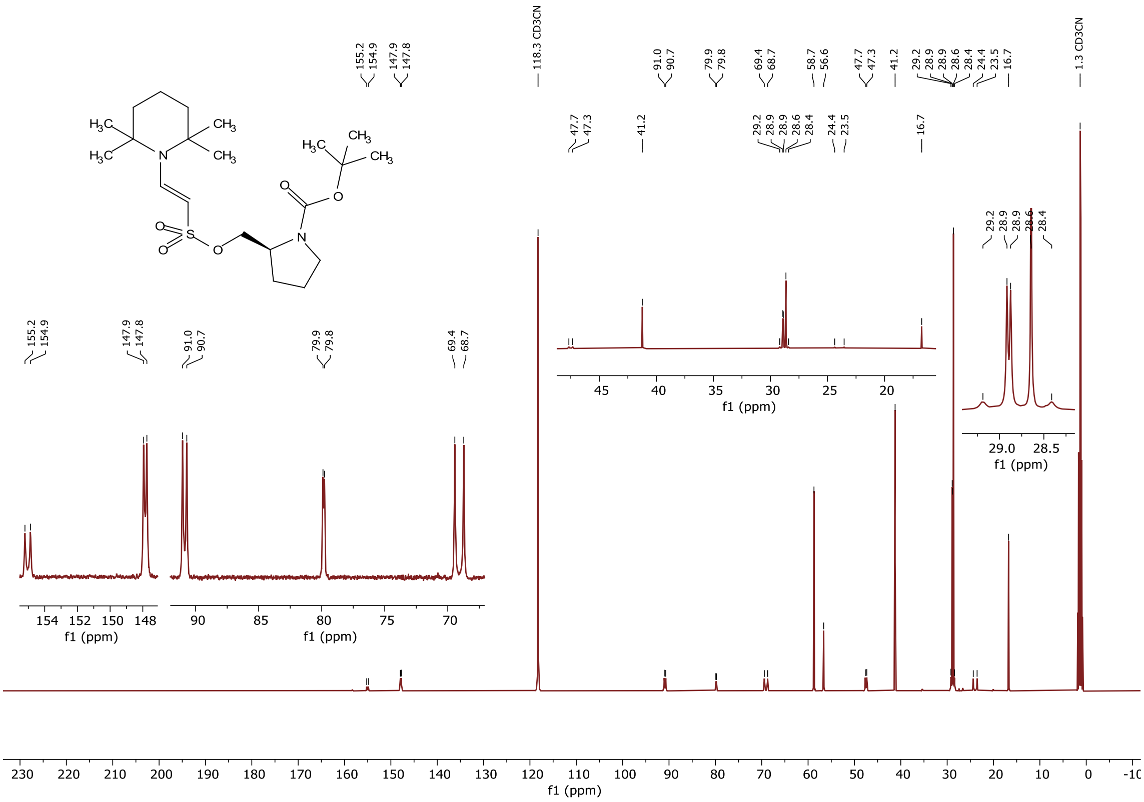

Figure S67: ^{13}C NMR spectrum (126 MHz, CD_3CN) of **4i**.

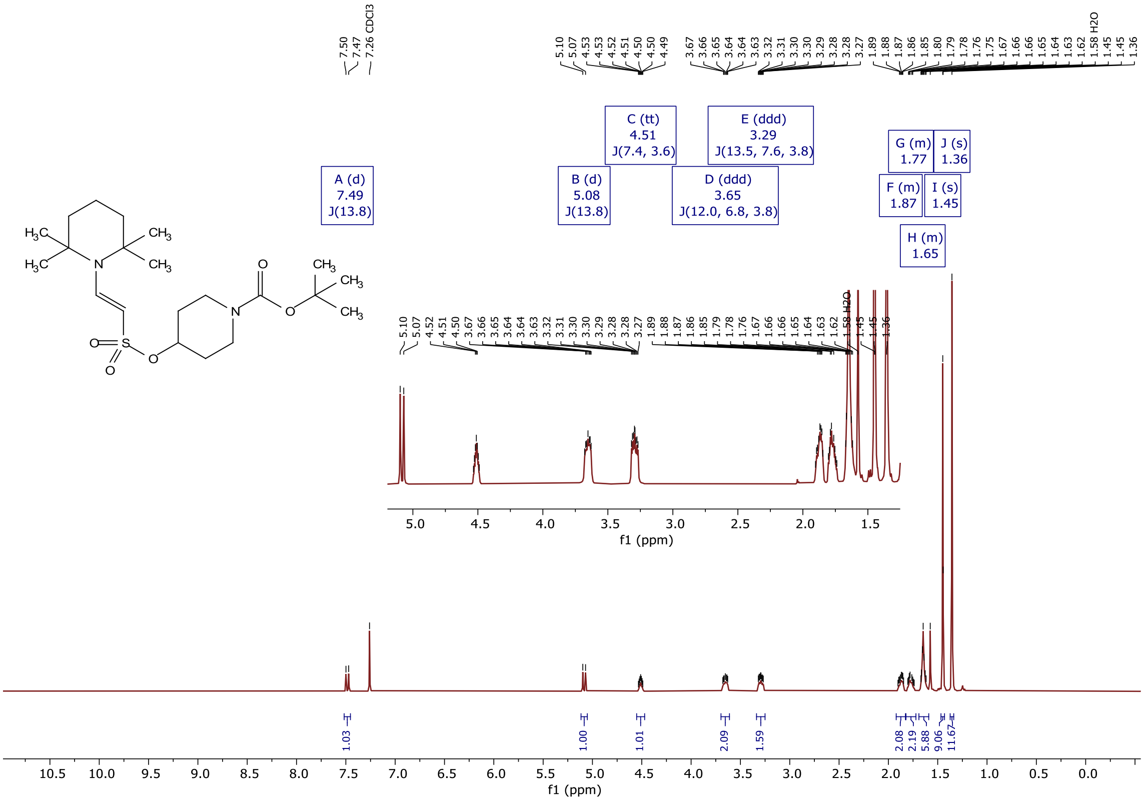

Figure S68: ^{1}H NMR spectrum (500 MHz, $CDCl_3$) of **4j**.

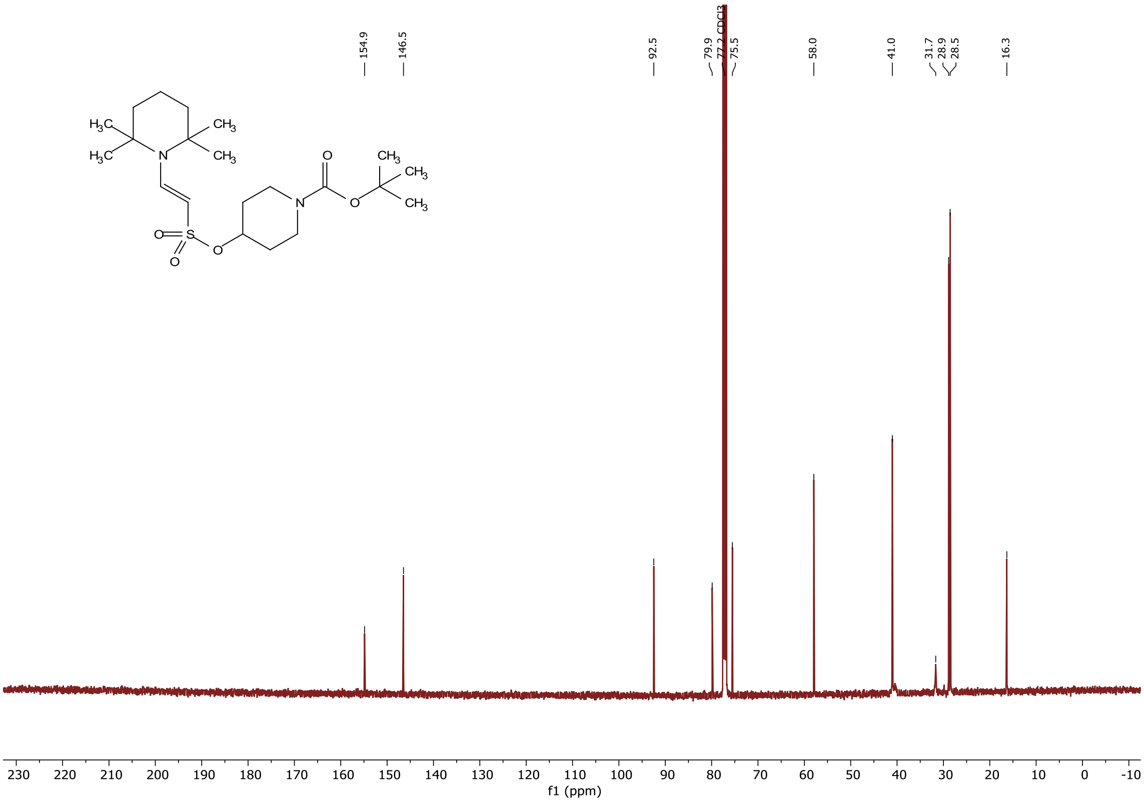

Figure S69: ^{13}C NMR spectrum (126 MHz, $CDCl_3$) of **4j**.

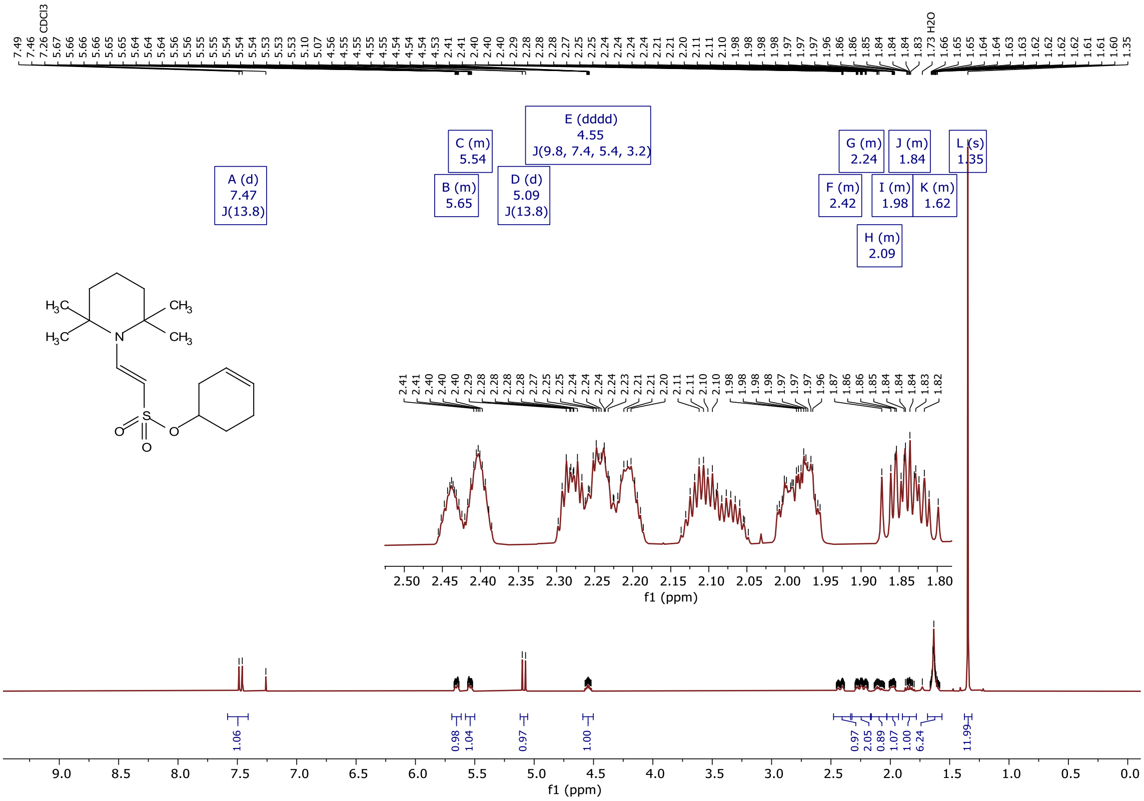

Figure S70: ^{1}H NMR spectrum (500 MHz, $CDCl_3$) of **4k**.

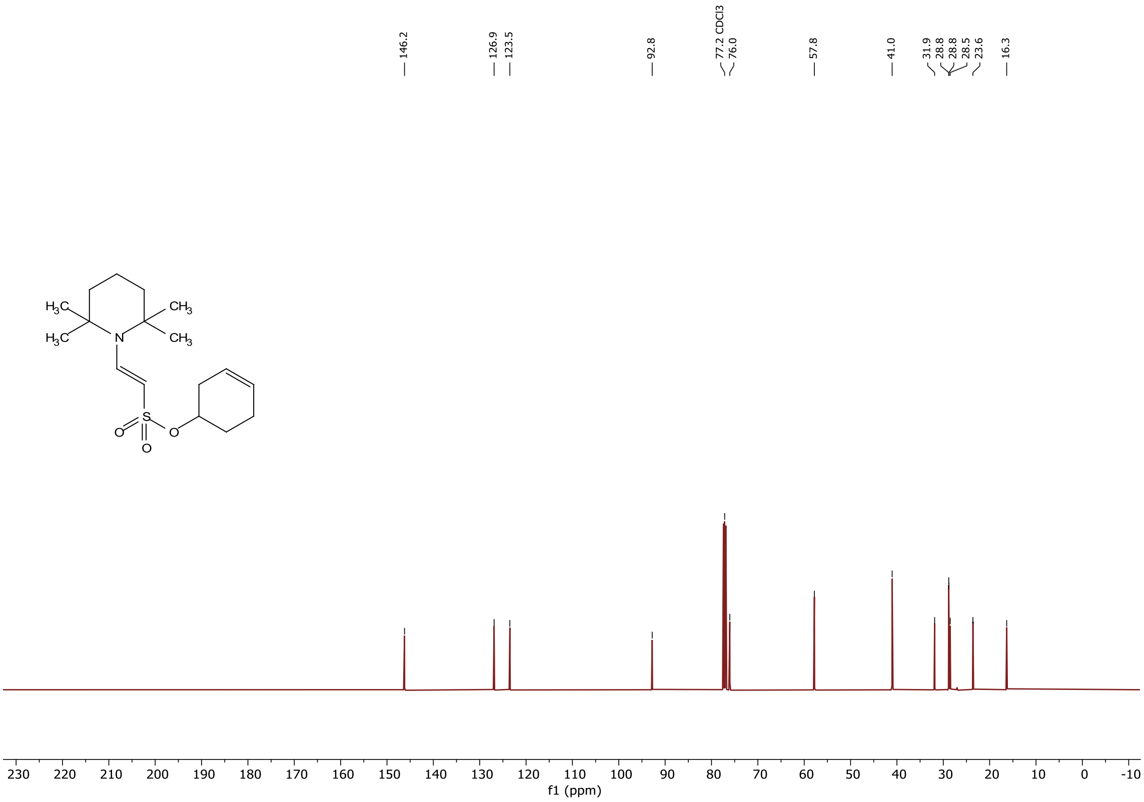

Figure S71: ^{13}C NMR spectrum (126 MHz, $CDCl_3$) of **4k**.

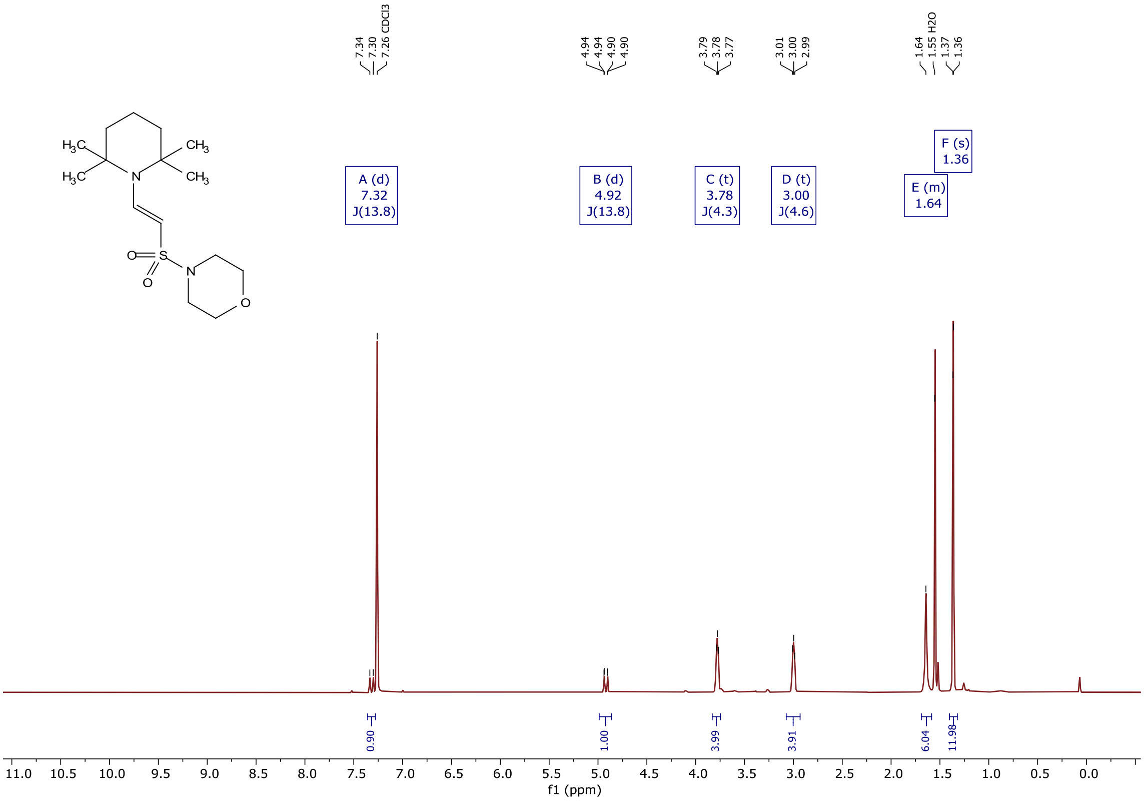

Figure S72: ^1H NMR spectrum (400 MHz, $CDCl_3$) of **5a**.

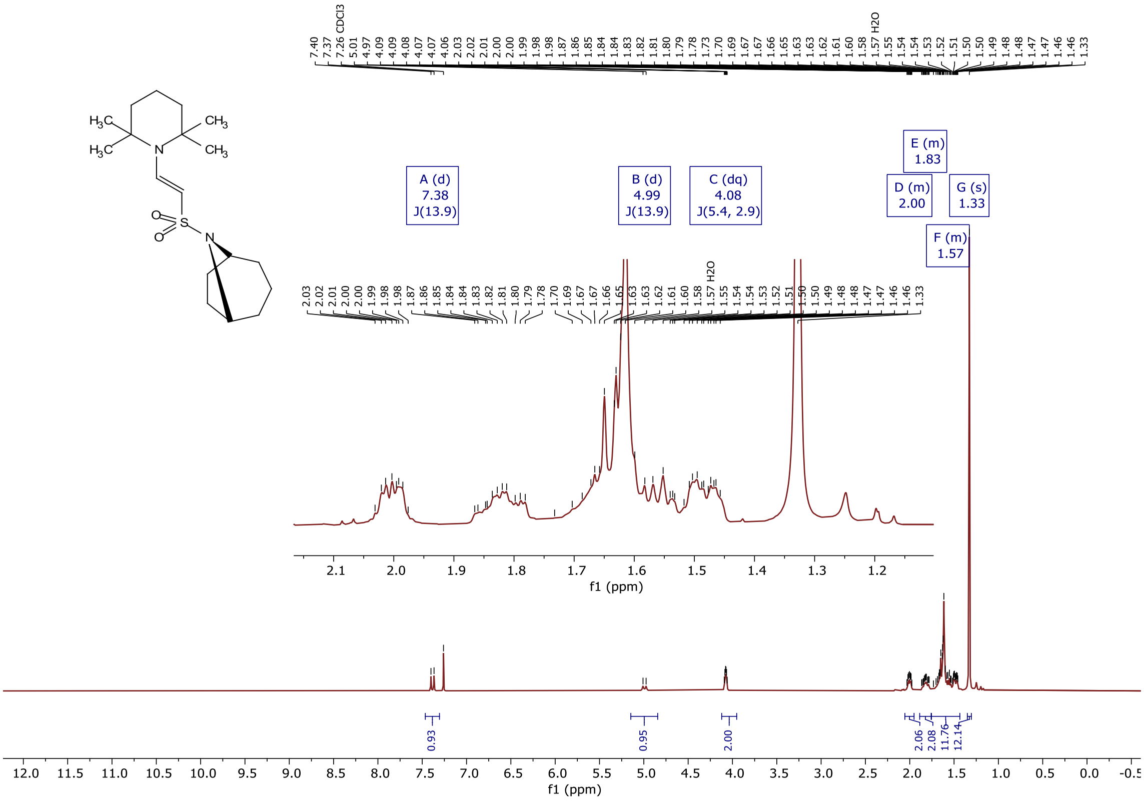

Figure S73: ^1H NMR spectrum (400 MHz, $CDCl_3$) of **5b**.

9 Author contributions

F.A.B.: Conceptualization, Investigation, Methodology, Data curation and analysis, Writing the original draft.

A.L.S.: Investigation: Supporting (optimization of reaction conditions)

C. K.: Investigation: Supporting (cyclic voltammetry measurements)

A. A. B.: Preliminary investigations

T. W.: Investigation: Supporting (crystallographic measurements and interpretation of X-ray data)

S.R.W.: Conceptualization, Funding acquisition, Project administration, Resources, Supervision, Writing the original draft.

10 References

(1) Gütz, C.; Klöckner, B.; Waldvogel, S. R. Electrochemical Screening for Electroorganic Synthesis. *Org. Process Res. Dev.* **2016**, *20*, 26-32.

(2) Komoda, M.; Nishina, Y. Electrochemical Production of Graphene Analogs from Various Graphite Materials. *Chemistry Letters* **2020**, *50*, 503-509.

(3) Ferguson, J. B. THE IODOMETRIC DETERMINATION OF SULFUR DIOXIDE AND THE SULFITES. *J. Am. Chem. Soc.* **1917**, *39*, 364-373.

(4) Miller, S. C. Profiling Sulfonate Ester Stability: Identification of Complementary Protecting Groups for Sulfonates. *The Journal of Organic Chemistry* **2010**, *75*, 4632-4635.

(5) (a) Mann, C. K. Cyclic Stationary Electrode Voltammetry of Some Aliphatic Amines. *Analytical Chemistry* **1964**, *36*, 2424-2426. (b) Mruthunjaya, A. K. V.; Torriero, A. A. J. Mechanistic Aspects of the Electrochemical Oxidation of Aliphatic Amines and Aniline Derivatives. *Molecules* **2023**, *28*, 471-502.

(6) (a) Blum, S. P.; Karakaya, T.; Schollmeyer, D.; Klapars, A.; Waldvogel, S. R. Metal-Free Electrochemical Synthesis of Sulfonamides Directly from (Hetero)arenes, SO_2, and Amines. *Angewandte Chemie International Edition* **2021**, *60*, 5056-5062. (b) Schneider, J.; Blum, S. P.; Waldvogel, S. R. Electrochemical Synthesis of Sulfonamides in Single-Pass Flow. *ChemElectroChem* **2023**, *10*, e202300456.
